# Supplementary material for: StellarPath: Hierarchical-vertical multi-omics classifier synergizes stable markers and interpretable similarity networks for patient profiling
Source: PLoS Comput Biol. 2024 Apr 12;20(4):e1012022. doi: 10.1371/journal.pcbi.1012022 (PMC11042724; doi:10.1371/journal.pcbi.1012022)
Supplement: S1 Text — Text file that provides extra information and details regarding specific aspects, operations and concepts of the designed methods. Plus, it includes the rationale behind specific operations and implementations. (PDF) [file pcbi.1012022.s001.pdf]

# SUPPLEMENTARY INFORMATION:

## Section 1.1:

StellarPath has been implemented in R and Python. R handles the data, while Python trains and uses the GCNs on the PSNs. StellarPath can be installed as an R package, has a function that automatically installs the Python module and has a user-friendly workflow to be applied to the data.

StellarPath begins its workflow with the "prepare_data" function, which processes the user's available data. This function requires a matrix indicating the patients' names and their classes. It currently accepts gene, miRNA, and lncRNA expression data in the form of separate continuous numerical matrices of read counts, and somatic mutation data in the form of a binary matrix (where a patient's profile has gene-level mutations indicated as ones and non-mutated genes as zeros). The function checks the input data to ensure downstream operations perform correctly, normalizes the omics, loads the pathway databases related to the species under study, and divides the data into two sets based on whether they are related to the training or testing (and validation) patients. The function returns a standardized list of objects for downstream analysis and the normalized omics.

The "analyse_training" function takes the result of the "prepare_data" function and analyzes the omics describing the training patients to identify significantly different molecules, pathways, and PSNs between the classes in comparison. Currently, the following analyses are implemented:

- gene expression data, presented as read counts, are normalized following the Law et al. [1] protocol. StellarPath identifies significantly different genes and canonical pathways, then it finds the class-signature pathway-specific PSNs.
- miRNA expression data, presented as read counts, are normalized following the Law et al. [1] protocol. StellarPath finds the significantly different miRNAs, tests the anti-correlation between each significant miRNA and its gene targets, finds the sets of target genes that are significantly anti-correlated with their miRNA and tests if every correlated set is also enriched by differentially expressed and stable targets between the two classes from the analysis, finally, it finds the class-signature PSNs representing significant target sets.
- lncRNA expression data, presented as read counts, are normalized following the Law et al. [1] protocol. StellarPath finds the significantly different lncRNAs, it tests the correlation between each significant lncRNA and its gene and miRNA targets, finds the sets of targets significantly correlated with a lncRNA and tests if every correlated set is also enriched by differentially expressed and stable targets between the two classes, finally, it finds the class-signature PSNs representing significant target sets.
- mutation data, presented as a binary gene-level matrix, are normalized employing a network-based propagation algorithm to address the sparsity of the matrix. StellarPath builds a network where genes from the matrix are represented as nodes, and edges between these nodes represent known physical interactions. These interactions are sourced from established databases such as BioGRID [2], STRING [3] or OmniPath [4]. For each patient sample, StellarPath maps the initial binary values of the genes to corresponding nodes in the network. It then applies a propagation, where these initial values (e.g., 1 for mutated, 0 for non-mutated) are spread through the network based on the interactions between nodes. The propagation is analogous to heat diffusion where the mutation status of one gene warms up its neighboring genes following a guilty-by-association principle. The result of this propagation is that each node in the network receives a continuous score. This score reflects the combined influence of its initial value and the values of its interacting neighbours. For instance, a non-mutated gene (initially set to 0) may receive a score closer to one if it is strongly connected to mutated genes, suggesting that it might be functionally related to the mutated state, even if it is not itself mutated. By this process, the sparse binary matrix, where most entries were 0, is transformed. Now, each gene (node) associated with a patient has a continuous score, reflecting its potential functional alteration based on its network context. The propagation has been widely used in scientific research and has been successfully applied to detect various biomarkers such as genes, pathways, miRNAs, lncRNA, protein

and somatic mutations. The algorithm implemented in StellarPath for the propagation is the Random Walk with Restart (RWR) [5]. Once the input matrix has been transformed, StellarPath identifies significantly different mutation-prone genes, determines if any of them fall into a significant pathway (including canonical pathways and target sets previously found analysing other omics) represented by a class-signature PSN, and if so, it adds the mutated gene to the meta-information of the pathway.

Testing the correlation (or anti-correlation) between one non-coding molecule and its targets means testing if the change in the expression of the non-coding molecule is significantly associated with the change in the expression of its targets. StellarPath tests the existence of the correlation (or anti-correlation depending on the relationship between the non-coding molecule and its targets) between a non-coding molecule (e.g. miRNA) and its targets (e.g. genes) using the empirical Cumulative Distribution Function (eCDF). StellarPath compares the eCDF obtained from the values of expression log fold change of the non-targets (control function) against that one of the targets. The eCDFs are compared using the Kolmogorov–Smirnov test, a type of statistical test that determines if two datasets differ significantly. If the test finds that the two distributions are significantly different, StellarPath concludes that the targets of the non-coding molecule are correlated with the non-coding molecule. This means that changes in the expression of the non-coding molecule are associated with changes in the expression of its target genes. This correlation can then be represented as a PSN where the similarity between patients is assessed using the expression values of the correlated molecules. This approach is commonly used to test the anti-correlation between miRNAs and their gene targets but can be applied to evaluate any relationship [6,7].

The "classify_testing" function uses the class-signature pathway-specific PSNs composed of training patients to train GCNs and to use the trained GCNs for predicting the class of the testing patients. The function returns the predicted classes of the testing patients, metrics to evaluate the classification performances of each triplet composed of GCN, PSN, and pathway, and metrics to evaluate the classification performances of the ensemble learning. We implemented the GCN with GraphSAGE algorithm using the Python library of StellarGraph. We used the generator GraphSAGENodeGenerator to feed a PSN and two variables defining the batch size equal to 50 for the training and a two-level model with 10 nodes sampled in the first layer and 5 in the second layer. We fed the patient's information into the model with the function generator.flow() which generated an object for the training set, one for the validation and one for the testing. We finalized our model by setting up the following parameters: each layer utilizes 32-dimensional hidden node features, bias is equal to true, dropout equal to 0.2, softmax as activation function, 200 epochs, early-stopping equal to 50 to stop the training once the loss did not decrease by 50 times continuously and Adam with an initial learning rate of 0.01 as optimizer.

The "enrichment_analysis" function performs a new analysis of the omics. Specifically, it works with all the patients and all the omics (without division into training and testing sets). It associates the testing patients to their predicted class, identifies the significant pathways, retains only the pathways that have been used previously by the "classify_testing" function, and tests the pathways for being represented by a significant PSN composed of all the patients. This ensures that the predictive molecules, pathways, and PSNs are also significant with the data provided by the testing patients. The function returns all the information, statistics, and plots related to the predictive and topologically relevant PSNs.

The "infer_new_data" function takes the output produced by the training (prepare_data, analyse_training, and classify_testing) made on one dataset in cross-validation setting and a new dataset of completely unknown patients. StellarPath uses what it learned from the first dataset to predict the class of each new unknown patient. As a result, the function returns the output of the functions "classify_testing" and "enrichment_analysis" referring to the new unknown patients.

Our implementation has two key characteristics. First, it reduces the interventions of the user who is mainly called to provide the data and run the workflow. Second, it allows the user to easily trace back the information that has been used to produce and find a predictive PSN. The "enrichment_analysis" function provides a link called "path2pathway" for each network, which can be used to retrieve

information related to the molecules and the pathway. As a consequence, StellarPath helps the user to understand why a PSN has been used to predict and why is biologically relevant.

## Section 1.2:

StellarPath accepts somatic mutation data in the form of a gene-level binary matrix. The rows are genes, the columns are patients and an entry of the matrix is 1 or 0. The entry (i,j) is 1 when the patient j has a mutation in the gene i, while 0 indicates the absence of mutation. The final matrix is sparse because each patient has few mutated genes with respect to the number of overall annotated genes.

We made the deliberate choice to work at the gene level rather than the variant level for somatic and germline mutations. This decision offers several advantages:

1. From a software perspective, a binary matrix is easier to store, process, analyze, and integrate than other formats. It simplifies the implementation and ensures efficient computation.
2. From an information perspective, the gene-level binary matrix has demonstrated its effectiveness in summarizing information about patients' mutations and exhibiting strong predictive power for disease and clinical status. Several papers in the literature have extensively used this data type in various applications, supporting its relevance and utility in genomics research.
   We would like to provide you with a list of papers that have employed the gene-level binary matrix for the analysis of somatic mutation data:

- https://doi.org/10.1371/journal.pcbi.1005573
- https://doi.org/10.12688/f1000research.26429.2 (netDx)
- https://doi.org/10.3389/fcell.2021.715275
- https://doi.org/10.1371/journal.pcbi.1010560
- https://doi.org/10.1016/j.crmeth.2023.100442
- https://doi.org/10.1214/15-AOAS836
- https://doi.org/10.1101/gr.120477.111
- https://doi.org/10.1371/journal.pcbi.1003054
- https://doi.org/10.1186/s12859-023-05319-8
- https://doi.org/10.1186/1752-0509-7-S2-S4
- https://doi.org/10.1186/1471-2105-16-S5-S3

These publications demonstrate the widespread use and acceptance of binary gene-level mutation matrices in genomics research and their potential to provide valuable insights into disease mechanisms and patient classification. We hope this explanation clarifies our rationale for considering somatic mutations as a sparse matrix represented at the gene level in StellarPath.

## Section 1.3:

The network-based propagation method is used to capture the likelihood of a gene being altered based on its interactions with one or more mutated genes. The intention is not to imply that all genes are carrying a mutation simultaneously but rather to consider the potential influence and impact of mutations in interacting genes. It works on the assumption that interacting genes are often involved in the same signaling cascade, pathway, or function; so if a gene is mutated then also its close genes are likely to be functionally altered. By considering the interactions between genes, the propagation aims to capture the broader context and potential impact of gene-gene relationships on the likelihood of alterations. While individual instances of co-mutation may be limited, the collective interactions and relationships among genes provide insights into altered functional roles and pathways.

The propagation approach has been widely and extensively used for transforming, normalizing, imputing, predicting, and integrating data in scientific research. Especially for biological purposes and with mutation profiles, the network propagation has been successfully used to detect driver mutational profiles, disease-specific subtypes and markers including genes, miRNA, lncRNAs, proteins and pathways.

We would like to provide you with a list of papers that have employed the network-based propagation with a gene-level binary mutation matrix:

- https://doi.org/10.1038/nmeth.2651 This paper by Ideker et al. is the most important research of network-based propagation applied to somatic mutation data represented as a gene-level binary matrix. It reviewed the propagation, proposed a workflow, and proved the benefits of the operation applied to somatic mutation data.
- https://doi.org/10.1371/journal.pcbi.1005573
- https://doi.org/10.1186/gm524
- https://doi.org/10.1093/bioinformatics/bty613
- https://doi.org/10.1089/cmb.2010.0265
- https://doi.org/10.1007/s12539-021-00475-y
- https://doi.org/10.12688/f1000research.26429.2 (netDx)

These publications demonstrate the widespread use and acceptance of the propagation technique applied on binary gene-level mutation matrices in research and their potential to provide valuable insights into disease mechanisms and patient classification.

## Section 1.4:

StellarPath divides the enriched pathways into four categories based on the prevalent types of expression and variance log fold changes observed in the significant molecules falling into these pathways. First, a pathway is considered activated (i.e. upregulated) by and for the case patients when it predominantly consists of molecules with positive expression log fold change (lFC) and negative variance lFC. Second, a pathway is considered activated by control patients when it exhibits a prevalence of molecules with negative expression lFC and positive variance lFC. Third, a pathway is considered inhibited by the case patients when it is primarily composed of molecules with negative expression lFC and negative variance lFC. Lastly, a pathway is considered inhibited by the control patients when it predominantly consists of molecules with positive expression lFC and positive variance lFC.

This type of categorization is not typically done by traditional methods that solely perform differential expression and pathway analysis because they operate under two key assumptions:

1) The differences captured between the two classes of patients are attributed to the case patients who are characterized by the condition under study. For example, if a molecule is found significantly downregulated, it means that the condition of the case patients inhibited the molecule.
2) The interpretation of the analysis results is left to the human expert, particularly when the two classes being compared represent distinct phenotypes or genotypes, neither of which is a healthy or control group (e.g. early vs late cancer stage, high vs low disease survival, etc …).

However, the patient similarity network paradigm cannot work under the same assumptions. In fact, while we do compare classes to select significantly different molecules and pathways, we also have to represent the patients' similarities and re-compare the classes on the PSN. This means that, with a classical categorization of pathways divided into up and down deregulated, the new comparison could lead us to get two different patient similarity networks. For instance, let us assume to represent how patients are similar according to an upregulated pathway (positive enrichment score produced by Broad Institute GSEA), then we could find two different PSNs::

1) The case patients exhibit a higher degree of similarity (because upregulating the pathway) among themselves compared to the control patients and the two classes are dissimilar.

2) The control patients exhibit a higher degree of similarity (because downregulating the pathway) among themselves compared to the case patients and the two classes are dissimilar.

During the implementation of StellarPath, we decided that we wanted to avoid producing PSNs that could potentially present both the topologies because would have been difficult to explain the biological reasons why a PSN had a specific topology instead of another one. As a consequence, we implemented our own pathway categorization based on the variance log fold change and the ad-hoc similarity measure to test whether the patient class that determines the overall molecular differences in a pathway is also the most cohesive class. For instance, a pathway considered activated by the case patients is tested to produce a PSN where case patients are cohesive, control patients are sparse and the two classes are dissimilar.

Section 1.5:

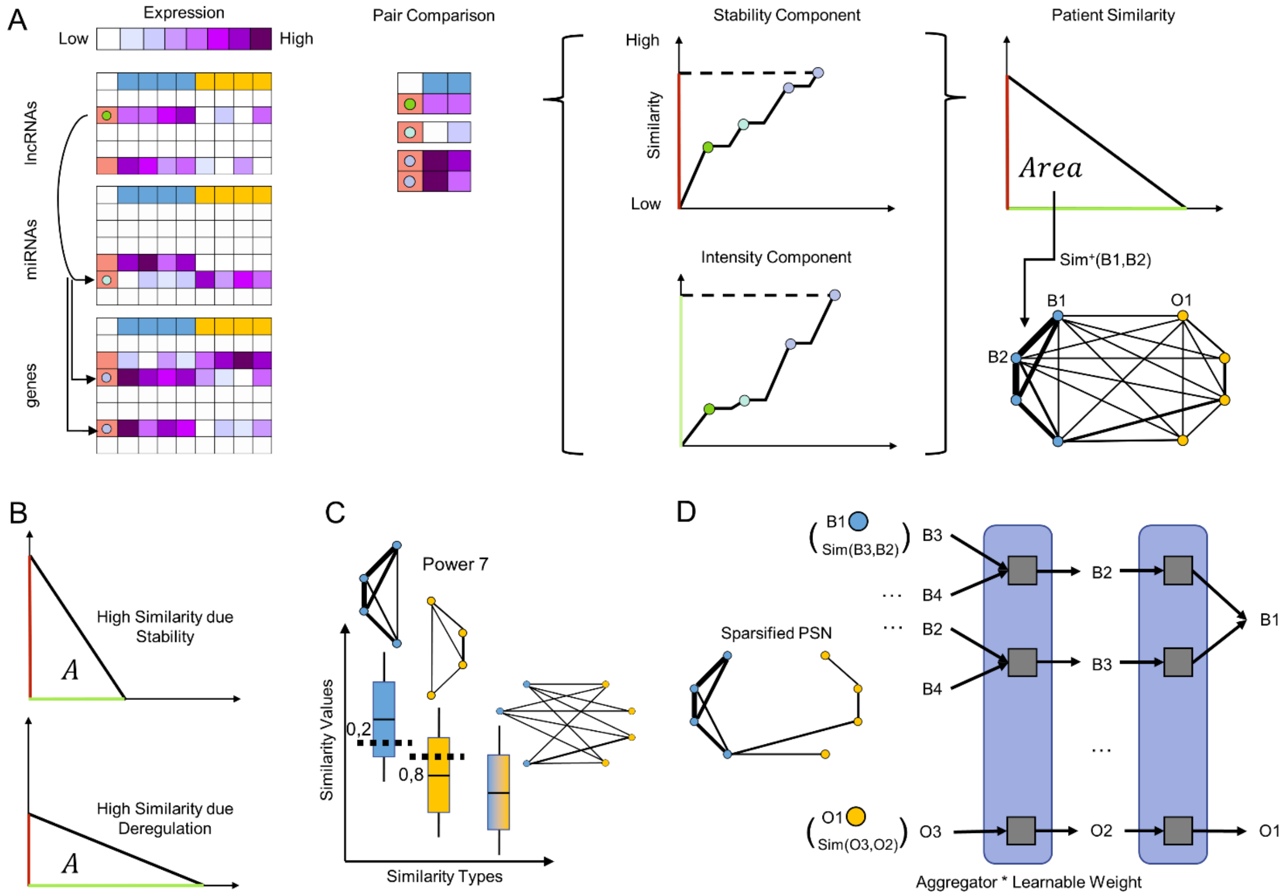


**(A)** It shows three omics produced by RNA sequencings describing the same patients with different molecules. StellarPath finds a significant deregulated pathway between blue and yellow patients, which involves a lncRNA targeting a miRNA that, in turn, targets two genes in anti-correlation. StellarPath gathers the expression values of the deregulated molecules in this pathway and computes the similarity measure. Since the pathway is consistently upregulated in blue patients due to the lncRNA and the gene targets, the similarity measure evaluates positively a high expression of the molecules. This example helps understand how the similarity between two blue patients is determined.The lncRNA has the same expression values in the two patients, so the component of the similarity which evaluates the stability is high. However, the lncRNA does not have very high values, so the component of the similarity which evaluates the intensity in expression is only moderate. The estimations are made also for all the other deregulated molecules and the final similarity score is equal to the area of the triangle defined between the two components. **(B)** The two components of the similarity measure do not always contribute equally to the final score. The final similarity can be interpreted differently based on the underlying components. In the first scenario, a higher stability component suggests that the similarity between two patients is more due to proximate

expression values rather than the expression level of these values. This could be indicative of patients exhibiting similarity within a homeostasis pathway. Conversely, in the second scenario, a dominant intensity component implies that both patients have elevated expression levels, though not necessarily in close proximity. This could be characteristic of patients sharing similarities in a strongly deregulated pathway relative to the contrasting group. **(C)** This part illustrates the comparison StellarPath performs to determine if a PSN is topologically significant for further analysis. Each boxplot contains similarity values between nodes (i.e., patients). For instance, the blue box only has similarities between blue patients. If the blue or yellow box is higher than the others, the PSN is considered signature of that cohesive class. Here, the 20th percentile of the blue distribution is above the 80th percentile of the other two, giving the PSN a Power of 7. (D) This section describes the GCN training. The GCN refines each node's representation in the PSN by integrating information from its neighboring nodes at varying distances.

## Section 1.6:

We compare our similarity measure against the Euclidean similarity, Pearson Correlation and Cosine similarity. In this example, we build a dummy count matrix describing the expression of three genes in ten patients. The first four patients belong to the case class and the last six patients belong to the control class. We assume that all the three genes belong to the same pathway, we compute the differential expression analysis implemented in StellarPath, we recognize that the three genes are downregulated. However, the pathway is considered upregulated for the control patients because they express the genes with more stable and precise expression values than the case patients. Next, we compute the similarity measures, and we rank how much each patient is similar to the fifth patient (first control patient) according to each measure. Finally, we make practical observations and conclusions.

```
#Input data ----
#Similarity measures in comparison
sim_names=c("StellarPath","Euclidean","Pearson",”Cosine”)

# Let's define a dummy count matrix
m = cbind(c(1,3,5),
          c(10,13,15),
          c(20,23,25),
          c(50,51,52),
          c(57,60,62),
          c(58,61,63),
          c(64,61,57),
          c(60,63,65),
          c(65,68,70),
          c(70,73,75))

# genes PAT1 PAT2 PAT3 PAT4 PAT5 PAT6 PAT7 PAT8 PAT9 PAT10
# GENE1    1   10   20   50   57   58   64   60   65    70
# GENE2    3   13   23   51   60   61   61   63   68    73
# GENE3    5   15   25   52   62   63   57   65   70    75

# Assign column and row names to the matrix
# Columns are patients
# Rows are genes
colnames(m)=paste("PAT",seq(1,ncol(m)),sep="")
rownames(m)=paste("GENE",seq(1,nrow(m)),sep="")
# Patient's classes
# We assume that the first 4 patients belong to the CASE class and the last 6 to the CTRL class
groups=c(rep("CASE",4),rep("CTRL",6))

# Find significantly different molecules between groups
find_SDR(m,groups)

# genes ExplogFoldChange stability activity_CASE activity_CTRL stable_for significant sign_type
# GENE1     -2.047306 -3.480573            15            62       CTRL        TRUE         2
# GENE2     -1.784271 -4.521966            18            62       CTRL        TRUE         2
# GENE3     -1.678072 -3.000928            20            64       CTRL        TRUE         2
# All the genes are significatly downregulated (according to the standard interpretation of a DE
analysis) but they are more stably expressed in the control case.
```

```
# As such, StellarPath attributes the pathway regulation to the control class which is actually upregulating the molecules.
# We calculate the patient similarity according to StellarPath measure, Euclidean distance and Pearson Correlation.
# We are going to analyse the similarities related to patient 5 (the first control patient). So we are going to rank the patients
# from the most similar to dissimilar from patient 5.
# Calculate similarity matrix using StellarPath's similarity measure
sim_m0=build_PSN(m)
# Apply a column rank
sim_m0=apply(-sim_m0,2,rank)
# Calculate similarity matrix using Euclidean distance
dist_m=as.matrix(dist(t(m),upper = TRUE,diag = TRUE))
sim_m1=1-(dist_m/max(dist_m))
diag(sim_m1)=1
# Apply a column rank
sim_m1=apply(-sim_m1,2,rank)
# Calculate similarity matrix using Pearson correlation
sim_m2=cor(m)
# Apply a column rank
sim_m2=apply(-sim_m2,2,rank,ties.method="min")
# Calculate similarity matrix using Cosine similarity
sim_m3=lsa::cosine(m)
sim_m3=apply(-sim_m3,2,rank)
# Let us compare how each patient is similar to the patient 5 based on the different measures
sim_bench=cbind(sim_m0[,5],sim_m1[,5],sim_m2[,5],sim_m3[,5])
colnames(sim_bench)=sim_names
sim_bench=as.matrix(sim_bench)
# Patients StellarPath Euclidean Pearson Cosine groups
# PAT1          10        10       8       10    CASE
# PAT2           9         9       1       9     CASE
# PAT3           8         8       1       7     CASE
# PAT4           7         6       8       6     CTRL
# PAT5           1         1       1       1     CTRL
# PAT6           2         2       1       2     CTRL
# PAT7           5         4      10       8     CTRL
# PAT8           3         3       1       3     CTRL
# PAT9           4         5       1       4     CTRL
# PAT10          6         7       1       5     CTRL
```

Differences:

1) PAT5 is correctly most similar to itself according to all the measures, However Pearson Correlation considers PAT5 similar in the same way to PAT2, PAT3, PAT6, PAT8, PAT9 and PAT10.
2) PAT6 is correctly the second most similar patient to PAT5 for both StellarPath, Euclidean and Cosine similarity measures.
3) According to StellarPath, PAT8 and PAT9 are more similar to PAT5 than PAT7. According to the Euclidean similarity, PAT7 and PAT8 are more similar to PAT5 than PAT9. According to Pearson Correlation, PAT7 is the most dissimilar to PAT5 because is anti-correled with respect PAT5. According to the Cosine similarity, PAT8 and PAT9 are more similar to PAT5 than PAT7.
   a. StellarPath attributes a higher similarity to PAT8 and PAT9 because the pathway is upregulated, so higher is the average of the genes' values between two patients (PAT5-PAT8, PAT5-PAT9) and higher is their similarity. StellarPath attributes a lower similarity to PAT7 because is anti-correlated and neither very close or high with respect PAT5.

b. Euclidean similarity considers PAT7 more similar to PAT5 than PAT9 because the first two patients express the same genes with closer values.
c. Cosine similarity penalizes a lot PAT7 because of its anti-correlated profile with respect PAT5, in fact PAT7 is more dissimilar to PAT5 than PAT3 and PAT4 which are case patients.

4) According to StellarPath, PAT10 is the least similar control patient to PAT5 because the two patients have very distant values in the same genes. On the contrary of what happens with PAT8 and PAT9, PAT10 is not considered more similar to PAT5 than PAT7. The reason is that, StellarPath similarity measure is formulated as area of triangle where closeness and highness (in a upregulated pathways) of genes' expression values are equal contributors. As consequence, there is a limit to how much the high values of PAT10 can push the similarity with PAT5. The closeness of the genes' values of PAT7 with that ones of PAT5 has been considered more important than the highness of genes' values of PAT10. However still, StellarPath considers PAT10 more similar to PAT5 than all the case patients (PAT1,2,3,4) because the latter individuals have all expression profiles with lower values than PAT5. While according to the Euclidean similarity, PAT4 is more similar to PAT5 than PAT10 because the first two have more close expression values in the same genes.

Conclusions:

1) Pearson or correlation measures are not adequate to assess the similarity between patients described with omics data. They evaluate the similarity between two patients based on how much is similar the relationship (i.e. trend) of their molecules but as we showed in the example, two patients could have completely different expression profiles and being considered strongly correlated. Correlation measures have been undoubtedly used to find correlated molecules between patients. A popular method that exploits this principle is WGCNA to build gene-gene correlation networks. However, the usage of correlation measures to assess the similarity between patient's expression profiles has never been officially proven valid. It could be theorized and discussed that, correlation measures could be useful when there is the need to compare expression profiles which have different intensities of count values but this issue is normally address by normalization techniques like TMM or the relative expression (also to allow the proper operation of a differential expression analysis).
2) StellarPath correctly associates PAT5 to the control patients, while separating PAT5 from case individuals.
3) Euclidean and Cosine similarity are not designed to value how much two patient's profiles have high expression values in a upregulated pathway or low expression values in a downregulated pathway. As negative consequences, Euclidean similarity considers PAT5 more similar to PAT4 than to PAT10, and Cosine similarity considers PAT7 less similar to PAT5 than PAT3 and PAT4.
4) Euclidean similarity is not designed to weigh negatively profiles having opposite trends like PAT5 and PAT7.
5) Cosine similarity is not designed to balance the closeness and the trend of values between two profiles like PAT5 and PAT7.

We explained the reasons why we decided to implement our own similarity measure, we described its advantages, and we provided an example of application. Undoubtedly, it is challenging to prove that one similarity measure is better than another one in all the aspects, terms, and situations. However, we believe that our similarity measure can be considered easier to understand and to interpret than other classical similarity measures that have not been developed for comparing patient's molecular profiles.

## Section 1.7:

The Pearson Correlation measures the linear relationship between two variables, it has been extensively used to evaluate how much genes are correlated among patient's expression profiles, but it is not suitable for assessing the similarity between two samples' gene expression profiles, especially in the context of sequencing data. The main reasons are the following:

1. Pearson Correlation is sensitive to outliers. In gene expression data, a single gene with an unusually high or low expression can significantly affect the correlation coefficient. This can lead to misleading conclusions about the overall similarity between the two profiles.

2. Pearson Correlation assumes a linear relationship between the two variables. Gene expression data often exhibit non-linear relationships, and the Pearson Correlation may not capture these complexities. This can result in an underestimation of the true relationship between two expression profiles.

3. Pearson Correlation is sensitive to the scale of the data. If the expression levels of genes in one profile are consistently higher or lower than in the other, the Pearson Correlation may indicate a strong correlation even if the patterns of expression are not similar. This can be problematic when comparing expression profiles across different conditions or treatments where the scale easily varies.

4. Pearson Correlation only considers the direction of the relationship, not the magnitude. Two profiles may have the same trend but different levels of expression. Pearson Correlation would consider these profiles similar, ignoring the potentially significant biological differences in expression levels.

The Pearson Correlation is mainly suited for assessing the correlation between genes. It became popular for this application when WGCNA was published. The main reason is the following:

1. In pathways, genes often work together. If two genes are part of the same biological process, their expression levels may change together in a coordinated way across different conditions or patient profiles. Pearson Correlation helps identify co-expressed genes. In fact, Pearson Correlation is used to build gene co-expression networks. By identifying pairs of genes that are highly correlated.

In conclusion, the popularity of Pearson Correlation in measuring how much two genes are correlated among patient profiles is due to its mathematical simplicity, interpretability, and applicability to the specific task of identifying linear relationships between genes. However, we believe that Pearson Correlation wrongly inherited the capability of assessing properly the similarity between patients.

## Section 1.8:

Let the $k$-hop neighbours of a node $v_i$ be defined as the set of nodes that are $k$ hops distant from $v_i$. This set is denoted as $N_k(i)$ and is formulated as follows:

$$N_k(i) = \{v_j \mid i \neq j, min(\text{sp}\ (i,j), K) = k, \forall v_j \in \mathcal{V}\}$$

where, $\text{sp}\ (i,j)$ is the shortest path between $v_i$ and $v_j$, while $K$ represents the maximum number of hops. Secondly, GCN learns embeddings so let an embedding for a node $v$ be defined as $h_{\text{v}}$ where $H \in R^{|V| \times F}$ with $F$ that is the dimension of nodes' representations (i.e. length of the vector containing the aggregated and compressed information).

We decided to use a specific implementation of the GCN which is called GraphSage, a framework for inductive node embedding.

Given the learnable matrices of weights $\text{WE}^k, \forall k \in \{1, \dots, K\}$ and a non-linear activation function such as the rectified linear unit: $\text{ReLU}\ (\cdot) = \max(0,\cdot)$ defined with $\sigma(\cdot)$, the GraphSAGE algorithm can be presented as follows:

$\text{h}_i^0 \leftarrow \text{x}_i, \forall i \in \{1,2,3, \dots., |V|\}\ s.t. v_i \in \mathcal{V}$

$for\ k = 1 \dots K\ do\ \{$

$for\ i \in \{1,2,3, \dots., |V|\}\ do\ \{$

$\text{h}_i^k \leftarrow \sigma\big(\text{WE}^\text{k} \cdot \text{MEAN}\ (\{\text{h}_i^{k-1}\} \cup \{\text{w}_\text{ij}\text{h}_j^{k-1}, \forall \text{V}_\text{j} \in \mathcal{N}_k(\text{i})\}\big)$

}

$\mathrm{h}_i^k \leftarrow \frac{\mathrm{h}_i^k}{\|\mathrm{h}_i^k\|_2}$

}

$\mathrm{z}_i \leftarrow \mathrm{h}_i^K$

The final embedding $\mathrm{z}_i$ is then converted into the probability of associating the $i$-th node into one of the two classes. For such task, the softmax function converts the embedding (vector of real numbers describing the attributes of a node) into a probability and is defined as follows:

Given the embeddings $Z = \{z_1, z_2, \ldots, z_n\}$ representing the training patient nodes, the softmax function computes the probability for each node:

$$pr_i = \frac{e^{z_i}}{\sum_{j=1}^{n} \mathrm{e}^{\mathrm{z_j}}}$$

By applying the softmax function, the outputs of the GraphSage model are transformed into a probability distribution, allowing to retrieve the class as follows:

$$pry_i = \begin{cases} if\ pr_i < 0.5\ then\ CL1 \\ if\ pr_i > 0.5\ then\ CL2 \end{cases}$$

The loss function called categorical cross-entropy is then used to calculate the loss between the predicted classes and the true ones, the Adam optimization algorithm is called to update the model's weights for minimizing the loss and the workflow is repeated with the new weights.

## Section 1.9:

Metrics like accuracy, precision, recall and F1 score are commonly used. However, they have significant limitations when evaluating the classification of two imbalanced classes. This means that they are unsuitable for evaluating our binary classifications performed on TCGA datasets.

The Matthews correlation coefficient (MCC) is a metric used to assess the performance of a binary classification model. It is calculated as follows:

MCC = (TP*TN – FP*FN) / √(TP+FP) (TP+FN) (TN+FP) (TN+FN)

where TP is the number of true positives, TN is the number of true negatives, FP is the number of false positives, and FN is the number of false negatives. The MCC ranges from -1 to 1, with -1 representing a completely incorrect classification and 1 signifying a completely accurate classification.

MCC is considered more reliable than other metrics in evaluating binary classification tasks [3,4]. It produces a high score only if the model scores well in all the four confusion matrix categories (true positives, false negatives, true negatives, and false positives), proportionally both to the size of positive objects and the size of negative objects in the dataset. For this reason, it is considered better than accuracy, precision, recall and F1 score [8,9]

Accuracy measures the proportion of correct predictions out of the total number of objects in the dataset. However, accuracy can be misleading when the dataset is imbalanced, meaning that one class has significantly more objects than the other. In such cases, a classifier that predicts correctly the biggest class can achieve high accuracy, even if it performs poorly on the smallest class.

F1 score is the harmonic mean of precision and recall. Precision measures the proportion of true positives among all positive predictions, while recall measures the proportion of true positives among all actual positive objects. F1 score balances precision and recall and is a good metric when the classes are balanced. However, F1 score can be misleading when the dataset is imbalanced, as it does not take into account true negatives, false negatives and false positives.

In contrast, MCC produces a high score only if the predictions are evaluated positivetely by all the categories, proportionally both to the size of positive elements and the size of negative elements in the dataset. MCC is a robust metric that summarizes the classifier performance in a single value, being suitable for both balanced and imbalanced positive and negative classes.

To demonstrate the advantages of MCC over other metrics, Davide Chicco et al. provide R scripts on GitHub. The scripts generate R plots for the comparison between MCC, accuracy, and F1 score.

In StellarPath, our Table B in S1 Tables presents the numbers of the patients in each dataset, including the size of each class that we compared. Notably, in many datasets, the smallest class is approximately half the size of the largest class. Given the prevalence of imbalanced datasets, we firmly consider using the MCC as the main metric to evaluate the predictions made by the classifiers.

## Section 1.10:

In the skin melanoma (SKCM) dataset, StellarPath identified pathways enriched in late stage cancer patients that elucidate the tumor's mechanisms. The negative regulation of the canonical WNT pathway contributes to tumor progression. Metastatic melanomas exhibit strong WNT5A expression, and a lower level of nuclear β-catenin compared to primary melanomas. In other terms, the lack of nuclear β-catenin (indicative of suppressed canonical WNT signaling) and high levels of cytoplasmic WNT5A (indicative of activated non-canonical WNT signaling) favors the melanoma progression [1] . Accordingly, the upregulation of the Cell Shape and Cell-Substrate Adhesion pathways, which are central to the epithelial to mesenchymal transition (EMT), enables primary melanoma cells to transition into a more aggressive, metastatic form [2]. Consequently, the upregulation of the Notch Signaling pathway is associated with this development [3], further promoting tumor growth, metastasis, and immunosuppression. Most importantly, StellarPath identified deregulation in hsa-miR-206 and its gene targets. This microRNA is a marker of aggressive melanoma progression and an unfavorable prognosis [4]. Collectively, these pathways depict a progression of the patient's disease from early to late stages, characterized not merely by growth in size, but also by changes in biological behavior and interactions, leading to increased invasiveness.

[1] https://www.ncbi.nlm.nih.gov/pmc/articles/PMC7402324/

[2] https://www.ncbi.nlm.nih.gov/pmc/articles/PMC4346328/

[3] https://surgexppathol.biomedcentral.com/articles/10.1186/s42047-019-0052-9#Sec3

[4] https://www.ncbi.nlm.nih.gov/pmc/articles/PMC4440132/

Finally in the pancreatic adenocarcinoma (PAAD) dataset, a notable finding is the upregulation of the Epithelial Mesenchymal Transition (EMT) pathway, which plays a key role in pancreatic cancer progression [1,2,3]. This pathway leads to the transformation of cancer cell phenotype, making them more invasive and enhancing their motility and invasive capabilities. Such changes are crucial for rapid metastatic progression in pancreatic ductal adenocarcinoma, particularly in later stages of the disease. The upregulation of the Cell Adhesion pathway is nicely and closely linked [4] to EMT. The loss of cell polarity and cell-to-cell adhesion, hallmarks of EMT, initiates a series of changes, including the loss of apical-basal polarity and cytoskeleton reorganization. These alterations result in increased cell motility, further augmenting the metastatic potential of cancer cells in advanced pancreatic cancer. In connection with the deregulation of the EMT and Cell Adhesion pathways, the Focal Adhesion pathway is also altered [3,5]. The signaling cascades regulated by the extracellular matrix and focal adhesion kinase (FAK) are crucial for the functionality of cancer stem cells, influencing their migration, metastasis formation, and drug resistance. Finally, StellarPath revealed also the significance of two microRNAs: hsa-miR-216a [6] and hsa-miR-216b [7], both associated with pancreatic cancer progression.

[1] https://www.ncbi.nlm.nih.gov/pmc/articles/PMC9735867/

[2] https://www.ncbi.nlm.nih.gov/pmc/articles/PMC3840444/

[3] https://www.ncbi.nlm.nih.gov/pmc/articles/PMC8623975/

[4] https://www.ncbi.nlm.nih.gov/pmc/articles/PMC7604894/

[5] https://www.ncbi.nlm.nih.gov/pmc/articles/PMC4599250/

[6] https://doi.org/10.1016/j.xcrm.2021.100434

[7] https://doi.org/10.3389/fonc.2022.790788

## Section 1.11:

We analysed the PSNs provided by StellarPath and netDx as result of classifying each dataset. We determined the centrality of each patient within these pathway-specific PSNs. For each dataset and method, we constructed a matrix where columns represent patients, rows correspond to pathway-specific PSNs, and each entry indicates the centrality of a patient within a specific pathway. We then subjected each matrix to unsupervised clustering to identify clusters. Specifically, we computed the Euclidean distance between patients, applied the hclust clustering algorithm, and partitioned the resulting hierarchical tree into two primary clusters. Next, we measured the overlap between these clusters and the original patient classes using the Jaccard Index (often referred to as the Jaccard coefficient for clustering). If a method mainly picks PSNs where one class is cohesive and the other is not, then the resulting clusters perfectly mirror the original classes.

## Section 1.12:

We used the Ingenuity Pathway Analysis (IPA) to identify deregulated molecules annotated and associated with Oxygen-Glucose deprivation (OGD). To achieve this, we provided IPA with the deregulated molecules from the pathway-specific PSNs activated by OGD WT compared to N WT, which were also predictive in classifying the samples. Next, we performed an enrichment analysis and built a network associating these deregulated molecules to their annotated phenotypes. We kept only the over-represented phenotypes with a probability value lower than 0.05. This network led the identification of molecules specifically associated with OGD. Exploiting these OGD-annotated molecules, we pinpointed the pathway-specific PSNs that incorporated them. The literature references utilized by IPA to correlate our deregulated molecules with the OGD phenotypes can be found in Table L in S1 Tables and and the network can be found here:

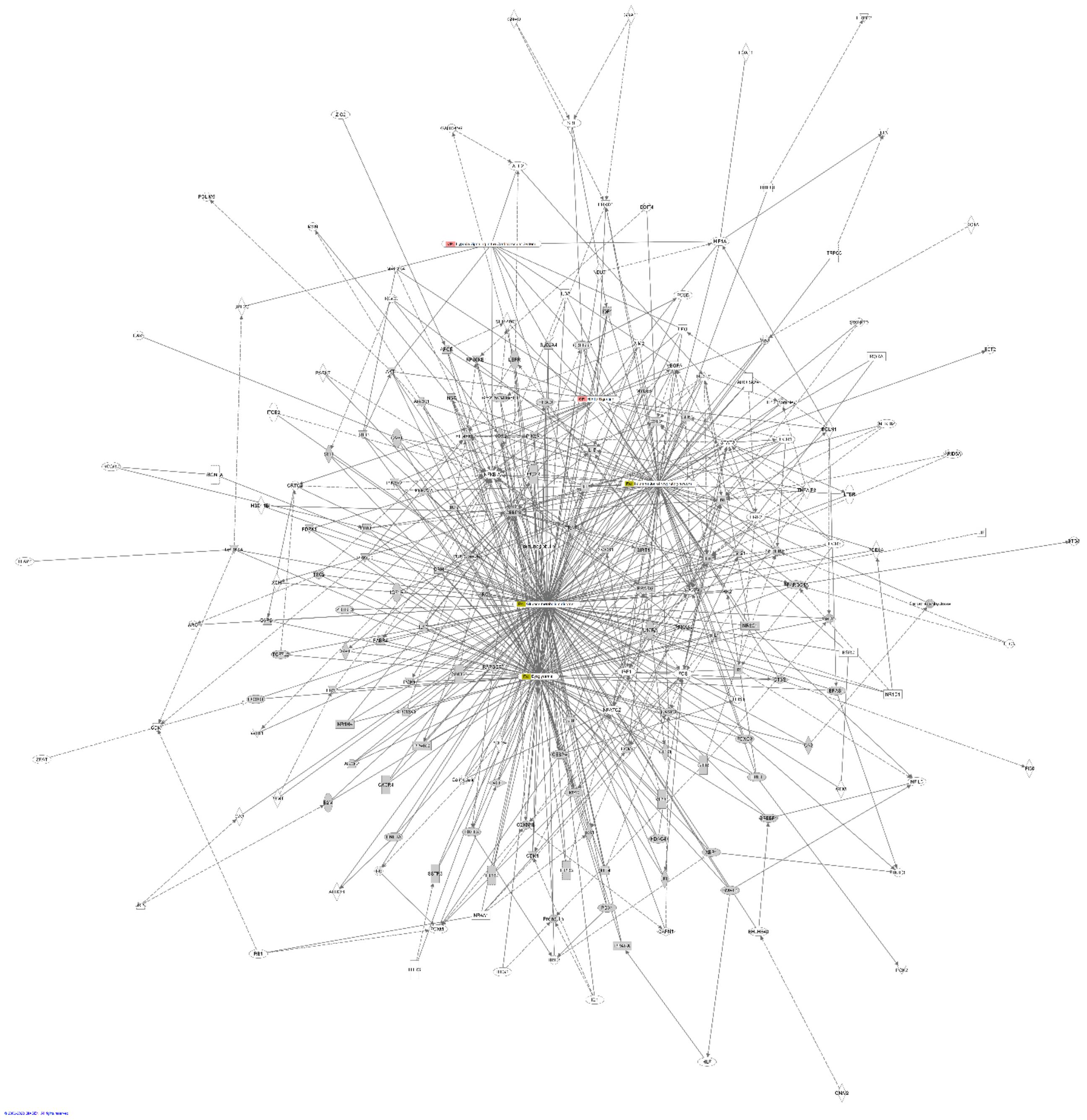

Section 1.13:

It is challenging to make a definitive quantitative and qualitative comparison between StellarPath and all the other pathway analysis tools like GSEA. Firstly because StellarPath solves a problem different from a functional enrichment tool, secondly because each enrichment tool has its unique strengths and weaknesses, and thirdly because the choice of tool often depends on the specific requirements of the analysis and user's preferences.

For example, Gene Set Enrichment Analysis (GSEA) developed by the Broad Institute and StellarPath are both computational methods that identify significant pathways in biological datasets. However, they employ different strategies and have unique features that make them suitable for different types of analyses. Here are the key differences:

1. Input Data: GSEA considers all genes in the dataset, regardless of their expression levels. This approach allows GSEA to detect all changes in gene expression that might be missed when focusing only on differentially expressed genes. On the other hand, StellarPath focuses

only on differentially expressed genes. This approach can be more sensitive to significant changes in gene expression that are relevant to the biological state or condition under study.

2. Statistical Testing: GSEA uses permutation testing to compare the observed enrichment score of a pathway against a null distribution. This method can control for type I errors (false positives) and is robust against variations in gene set sizes. In contrast, StellarPath uses over-representation analysis (ORA) to test whether a pathway is enriched with differentially expressed genes. Additionally, StellarPath checks that the pathway-specific patient similarity network (PSN) representing a gene set is also topologically relevant because it separates the two classes under comparison.

3. Pathway Significance: GSEA is likely to find more significant pathways because a pathway needs to pass only one test (the permutation test). This approach can provide a broad overview of the biological processes involved. However, StellarPath is likely to find fewer significant sets because a pathway has to meet several criteria: it must include differentially expressed genes, pass the ORA test, and produce a predictive PSN that separates the patient classes under study. One test against four.

4. Output Data: GSEA provides the genes, a probability value, an enrichment score associated to each pathway. StellarPath provides the differentially expressed genes, the cohesive patient class, the separability power, the PSN, how much each patient is important for the connectivity of its class, the classification performances and how the pathway is regulated.

At the same time, we understand that comparing StellarPath against GSEA or other functional enrichment tools could attract the interest of many bioinformaticians. Therefore, we compared StellarPath against GSEA in a vignette available on the GitHub page of our software. We summarize the vignette as follows:

We applied StellarPath on our mouse dataset where the classes in comparison are Oxygen-Glucose deprived Wild-Type and Normoxia Wild-Type (mainly because the biological results and differences between the classes are very easy to understand). Then, we applied "GSEA" (Broad Institute algorithm) implemented in the "fgsea" R package available on Bioconductor (We did not use the original software implemented in Java because its usage and results cannot be incorporated in a replicable vignette but the authors of fgsea used Broad GSEA as reference implementation (10.1101/060012v3.full) and found matching results).

GSEA does not work on multi-omics dataset, so we applied GSEA only on the normalized gene expression matrix. We opted to normalize the matrix with Limma workflow, as it was also the one applied by the original author. Next, GSEA, offers two strategies for the gene set enrichment analysis: label-permuting GSEA and preranked GSEA.

- Label-permuting: An Enrichment Score (ES) represents the degree to which a gene set (pathway) is overrepresented at the extremes (top or bottom) of a ranked list of genes. The ES is directly calculated from the original dataset. GSEA uses a permutation procedure that shuffles the patients class labels, preserving the correlation structure among genes, to test whenever the pre-shuffling ES obtained by a pathway is significant against a null distribution of post-shuffling ESs.
    - However, this strategy may be overly conservative when dealing with small sample sizes of the classes.
- Preranked GSEA: a faster version of GSEA that uses a pre-ranked list of genes instead of calculating the ES from the original dataset. The preranked list of genes is generated by ranking all genes based on a gene-level statistic, such as fold change or t-statistic. Unlike label-permuting GSEA, preranked GSEA uses gene label permutation to generate a null distribution of ESs. This procedure permutes the gene labels, which breaks gene-gene correlations.
    - However, the pre-ranked version of GSEA has not been published or endorsed by the original authors of Broad GSEA. The reason is that this strategy can lead to highly

significant pathway even when comparing randomly selected groups of samples, which could lead to misleading results. In fact, this strategy assumes that genes are independent. However, in reality, genes within pathways often exhibit positive correlations. Ignoring these correlations can lead to inflated significance levels. This issue was demonstrated in a study (https://doi.org/10.1093/nar/gks461), which showed that methods assuming gene-wise independence could lead to erroneous results.

We obtained the following results:

- StellarPath found 68 significant pathways
- Label-permuting GSEA did not find any significant pathway
- Preranked GSEA found 481 significant pathways
- The significant pathways found by both StellarPath and PreRank GSEA are 34
- The pathways found only by StellarPath are 34
- The pathways found only by PreRanked GSEA are 447

We then analysed the shared and unique pathways.

The shared pathways found by both methods are:

1. Hypoxia-Related Pathways:
    - HALLMARK_HYPOXIA: Related to low oxygen levels.
    - PID_HIF1_TFPATHWAY: Involves the Hypoxia-inducible factor (HIF) pathway, key in oxygen homeostasis.
2. Inflammatory and Signaling Pathways:
    - HALLMARK_TNFA_SIGNALING_VIA_NFKB: TNF-alpha signaling, associated with inflammation.
    - HALLMARK_IL6_JAK_STAT3_SIGNALING: Involved in immune response and inflammation.
    - HALLMARK_IL2_STAT5_SIGNALING: Part of the immune response.
    - HALLMARK_KRAS_SIGNALING_UP: Related to cell signaling and growth.
3. Metabolic Pathways:
    - HALLMARK_GLYCOLYSIS: Related to energy production, especially under low oxygen.
    - HALLMARK_MTORC1_SIGNALING: Involved in cell growth and metabolism.
4. Cell Death and Apoptosis:
    - HALLMARK_APOPTOSIS: Related to programmed cell death.
    - positive regulation of apoptotic signaling pathway: linked to stress conditions like OGD.
5. Others:
    - Pathways related to cell adhesion, wound healing, sensory perception, and other cellular processes.

The majority of the shared pathways are reasonably associated with Oxygen-Glucose deprivation. Hypoxia-related pathways are directly linked to low oxygen conditions, metabolic pathways are consistent with cellular responses to energy stress, and Inflammation and apoptosis pathways reflect cellular stress and injury responses.

Next, the pathways found only by StellarPath.

StellarPath found unique Inflammatory Pathways activated by/in OGD WT samples:

- HALLMARK_INFLAMMATORY_RESPONSE
- HALLMARK_UV_RESPONSE_UP
- cytokine production
- intrinsic apoptotic signaling pathway
- HALLMARK_INTERFERON_GAMMA_RESPONSE

StellarPath found unique homeostatic pathways activated by/in N WT samples:

- cellular transition metal ion homeostasis
- second-messenger-mediated signaling
- Notch signaling pathway
- HALLMARK_MYOGENESIS
- HALLMARK_ADIPOGENESIS

StellarPath associates precise and relevant pathways to the Oxygen-Glucose deprived samples. These pathways match the expected biological processes affected by cases' condition. At the same time, it also properly recognizes homeostatic cellular functions activated in Normoxia samples.

While GSEA is less focused than StellarPath, it found two categories of pathways:

1. Directly Related to OGD:
    - Pathways like response to hypoxia, cellular response to oxygen levels, and glucose metabolic process are clearly connected to OGD, as they reflect cellular responses to low oxygen and glucose deprivation.
2. Generic or Unrelated to OGD:
    - Several pathways, including those related to muscle development, organ morphogenesis, ion transport, and others, seem too generic or unrelated to OGD. While they might represent broad cellular responses, their direct relevance to OGD is less clear.

In conclusion, GSEA finds much more pathways than StellarPath, some of them are clearly connected to OGD but many of them are generic or seemingly unrelated to the classes in comparison. Plus, GSEA does not provide further information to understand if the pathway is significantly deregulated by one specific class. Instead, StellarPath demonstrates a stronger performance in identifying pathways relevant to Oxygen-Glucose deprivation. Compared to GSEA, StellarPath focuses on pertinent pathways, minimizes the inclusion of unrelated ones, and can recognize that the most generic ones (i.e. homeostatic) are actually regulated by the Normoxia samples and not by the OGD samples which are more oriented to handle the induced stress. StellarPath results are not only scientifically meaningful but also offer a strong base for further wet-lab validations and studies.

We would like to draw attention to a specific detail concerning the preranked strategy in gene set enrichment analysis, a strategy widely used despite known concerns of over-estimation. This strategy has another hidden disadvantage. Let us consider a pathway where half of genes have a

very high positive fold change, while the other half shows a very low negative fold change. Such a pattern would indicate that the pathway is strongly involved and deregulated in the case samples under study. However, tools employing the preranked strategy may fail to recognize this pathway as significant. The reason lies in the calculation of the enrichment score, which, in this scenario, would be close to zero, masking the pathway's true significance. This limitation derives from a lack of annotation in open databases such as GO, KEGG, and Reactome. They do not collect the direction of deregulation of the molecules (i.e., fold change) required to activate or inhibit a pathway. Consequently, it becomes challenging to assess the significance and regulation of a pathway when genes within it are both positively and negatively deregulated. StellarPath does not suffer the same disadvantage because it has to perform two further tests in order to consider a pathway as significant. While enrichment tools do not have other criteria to determine the pathway significance.

REFERENCES:


[1] Law CW, Alhamdoosh M, Su S, Dong X, Tian L, Smyth GK, et al. RNA-seq analysis is easy as 1-2-3 with limma, Glimma and edgeR. F1000Res 2016;5. https://doi.org/10.12688/f1000research.9005.3.

[2] Oughtred R, Rust J, Chang C, Breitkreutz B-J, Stark C, Willems A, et al. The BioGRID database: A comprehensive biomedical resource of curated protein, genetic, and chemical interactions. Protein Sci 2021;30:187–200. https://doi.org/10.1002/pro.3978.

[3] Jensen LJ, Kuhn M, Stark M, Chaffron S, Creevey C, Muller J, et al. STRING 8--a global view on proteins and their functional interactions in 630 organisms. Nucleic Acids Res 2009;37:D412-416. https://doi.org/10.1093/nar/gkn760.

[4] Türei D, Korcsmáros T, Saez-Rodriguez J. OmniPath: guidelines and gateway for literature-curated signaling pathway resources. Nat Methods 2016;13:966–7. https://doi.org/10.1038/nmeth.4077.

[5] Cowen L, Ideker T, Raphael BJ, Sharan R. Network propagation: a universal amplifier of genetic associations. Nat Rev Genet 2017;18:551–62. https://doi.org/10.1038/nrg.2017.38.

[6] Volinia S, Visone R, Galasso M, Rossi E, Croce CM. Identification of microRNA activity by Targets’ Reverse EXpression. Bioinformatics 2010;26:91–7. https://doi.org/10.1093/bioinformatics/btp598.

[7] Baek D, Villén J, Shin C, Camargo FD, Gygi SP, Bartel DP. The impact of microRNAs on protein output. Nature 2008;455:64–71. https://doi.org/10.1038/nature07242.

[8] Chicco D, Jurman G. The advantages of the Matthews correlation coefficient (MCC) over F1 score and accuracy in binary classification evaluation. BMC Genomics 2020;21:6. https://doi.org/10.1186/s12864-019-6413-7.

[9] Chicco D, Tötsch N, Jurman G. The Matthews correlation coefficient (MCC) is more reliable than balanced accuracy, bookmaker informedness, and markedness in two-class confusion matrix evaluation. BioData Mining 2021;14:13. https://doi.org/10.1186/s13040-021-00244-z.


## Section 1.14:

The pathways directly related to hypoxia, such as "response to hypoxia," "cellular response to oxygen levels," and "response to decreased oxygen levels," are expected to be highly relevant in OGD conditions. Similarly, pathways involved in energy metabolism (e.g., "glucose metabolic process"), stress responses, and angiogenesis regulation are also pertinent due to the nature of OGD's impact on cellular energy supply, stress response mechanisms, and the need for restoring blood flow and oxygen supply.

However, GSEA finds also many circumstantial enriched gene sets. They are circumstantial because they could be associated with a stress response, but they are either too much broad or indirect to be firmly associated with OGD. We reviewed the GSEA’s resulting pathways and selected those ones that are less likely to be directly related to OGD based on literature:

- Muscle Organ Development, Skeletal Muscle Cell Differentiation, Skeletal Muscle Organ Development, Skeletal Muscle Tissue Development: These pathways are primarily involved in the development and differentiation of muscle tissues.

- Placenta Development, Embryonic Placenta Development: These pathways are specific to placental development and function, which are important during pregnancy.
- Nuclear Chromosome Segregation, Mitotic Nuclear Division, Mitotic Sister Chromatid Segregation, Sister Chromatid Segregation: These pathways are crucial for cell division and genetic stability, their specific deregulation in OGD is not directly related to the hypoxic or glucose-deprived conditions.
- Regulation of Osteoblast Differentiation, Regulation of Amine Transport, Glycolipid Biosynthetic Process: These pathways are involved in specific physiological processes such as bone formation, neurotransmitter transport, and glycolipid biosynthesis.
- Potassium Ion Transmembrane Transport, Potassium Ion Transport: ion transport is crucial for maintaining cellular homeostasis
- Chromosome Localization, Regulation of Chromosome Organization, Establishment of Chromosome Localization: These pathways are involved in the spatial arrangement and structural organization of chromosomes.
- Regulation of Neurotransmitter Secretion, Regulation of Neurotransmitter Transport: neurotransmitter mechanisms are crucial in neuronal communication but are not necessarily affected by OGD condition.
- Positive Regulation of Osteoblast Differentiation: This pathway is specifically involved in bone formation and differentiation.
- Wound Healing, Stem Cell Population Maintenance: These are broad physiological processes that, while potentially affected by OGD indirectly through tissue damage and repair mechanisms, are not directly associated with the cellular response to hypoxia and energy depletion typical of OGD.
- Artery Development, Regulation of Blood Vessel Endothelial Cell Migration: These pathways are involved in vascular development and repair.
- Morphogenesis of a Branching Epithelium: This pathway is involved in the development of structures in the epithelial tissues.
- Circadian Regulation of Gene Expression: Circadian rhythms regulate various physiological processes, including gene expression. However, it is difficult to associate them to OGD.
- Cardiac Septum Morphogenesis, Cardiac Septum Development, Cardiac Chamber Development: These pathways are involved in the development of heart structures.
- Vesicle-Mediated Transport in Synapse: This pathway is specific to synaptic function and neurotransmitter release.
- Regulation of Catecholamine and Dopamine Secretion: These pathways are involved in the secretion of specific neurotransmitters.
- Response to Muramyl Dipeptide: This pathway is involved in the immune response to bacterial peptidoglycan.
- Ganglioside Biosynthetic Process: This pathway is involved in the synthesis of gangliosides, which are important for cell membrane composition and function, particularly in the nervous system.
- Labyrinthine Layer Development, Lung Morphogenesis, Lung Vasculature Development: These pathways are involved in the development of specific anatomical structures.

- Modulation by Host of Symbiont Process, Biological Process Involved in Interaction with Symbiont: These pathways involve host-symbiont interactions which are absent in the OGD experiment.
- Behavioral Fear Response: This pathway involves the organismal level response to fear and is more related to the neurological and psychological responses rather than cellular metabolic stress or hypoxic conditions induced by OGD.
