# Supplementary material for: StellarPath: Hierarchical-vertical multi-omics classifier synergizes stable markers and interpretable similarity networks for patient profiling
Source: PLoS Comput Biol. 2024 Apr 12;20(4):e1012022. doi: 10.1371/journal.pcbi.1012022 (PMC11042724; doi:10.1371/journal.pcbi.1012022)

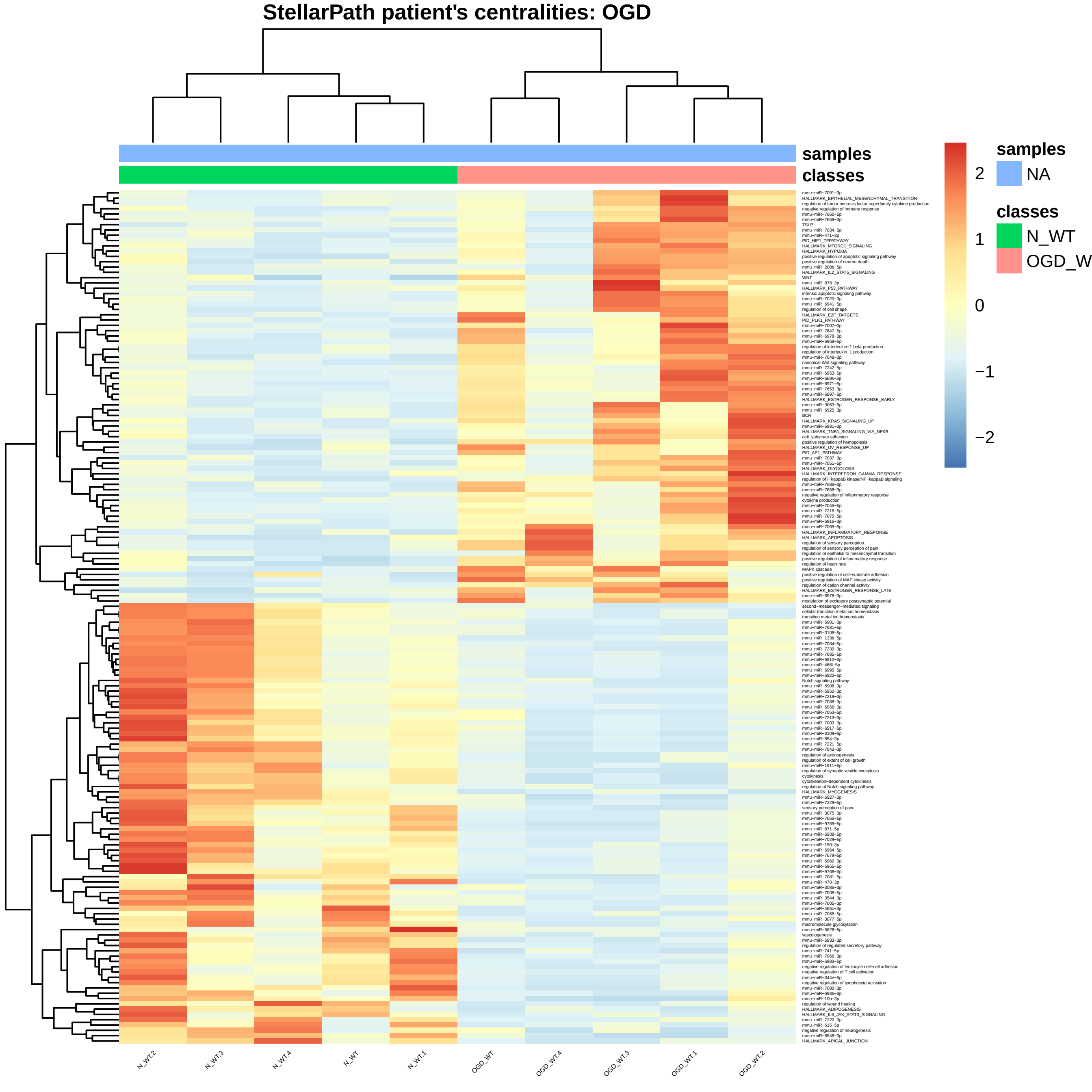

StellarPath patient's centralities: OGD
samples
classes
samples
NA
classes
N_WT
OGD_W
2
1
0
−1
−2
mmu−miR−7091−3p
HALLMARK_EPITHELIAL_MESENCHYMAL_TRANSITION
regulation of tumor necrosis factor superfamily cytokine production
negative regulation of immune response
mmu−miR−7660−5p
mmu−miR−7659−3p
TSLP
mmu−miR−7034−5p
mmu−miR−471−3p
PID_HIF1_TFPATHWAY
HALLMARK_MTORC1_SIGNALING
HALLMARK_HYPOXIA
positive regulation of apoptotic signaling pathway
positive regulation of neuron death
mmu−miR−208b−5p
HALLMARK_IL2_STAT5_SIGNALING
WNT
mmu−miR−878−3p
HALLMARK_P53_PATHWAY
intrinsic apoptotic signaling pathway
mmu−miR−7020−3p
mmu−miR−6941−5p
regulation of cell shape
HALLMARK_E2F_TARGETS
PID_PLK1_PATHWAY
mmu−miR−7007−3p
mmu−miR−7647−5p
mmu−miR−6978−3p
mmu−miR−6968−5p
regulation of interleukin−1 beta production
regulation of interleukin−1 production
mmu−miR−7049−3p
canonical Wnt signaling pathway
mmu−miR−7242−5p
mmu−miR−6953−5p
mmu−miR−669k−3p
mmu−miR−6971−5p
mmu−miR−7653−3p
mmu−miR−6897−5p
HALLMARK_ESTROGEN_RESPONSE_EARLY
mmu−miR−3063−5p
mmu−miR−6925−3p
BCR
HALLMARK_KRAS_SIGNALING_UP
mmu−miR−6981−3p
HALLMARK_TNFA_SIGNALING_VIA_NFKB
cell−substrate adhesion
positive regulation of hemopoiesis
HALLMARK_UV_RESPONSE_UP
PID_AP1_PATHWAY
mmu−miR−7037−3p
mmu−miR−7051−5p
HALLMARK_GLYCOLYSIS
HALLMARK_INTERFERON_GAMMA_RESPONSE
regulation of I−kappaB kinase/NF−kappaB signaling
mmu−miR−7686−3p
mmu−miR−7658−3p
negative regulation of inflammatory response
cytokine production
mmu−miR−7045−5p
mmu−miR−7218−5p
mmu−miR−7075−5p
mmu−miR−6916−3p
mmu−miR−7065−5p
HALLMARK_INFLAMMATORY_RESPONSE
HALLMARK_APOPTOSIS
regulation of sensory perception
regulation of sensory perception of pain
regulation of epithelial to mesenchymal transition
positive regulation of inflammatory response
regulation of heart rate
MAPK cascade
positive regulation of cell−substrate adhesion
positive regulation of MAP kinase activity
regulation of cation channel activity
HALLMARK_ESTROGEN_RESPONSE_LATE
mmu−miR−6976−3p
modulation of excitatory postsynaptic potential
second−messenger−mediated signaling
cellular transition metal ion homeostasis
transition metal ion homeostasis
mmu−miR−6901−3p
mmu−miR−7681−5p
mmu−miR−3108−5p
mmu−miR−133b−5p
mmu−miR−7084−5p
mmu−miR−7230−3p
mmu−miR−7685−5p
mmu−miR−6910−3p
mmu−miR−466l−5p
mmu−miR−6995−5p
mmu−miR−6923−5p
Notch signaling pathway
mmu−miR−6908−3p
mmu−miR−6950−3p
mmu−miR−7219−3p
mmu−miR−7088−3p
mmu−miR−6956−3p
mmu−miR−7053−5p
mmu−miR−7213−3p
mmu−miR−7003−3p
mmu−miR−6917−5p
mmu−miR−3109−5p
mmu−miR−654−3p
mmu−miR−7221−5p
mmu−miR−7042−3p
regulation of axonogenesis
regulation of extent of cell growth
mmu−miR−1911−5p
regulation of synaptic vesicle exocytosis
cytokinesis
cytoskeleton−dependent cytokinesis
regulation of Notch signaling pathway
HALLMARK_MYOGENESIS
mmu−miR−6927−3p
mmu−miR−7229−5p
sensory perception of pain
mmu−miR−3075−3p
mmu−miR−7666−5p
mmu−miR−9769−5p
mmu−miR−871−5p
mmu−miR−6939−5p
mmu−miR−7029−5p
mmu−miR−150−3p
mmu−miR−6964−5p
mmu−miR−7679−5p
mmu−miR−6960−3p
mmu−miR−6965−5p
mmu−miR−9768−3p
mmu−miR−7081−5p
mmu−miR−470−3p
mmu−miR−3086−3p
mmu−miR−7008−5p
mmu−miR−3544−3p
mmu−miR−7005−3p
mmu−miR−465c−3p
mmu−miR−7068−5p
mmu−miR−3077−5p
macromolecule glycosylation
mmu−miR−5626−5p
vasculogenesis
mmu−miR−6933−3p
regulation of regulated secretory pathway
mmu−miR−741−5p
mmu−miR−7066−3p
mmu−miR−6983−5p
negative regulation of leukocyte cell−cell adhesion
negative regulation of T cell activation
mmu−miR−344e−5p
negative regulation of lymphocyte activation
mmu−miR−7080−3p
mmu−miR−6936−3p
mmu−miR−10b−3p
regulation of wound healing
HALLMARK_ADIPOGENESIS
HALLMARK_IL6_JAK_STAT3_SIGNALING
mmu−miR−7220−3p
mmu−miR−615−5p
negative regulation of neurogenesis
mmu−miR−6546−3p
HALLMARK_APICAL_JUNCTION
N_WT.2
N_WT.3
N_WT.4
N_WT
N_WT.1
OGD_WT
OGD_WT.4
OGD_WT.3
OGD_WT.1
OGD_WT.2

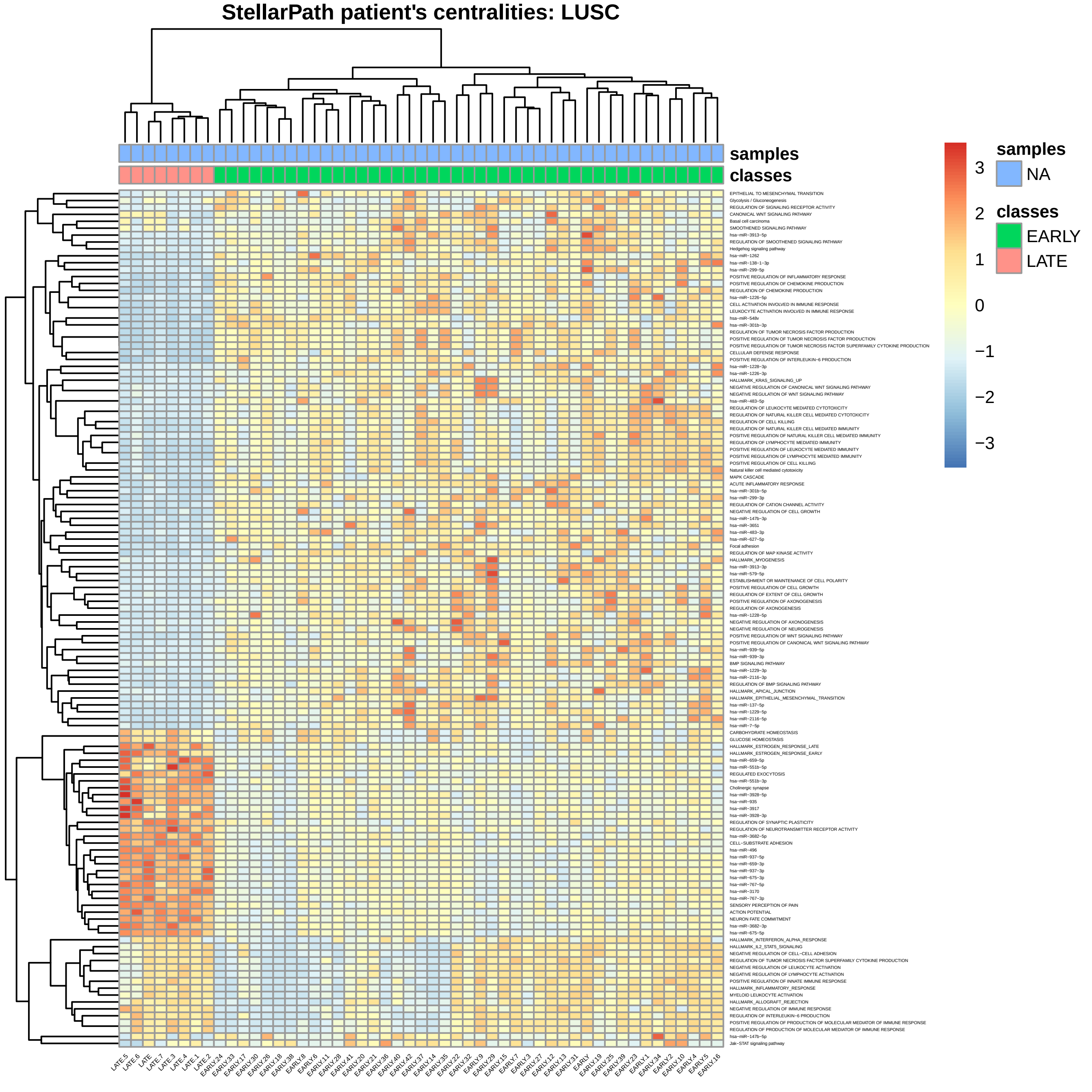
StellarPath patient's centralities: LUSC
samples
classes
samples
NA
classes
EARLY
LATE
3
2
1
0
−1
−2
−3
EPITHELIAL TO MESENCHYMAL TRANSITION
Glycolysis / Gluconeogenesis
REGULATION OF SIGNALING RECEPTOR ACTIVITY
CANONICAL WNT SIGNALING PATHWAY
Basal cell carcinoma
SMOOTHENED SIGNALING PATHWAY
hsa−miR−3913−5p
REGULATION OF SMOOTHENED SIGNALING PATHWAY
Hedgehog signaling pathway
hsa−miR−1262
hsa−miR−138−1−3p
hsa−miR−299−5p
POSITIVE REGULATION OF INFLAMMATORY RESPONSE
POSITIVE REGULATION OF CHEMOKINE PRODUCTION
REGULATION OF CHEMOKINE PRODUCTION
hsa−miR−1226−5p
CELL ACTIVATION INVOLVED IN IMMUNE RESPONSE
LEUKOCYTE ACTIVATION INVOLVED IN IMMUNE RESPONSE
hsa−miR−548v
hsa−miR−301b−3p
REGULATION OF TUMOR NECROSIS FACTOR PRODUCTION
POSITIVE REGULATION OF TUMOR NECROSIS FACTOR PRODUCTION
POSITIVE REGULATION OF TUMOR NECROSIS FACTOR SUPERFAMILY CYTOKINE PRODUCTION
CELLULAR DEFENSE RESPONSE
POSITIVE REGULATION OF INTERLEUKIN−6 PRODUCTION
hsa−miR−1228−3p
hsa−miR−1226−3p
HALLMARK_KRAS_SIGNALING_UP
NEGATIVE REGULATION OF CANONICAL WNT SIGNALING PATHWAY
NEGATIVE REGULATION OF WNT SIGNALING PATHWAY
hsa−miR−483−5p
REGULATION OF LEUKOCYTE MEDIATED CYTOTOXICITY
REGULATION OF NATURAL KILLER CELL MEDIATED CYTOTOXICITY
REGULATION OF CELL KILLING
REGULATION OF NATURAL KILLER CELL MEDIATED IMMUNITY
POSITIVE REGULATION OF NATURAL KILLER CELL MEDIATED IMMUNITY
REGULATION OF LYMPHOCYTE MEDIATED IMMUNITY
POSITIVE REGULATION OF LEUKOCYTE MEDIATED IMMUNITY
POSITIVE REGULATION OF LYMPHOCYTE MEDIATED IMMUNITY
POSITIVE REGULATION OF CELL KILLING
Natural killer cell mediated cytotoxicity
MAPK CASCADE
ACUTE INFLAMMATORY RESPONSE
hsa−miR−301b−5p
hsa−miR−299−3p
REGULATION OF CATION CHANNEL ACTIVITY
NEGATIVE REGULATION OF CELL GROWTH
hsa−miR−147b−3p
hsa−miR−3651
hsa−miR−483−3p
hsa−miR−627−5p
Focal adhesion
REGULATION OF MAP KINASE ACTIVITY
HALLMARK_MYOGENESIS
hsa−miR−3913−3p
hsa−miR−579−5p
ESTABLISHMENT OR MAINTENANCE OF CELL POLARITY
POSITIVE REGULATION OF CELL GROWTH
REGULATION OF EXTENT OF CELL GROWTH
POSITIVE REGULATION OF AXONOGENESIS
REGULATION OF AXONOGENESIS
hsa−miR−1228−5p
NEGATIVE REGULATION OF AXONOGENESIS
NEGATIVE REGULATION OF NEUROGENESIS
POSITIVE REGULATION OF WNT SIGNALING PATHWAY
POSITIVE REGULATION OF CANONICAL WNT SIGNALING PATHWAY
hsa−miR−939−5p
hsa−miR−939−3p
BMP SIGNALING PATHWAY
hsa−miR−1229−3p
hsa−miR−2116−3p
REGULATION OF BMP SIGNALING PATHWAY
HALLMARK_APICAL_JUNCTION
HALLMARK_EPITHELIAL_MESENCHYMAL_TRANSITION
hsa−miR−137−5p
hsa−miR−1229−5p
hsa−miR−2116−5p
hsa−miR−7−5p
CARBOHYDRATE HOMEOSTASIS
GLUCOSE HOMEOSTASIS
HALLMARK_ESTROGEN_RESPONSE_LATE
HALLMARK_ESTROGEN_RESPONSE_EARLY
hsa−miR−659−5p
hsa−miR−551b−5p
REGULATED EXOCYTOSIS
hsa−miR−551b−3p
Cholinergic synapse
hsa−miR−3928−5p
hsa−miR−935
hsa−miR−3917
hsa−miR−3928−3p
REGULATION OF SYNAPTIC PLASTICITY
REGULATION OF NEUROTRANSMITTER RECEPTOR ACTIVITY
hsa−miR−3682−5p
CELL−SUBSTRATE ADHESION
hsa−miR−496
hsa−miR−937−5p
hsa−miR−659−3p
hsa−miR−937−3p
hsa−miR−675−3p
hsa−miR−767−5p
hsa−miR−3170
hsa−miR−767−3p
SENSORY PERCEPTION OF PAIN
ACTION POTENTIAL
NEURON FATE COMMITMENT
hsa−miR−3682−3p
hsa−miR−675−5p
HALLMARK_INTERFERON_ALPHA_RESPONSE
HALLMARK_IL2_STAT5_SIGNALING
NEGATIVE REGULATION OF CELL−CELL ADHESION
REGULATION OF TUMOR NECROSIS FACTOR SUPERFAMILY CYTOKINE PRODUCTION
NEGATIVE REGULATION OF LEUKOCYTE ACTIVATION
NEGATIVE REGULATION OF LYMPHOCYTE ACTIVATION
POSITIVE REGULATION OF INNATE IMMUNE RESPONSE
HALLMARK_INFLAMMATORY_RESPONSE
MYELOID LEUKOCYTE ACTIVATION
HALLMARK_ALLOGRAFT_REJECTION
NEGATIVE REGULATION OF IMMUNE RESPONSE
REGULATION OF INTERLEUKIN−6 PRODUCTION
POSITIVE REGULATION OF PRODUCTION OF MOLECULAR MEDIATOR OF IMMUNE RESPONSE
REGULATION OF PRODUCTION OF MOLECULAR MEDIATOR OF IMMUNE RESPONSE
hsa−miR−147b−5p
Jak−STAT signaling pathway
LATE.5
LATE.6
LATE
LATE.7
LATE.3
LATE.4
LATE.1
LATE.2
EARLY.24
EARLY.33
EARLY.17
EARLY.30
EARLY.26
EARLY.18
EARLY.38
EARLY.8
EARLY6
EARLY.11
EARLY.28
EARLY.41
EARLY.20
EARLY.21
EARLY.36
EARLY.40
EARLY.42
EARLY.37
EARLY.14
EARLY.35
EARLY.22
EARLY.32
EARLY.9
EARLY.29
EARLY.15
EARLY.7
EARLY.3
EARLY.27
EARLY.12
EARLY.13
EARLY.31
EARLY
EARLY.19
EARLY.25
EARLY.39
EARLY.23
EARLY.1
EARLY.34
EARLY.2
EARLY.10
EARLY.4
EARLY.5
EARLY.16

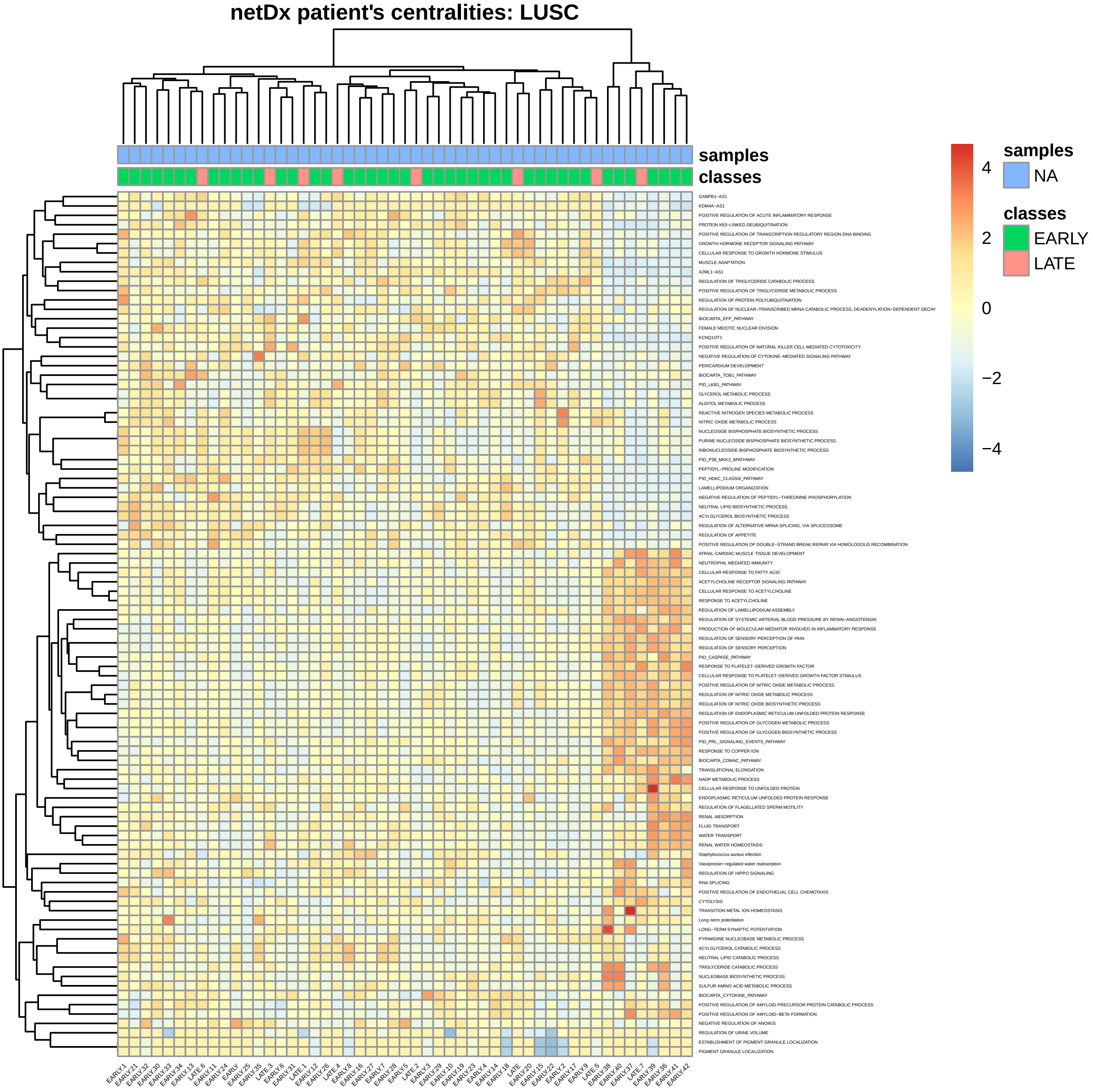
netDx patient's centralities: LUSC
samples
classes
samples
NA
classes
EARLY
LATE
4
2
0
−2
−4
GABPB1−AS1
KDM4A−AS1
POSITIVE REGULATION OF ACUTE INFLAMMATORY RESPONSE
PROTEIN K63−LINKED DEUBIQUITINATION
POSITIVE REGULATION OF TRANSCRIPTION REGULATORY REGION DNA BINDING
GROWTH HORMONE RECEPTOR SIGNALING PATHWAY
CELLULAR RESPONSE TO GROWTH HORMONE STIMULUS
MUSCLE ADAPTATION
A2ML1−AS1
REGULATION OF TRIGLYCERIDE CATABOLIC PROCESS
POSITIVE REGULATION OF TRIGLYCERIDE METABOLIC PROCESS
REGULATION OF PROTEIN POLYUBIQUITINATION
REGULATION OF NUCLEAR−TRANSCRIBED MRNA CATABOLIC PROCESS, DEADENYLATION−DEPENDENT DECAY
BIOCARTA_EFP_PATHWAY
FEMALE MEIOTIC NUCLEAR DIVISION
KCNQ1OT1
POSITIVE REGULATION OF NATURAL KILLER CELL MEDIATED CYTOTOXICITY
NEGATIVE REGULATION OF CYTOKINE−MEDIATED SIGNALING PATHWAY
PERICARDIUM DEVELOPMENT
BIOCARTA_TOB1_PATHWAY
PID_LKB1_PATHWAY
GLYCEROL METABOLIC PROCESS
ALDITOL METABOLIC PROCESS
REACTIVE NITROGEN SPECIES METABOLIC PROCESS
NITRIC OXIDE METABOLIC PROCESS
NUCLEOSIDE BISPHOSPHATE BIOSYNTHETIC PROCESS
PURINE NUCLEOSIDE BISPHOSPHATE BIOSYNTHETIC PROCESS
RIBONUCLEOSIDE BISPHOSPHATE BIOSYNTHETIC PROCESS
PID_P38_MKK3_6PATHWAY
PEPTIDYL−PROLINE MODIFICATION
PID_HDAC_CLASSIII_PATHWAY
LAMELLIPODIUM ORGANIZATION
NEGATIVE REGULATION OF PEPTIDYL−THREONINE PHOSPHORYLATION
NEUTRAL LIPID BIOSYNTHETIC PROCESS
ACYLGLYCEROL BIOSYNTHETIC PROCESS
REGULATION OF ALTERNATIVE MRNA SPLICING, VIA SPLICEOSOME
REGULATION OF APPETITE
POSITIVE REGULATION OF DOUBLE−STRAND BREAK REPAIR VIA HOMOLOGOUS RECOMBINATION
ATRIAL CARDIAC MUSCLE TISSUE DEVELOPMENT
NEUTROPHIL MEDIATED IMMUNITY
CELLULAR RESPONSE TO FATTY ACID
ACETYLCHOLINE RECEPTOR SIGNALING PATHWAY
CELLULAR RESPONSE TO ACETYLCHOLINE
RESPONSE TO ACETYLCHOLINE
REGULATION OF LAMELLIPODIUM ASSEMBLY
REGULATION OF SYSTEMIC ARTERIAL BLOOD PRESSURE BY RENIN−ANGIOTENSIN
PRODUCTION OF MOLECULAR MEDIATOR INVOLVED IN INFLAMMATORY RESPONSE
REGULATION OF SENSORY PERCEPTION OF PAIN
REGULATION OF SENSORY PERCEPTION
PID_CASPASE_PATHWAY
RESPONSE TO PLATELET−DERIVED GROWTH FACTOR
CELLULAR RESPONSE TO PLATELET−DERIVED GROWTH FACTOR STIMULUS
POSITIVE REGULATION OF NITRIC OXIDE METABOLIC PROCESS
REGULATION OF NITRIC OXIDE METABOLIC PROCESS
REGULATION OF NITRIC OXIDE BIOSYNTHETIC PROCESS
REGULATION OF ENDOPLASMIC RETICULUM UNFOLDED PROTEIN RESPONSE
POSITIVE REGULATION OF GLYCOGEN METABOLIC PROCESS
POSITIVE REGULATION OF GLYCOGEN BIOSYNTHETIC PROCESS
PID_PRL_SIGNALING_EVENTS_PATHWAY
RESPONSE TO COPPER ION
BIOCARTA_CDMAC_PATHWAY
TRANSLATIONAL ELONGATION
NADP METABOLIC PROCESS
CELLULAR RESPONSE TO UNFOLDED PROTEIN
ENDOPLASMIC RETICULUM UNFOLDED PROTEIN RESPONSE
REGULATION OF FLAGELLATED SPERM MOTILITY
RENAL ABSORPTION
FLUID TRANSPORT
WATER TRANSPORT
RENAL WATER HOMEOSTASIS
Staphylococcus aureus infection
Vasopressin−regulated water reabsorption
REGULATION OF HIPPO SIGNALING
RNA SPLICING
POSITIVE REGULATION OF ENDOTHELIAL CELL CHEMOTAXIS
CYTOLYSIS
TRANSITION METAL ION HOMEOSTASIS
Long−term potentiation
LONG−TERM SYNAPTIC POTENTIATION
PYRIMIDINE NUCLEOBASE METABOLIC PROCESS
ACYLGLYCEROL CATABOLIC PROCESS
NEUTRAL LIPID CATABOLIC PROCESS
TRIGLYCERIDE CATABOLIC PROCESS
NUCLEOBASE BIOSYNTHETIC PROCESS
SULFUR AMINO ACID METABOLIC PROCESS
BIOCARTA_CYTOKINE_PATHWAY
POSITIVE REGULATION OF AMYLOID PRECURSOR PROTEIN CATABOLIC PROCESS
POSITIVE REGULATION OF AMYLOID−BETA FORMATION
NEGATIVE REGULATION OF ANOIKIS
REGULATION OF URINE VOLUME
ESTABLISHMENT OF PIGMENT GRANULE LOCALIZATION
PIGMENT GRANULE LOCALIZATION
EARLY.1
EARLY.21
EARLY.32
EARLY.30
EARLY.33
EARLY.34
EARLY.13
LATE.6
EARLY.11
EARLY.24
EARLY
EARLY.25
EARLY.35
LATE.3
EARLY.6
EARLY.31
LATE.1
EARLY.12
EARLY.26
LATE.4
EARLY.8
EARLY.16
EARLY.27
EARLY.7
EARLY.28
EARLY.5
LATE.2
EARLY.3
EARLY.29
EARLY.10
EARLY.19
EARLY.23
EARLY.4
EARLY.14
EARLY.18
LATE
EARLY.20
EARLY.15
EARLY.22
EARLY.2
EARLY.17
EARLY.9
LATE.5
EARLY.38
EARLY.40
EARLY.37
LATE.7
EARLY.39
EARLY.36
EARLY.41
EARLY.42

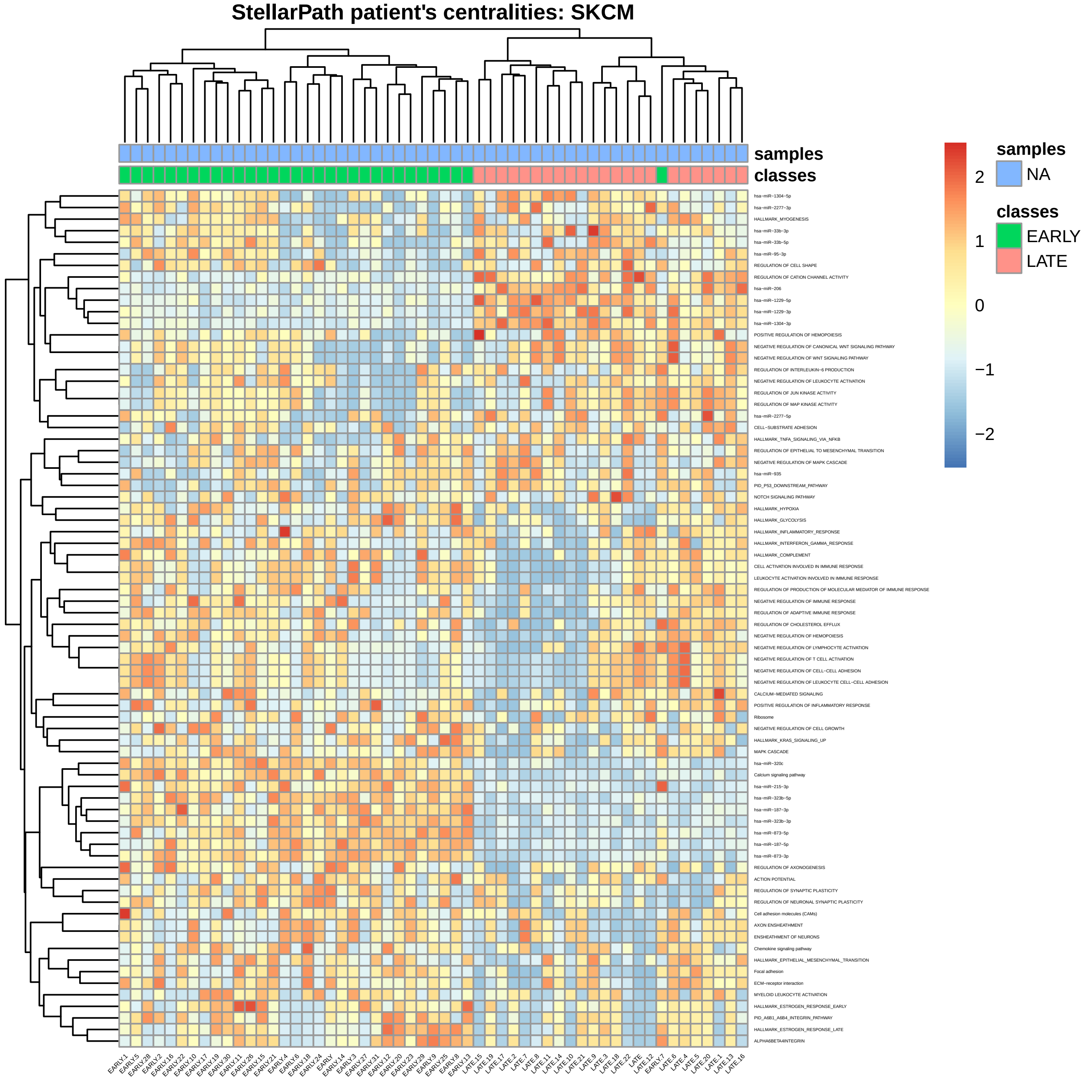
StellarPath patient's centralities: SKCM
samples
classes
samples
NA
classes
EARLY
LATE
2
1
0
−1
−2
hsa−miR−1304−5p
hsa−miR−2277−3p
HALLMARK_MYOGENESIS
hsa−miR−33b−3p
hsa−miR−33b−5p
hsa−miR−95−3p
REGULATION OF CELL SHAPE
REGULATION OF CATION CHANNEL ACTIVITY
hsa−miR−206
hsa−miR−1229−5p
hsa−miR−1229−3p
hsa−miR−1304−3p
POSITIVE REGULATION OF HEMOPOIESIS
NEGATIVE REGULATION OF CANONICAL WNT SIGNALING PATHWAY
NEGATIVE REGULATION OF WNT SIGNALING PATHWAY
REGULATION OF INTERLEUKIN−6 PRODUCTION
NEGATIVE REGULATION OF LEUKOCYTE ACTIVATION
REGULATION OF JUN KINASE ACTIVITY
REGULATION OF MAP KINASE ACTIVITY
hsa−miR−2277−5p
CELL−SUBSTRATE ADHESION
HALLMARK_TNFA_SIGNALING_VIA_NFKB
REGULATION OF EPITHELIAL TO MESENCHYMAL TRANSITION
NEGATIVE REGULATION OF MAPK CASCADE
hsa−miR−935
PID_P53_DOWNSTREAM_PATHWAY
NOTCH SIGNALING PATHWAY
HALLMARK_HYPOXIA
HALLMARK_GLYCOLYSIS
HALLMARK_INFLAMMATORY_RESPONSE
HALLMARK_INTERFERON_GAMMA_RESPONSE
HALLMARK_COMPLEMENT
CELL ACTIVATION INVOLVED IN IMMUNE RESPONSE
LEUKOCYTE ACTIVATION INVOLVED IN IMMUNE RESPONSE
REGULATION OF PRODUCTION OF MOLECULAR MEDIATOR OF IMMUNE RESPONSE
NEGATIVE REGULATION OF IMMUNE RESPONSE
REGULATION OF ADAPTIVE IMMUNE RESPONSE
REGULATION OF CHOLESTEROL EFFLUX
NEGATIVE REGULATION OF HEMOPOIESIS
NEGATIVE REGULATION OF LYMPHOCYTE ACTIVATION
NEGATIVE REGULATION OF T CELL ACTIVATION
NEGATIVE REGULATION OF CELL−CELL ADHESION
NEGATIVE REGULATION OF LEUKOCYTE CELL−CELL ADHESION
CALCIUM−MEDIATED SIGNALING
POSITIVE REGULATION OF INFLAMMATORY RESPONSE
Ribosome
NEGATIVE REGULATION OF CELL GROWTH
HALLMARK_KRAS_SIGNALING_UP
MAPK CASCADE
hsa−miR−320c
Calcium signaling pathway
hsa−miR−215−3p
hsa−miR−323b−5p
hsa−miR−187−3p
hsa−miR−323b−3p
hsa−miR−873−5p
hsa−miR−187−5p
hsa−miR−873−3p
REGULATION OF AXONOGENESIS
ACTION POTENTIAL
REGULATION OF SYNAPTIC PLASTICITY
REGULATION OF NEURONAL SYNAPTIC PLASTICITY
Cell adhesion molecules (CAMs)
AXON ENSHEATHMENT
ENSHEATHMENT OF NEURONS
Chemokine signaling pathway
HALLMARK_EPITHELIAL_MESENCHYMAL_TRANSITION
Focal adhesion
ECM−receptor interaction
MYELOID LEUKOCYTE ACTIVATION
HALLMARK_ESTROGEN_RESPONSE_EARLY
PID_A6B1_A6B4_INTEGRIN_PATHWAY
HALLMARK_ESTROGEN_RESPONSE_LATE
ALPHA6BETA4INTEGRIN
EARLY.1
EARLY.5
EARLY.28
EARLY.2
EARLY.16
EARLY.22
EARLY.10
EARLY.17
EARLY.19
EARLY.30
EARLY.11
EARLY.26
EARLY.15
EARLY.21
EARLY.4
EARLY.6
EARLY.18
EARLY.24
EARLY
EARLY.14
EARLY.3
EARLY.27
EARLY.31
EARLY.12
EARLY.20
EARLY.23
EARLY.29
EARLY.9
EARLY.25
EARLY.8
EARLY.13
LATE.15
LATE.19
LATE.17
LATE.2
LATE.7
LATE.8
LATE.11
LATE.14
LATE.10
LATE.21
LATE.9
LATE.3
LATE.18
LATE.22
LATE
LATE.12
EARLY.7
LATE.6
LATE.4
LATE.5
LATE.20
LATE.1
LATE.13
LATE.16

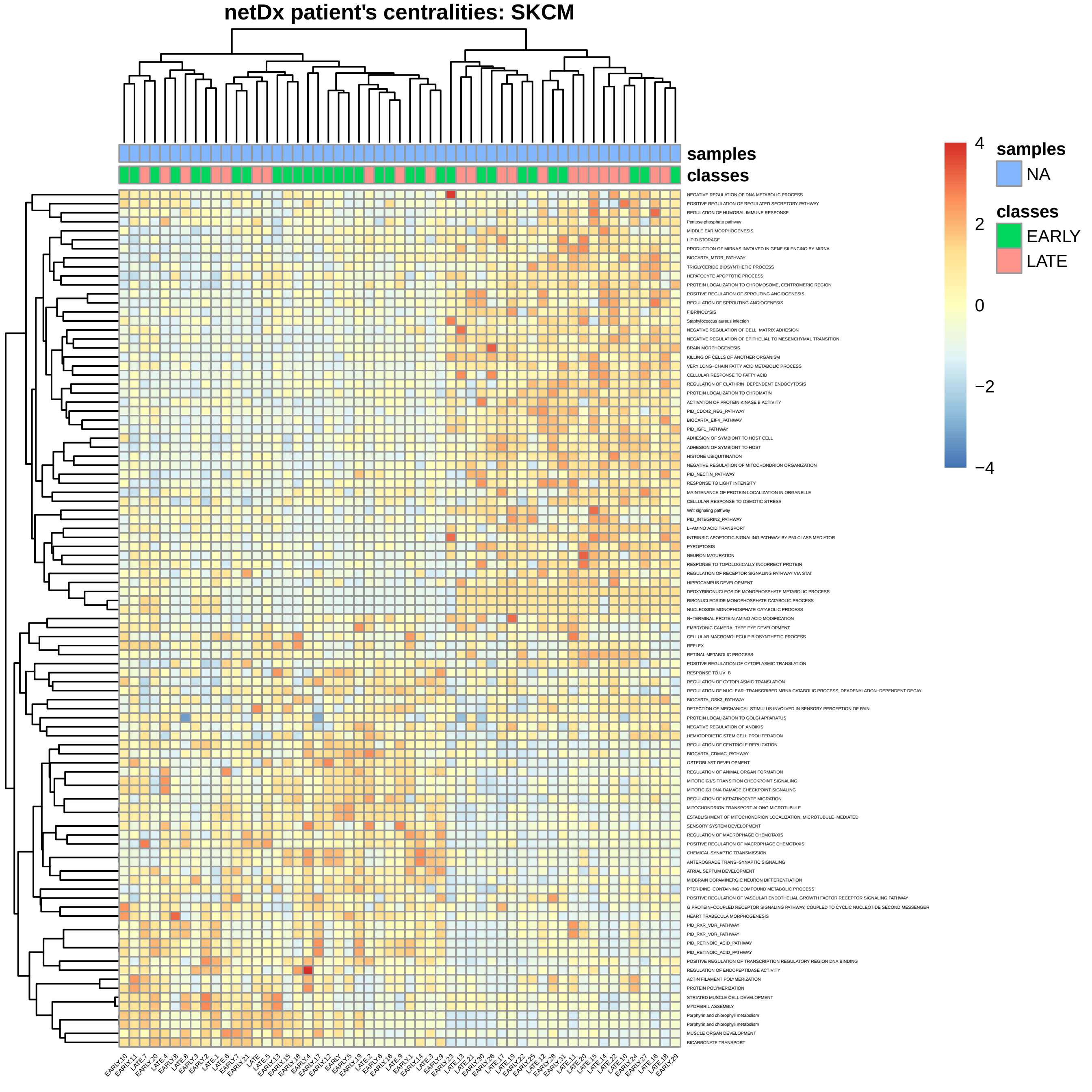
netDx patient's centralities: SKCM
samples
classes
samples
NA
classes
EARLY
LATE
4
2
0
−2
−4
NEGATIVE REGULATION OF DNA METABOLIC PROCESS
POSITIVE REGULATION OF REGULATED SECRETORY PATHWAY
REGULATION OF HUMORAL IMMUNE RESPONSE
Pentose phosphate pathway
MIDDLE EAR MORPHOGENESIS
LIPID STORAGE
PRODUCTION OF MIRNAS INVOLVED IN GENE SILENCING BY MIRNA
BIOCARTA_MTOR_PATHWAY
TRIGLYCERIDE BIOSYNTHETIC PROCESS
HEPATOCYTE APOPTOTIC PROCESS
PROTEIN LOCALIZATION TO CHROMOSOME, CENTROMERIC REGION
POSITIVE REGULATION OF SPROUTING ANGIOGENESIS
REGULATION OF SPROUTING ANGIOGENESIS
FIBRINOLYSIS
Staphylococcus aureus infection
NEGATIVE REGULATION OF CELL−MATRIX ADHESION
NEGATIVE REGULATION OF EPITHELIAL TO MESENCHYMAL TRANSITION
BRAIN MORPHOGENESIS
KILLING OF CELLS OF ANOTHER ORGANISM
VERY LONG−CHAIN FATTY ACID METABOLIC PROCESS
CELLULAR RESPONSE TO FATTY ACID
REGULATION OF CLATHRIN−DEPENDENT ENDOCYTOSIS
PROTEIN LOCALIZATION TO CHROMATIN
ACTIVATION OF PROTEIN KINASE B ACTIVITY
PID_CDC42_REG_PATHWAY
BIOCARTA_EIF4_PATHWAY
PID_IGF1_PATHWAY
ADHESION OF SYMBIONT TO HOST CELL
ADHESION OF SYMBIONT TO HOST
HISTONE UBIQUITINATION
NEGATIVE REGULATION OF MITOCHONDRION ORGANIZATION
PID_NECTIN_PATHWAY
RESPONSE TO LIGHT INTENSITY
MAINTENANCE OF PROTEIN LOCALIZATION IN ORGANELLE
CELLULAR RESPONSE TO OSMOTIC STRESS
Wnt signaling pathway
PID_INTEGRIN2_PATHWAY
L−AMINO ACID TRANSPORT
INTRINSIC APOPTOTIC SIGNALING PATHWAY BY P53 CLASS MEDIATOR
PYROPTOSIS
NEURON MATURATION
RESPONSE TO TOPOLOGICALLY INCORRECT PROTEIN
REGULATION OF RECEPTOR SIGNALING PATHWAY VIA STAT
HIPPOCAMPUS DEVELOPMENT
DEOXYRIBONUCLEOSIDE MONOPHOSPHATE METABOLIC PROCESS
RIBONUCLEOSIDE MONOPHOSPHATE CATABOLIC PROCESS
NUCLEOSIDE MONOPHOSPHATE CATABOLIC PROCESS
N−TERMINAL PROTEIN AMINO ACID MODIFICATION
EMBRYONIC CAMERA−TYPE EYE DEVELOPMENT
CELLULAR MACROMOLECULE BIOSYNTHETIC PROCESS
REFLEX
RETINAL METABOLIC PROCESS
POSITIVE REGULATION OF CYTOPLASMIC TRANSLATION
RESPONSE TO UV−B
REGULATION OF CYTOPLASMIC TRANSLATION
REGULATION OF NUCLEAR−TRANSCRIBED MRNA CATABOLIC PROCESS, DEADENYLATION−DEPENDENT DECAY
BIOCARTA_GSK3_PATHWAY
DETECTION OF MECHANICAL STIMULUS INVOLVED IN SENSORY PERCEPTION OF PAIN
PROTEIN LOCALIZATION TO GOLGI APPARATUS
NEGATIVE REGULATION OF ANOIKIS
HEMATOPOIETIC STEM CELL PROLIFERATION
REGULATION OF CENTRIOLE REPLICATION
BIOCARTA_CDMAC_PATHWAY
OSTEOBLAST DEVELOPMENT
REGULATION OF ANIMAL ORGAN FORMATION
MITOTIC G1/S TRANSITION CHECKPOINT SIGNALING
MITOTIC G1 DNA DAMAGE CHECKPOINT SIGNALING
REGULATION OF KERATINOCYTE MIGRATION
MITOCHONDRION TRANSPORT ALONG MICROTUBULE
ESTABLISHMENT OF MITOCHONDRION LOCALIZATION, MICROTUBULE−MEDIATED
SENSORY SYSTEM DEVELOPMENT
REGULATION OF MACROPHAGE CHEMOTAXIS
POSITIVE REGULATION OF MACROPHAGE CHEMOTAXIS
CHEMICAL SYNAPTIC TRANSMISSION
ANTEROGRADE TRANS−SYNAPTIC SIGNALING
ATRIAL SEPTUM DEVELOPMENT
MIDBRAIN DOPAMINERGIC NEURON DIFFERENTIATION
PTERIDINE−CONTAINING COMPOUND METABOLIC PROCESS
POSITIVE REGULATION OF VASCULAR ENDOTHELIAL GROWTH FACTOR RECEPTOR SIGNALING PATHWAY
G PROTEIN−COUPLED RECEPTOR SIGNALING PATHWAY, COUPLED TO CYCLIC NUCLEOTIDE SECOND MESSENGER
HEART TRABECULA MORPHOGENESIS
PID_RXR_VDR_PATHWAY
PID_RXR_VDR_PATHWAY
PID_RETINOIC_ACID_PATHWAY
PID_RETINOIC_ACID_PATHWAY
POSITIVE REGULATION OF TRANSCRIPTION REGULATORY REGION DNA BINDING
REGULATION OF ENDOPEPTIDASE ACTIVITY
ACTIN FILAMENT POLYMERIZATION
PROTEIN POLYMERIZATION
STRIATED MUSCLE CELL DEVELOPMENT
MYOFIBRIL ASSEMBLY
Porphyrin and chlorophyll metabolism
Porphyrin and chlorophyll metabolism
MUSCLE ORGAN DEVELOPMENT
BICARBONATE TRANSPORT
EARLY.10
EARLY.11
LATE.7
EARLY.20
LATE.4
EARLY.8
LATE.8
EARLY.3
EARLY.2
LATE.1
LATE.6
EARLY.7
EARLY.21
LATE
LATE.5
EARLY.13
EARLY.15
EARLY.18
EARLY.4
EARLY.17
EARLY.12
EARLY
EARLY.5
EARLY.19
LATE.2
EARLY.6
EARLY.16
LATE.9
EARLY.1
EARLY.14
LATE.3
EARLY.9
EARLY.23
LATE.13
LATE.21
EARLY.30
EARLY.26
LATE.17
LATE.19
EARLY.22
EARLY.25
LATE.12
EARLY.28
EARLY.31
LATE.11
LATE.20
LATE.15
LATE.14
LATE.22
LATE.10
EARLY.24
EARLY.27
LATE.16
LATE.18
EARLY.29

StellarPath patient's centralities: KICH

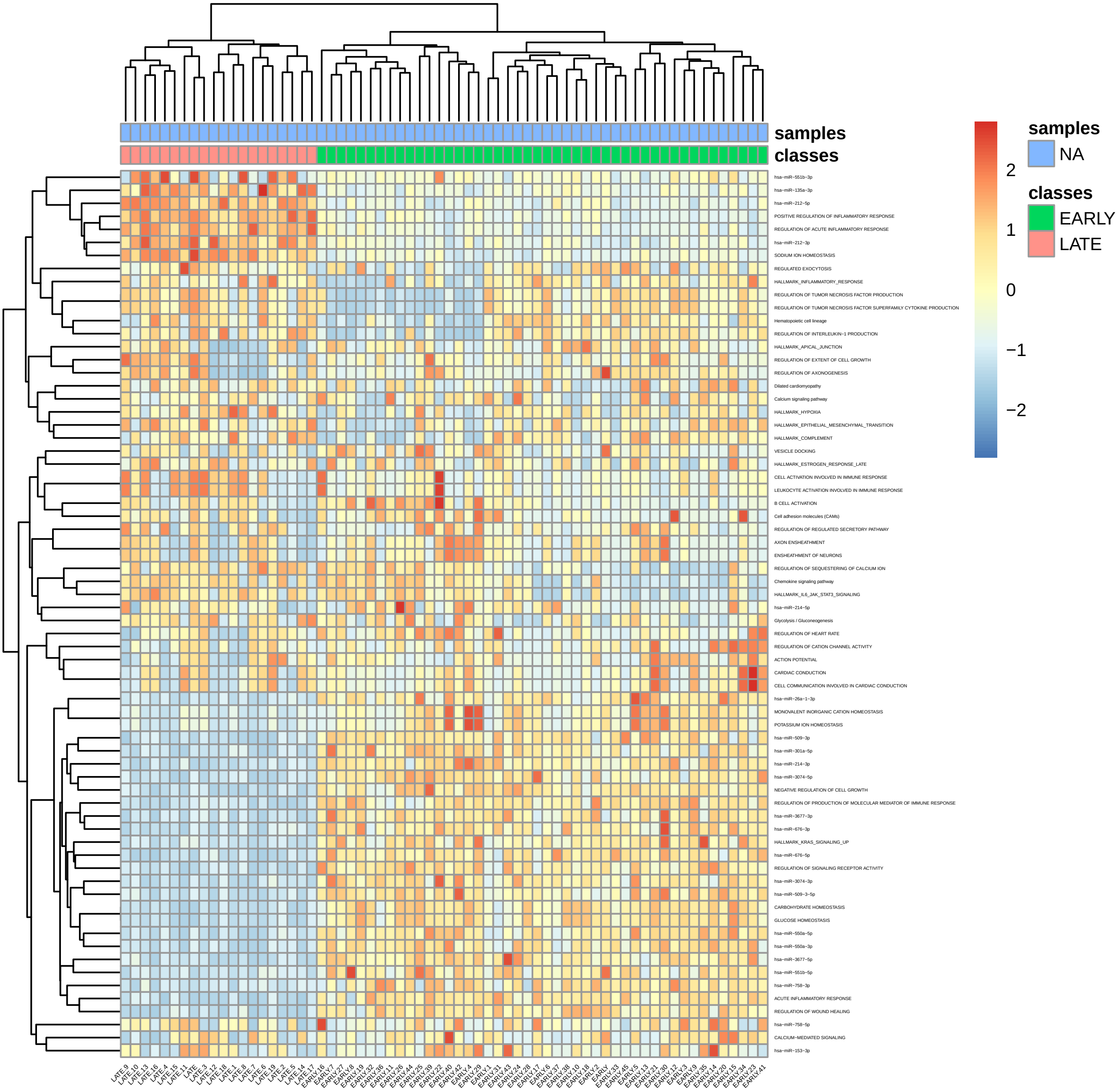

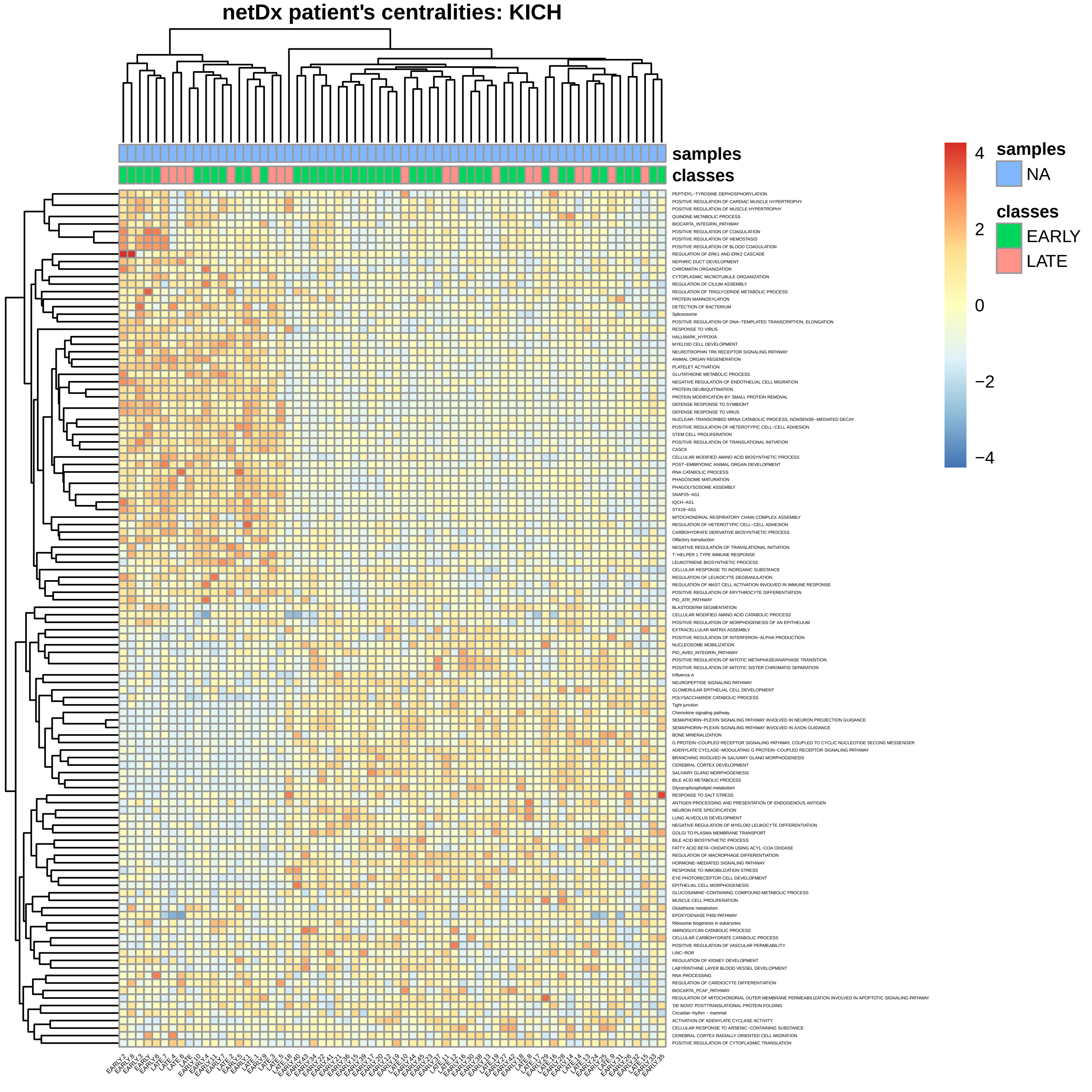

netDx patient's centralities: KICH
samples
classes
samples
NA
classes
EARLY
LATE
4
2
0
−2
−4
PEPTIDYL−TYROSINE DEPHOSPHORYLATION
POSITIVE REGULATION OF CARDIAC MUSCLE HYPERTROPHY
POSITIVE REGULATION OF MUSCLE HYPERTROPHY
QUINONE METABOLIC PROCESS
BIOCARTA_INTEGRIN_PATHWAY
POSITIVE REGULATION OF COAGULATION
POSITIVE REGULATION OF HEMOSTASIS
POSITIVE REGULATION OF BLOOD COAGULATION
REGULATION OF ERK1 AND ERK2 CASCADE
NEPHRIC DUCT DEVELOPMENT
CHROMATIN ORGANIZATION
CYTOPLASMIC MICROTUBULE ORGANIZATION
REGULATION OF CILIUM ASSEMBLY
REGULATION OF TRIGLYCERIDE METABOLIC PROCESS
PROTEIN MANNOSYLATION
DETECTION OF BACTERIUM
Spliceosome
POSITIVE REGULATION OF DNA−TEMPLATED TRANSCRIPTION, ELONGATION
RESPONSE TO VIRUS
HALLMARK_HYPOXIA
MYELOID CELL DEVELOPMENT
NEUROTROPHIN TRK RECEPTOR SIGNALING PATHWAY
ANIMAL ORGAN REGENERATION
PLATELET ACTIVATION
GLUTATHIONE METABOLIC PROCESS
NEGATIVE REGULATION OF ENDOTHELIAL CELL MIGRATION
PROTEIN DEUBIQUITINATION
PROTEIN MODIFICATION BY SMALL PROTEIN REMOVAL
DEFENSE RESPONSE TO SYMBIONT
DEFENSE RESPONSE TO VIRUS
NUCLEAR−TRANSCRIBED MRNA CATABOLIC PROCESS, NONSENSE−MEDIATED DECAY
POSITIVE REGULATION OF HETEROTYPIC CELL−CELL ADHESION
STEM CELL PROLIFERATION
POSITIVE REGULATION OF TRANSLATIONAL INITIATION
CASC9
CELLULAR MODIFIED AMINO ACID BIOSYNTHETIC PROCESS
POST−EMBRYONIC ANIMAL ORGAN DEVELOPMENT
RNA CATABOLIC PROCESS
PHAGOSOME MATURATION
PHAGOLYSOSOME ASSEMBLY
SNAP25−AS1
IQCH−AS1
STX18−AS1
MITOCHONDRIAL RESPIRATORY CHAIN COMPLEX ASSEMBLY
REGULATION OF HETEROTYPIC CELL−CELL ADHESION
CARBOHYDRATE DERIVATIVE BIOSYNTHETIC PROCESS
Olfactory transduction
NEGATIVE REGULATION OF TRANSLATIONAL INITIATION
T−HELPER 1 TYPE IMMUNE RESPONSE
LEUKOTRIENE BIOSYNTHETIC PROCESS
CELLULAR RESPONSE TO INORGANIC SUBSTANCE
REGULATION OF LEUKOCYTE DEGRANULATION
REGULATION OF MAST CELL ACTIVATION INVOLVED IN IMMUNE RESPONSE
POSITIVE REGULATION OF ERYTHROCYTE DIFFERENTIATION
PID_ATR_PATHWAY
BLASTODERM SEGMENTATION
CELLULAR MODIFIED AMINO ACID CATABOLIC PROCESS
POSITIVE REGULATION OF MORPHOGENESIS OF AN EPITHELIUM
EXTRACELLULAR MATRIX ASSEMBLY
POSITIVE REGULATION OF INTERFERON−ALPHA PRODUCTION
NUCLEOSOME MOBILIZATION
PID_AVB3_INTEGRIN_PATHWAY
POSITIVE REGULATION OF MITOTIC METAPHASE/ANAPHASE TRANSITION
POSITIVE REGULATION OF MITOTIC SISTER CHROMATID SEPARATION
Influenza A
NEUROPEPTIDE SIGNALING PATHWAY
GLOMERULAR EPITHELIAL CELL DEVELOPMENT
POLYSACCHARIDE CATABOLIC PROCESS
Tight junction
Chemokine signaling pathway
SEMAPHORIN−PLEXIN SIGNALING PATHWAY INVOLVED IN NEURON PROJECTION GUIDANCE
SEMAPHORIN−PLEXIN SIGNALING PATHWAY INVOLVED IN AXON GUIDANCE
BONE MINERALIZATION
G PROTEIN−COUPLED RECEPTOR SIGNALING PATHWAY, COUPLED TO CYCLIC NUCLEOTIDE SECOND MESSENGER
ADENYLATE CYCLASE−MODULATING G PROTEIN−COUPLED RECEPTOR SIGNALING PATHWAY
BRANCHING INVOLVED IN SALIVARY GLAND MORPHOGENESIS
CEREBRAL CORTEX DEVELOPMENT
SALIVARY GLAND MORPHOGENESIS
BILE ACID METABOLIC PROCESS
Glycerophospholipid metabolism
RESPONSE TO SALT STRESS
ANTIGEN PROCESSING AND PRESENTATION OF ENDOGENOUS ANTIGEN
NEURON FATE SPECIFICATION
LUNG ALVEOLUS DEVELOPMENT
NEGATIVE REGULATION OF MYELOID LEUKOCYTE DIFFERENTIATION
GOLGI TO PLASMA MEMBRANE TRANSPORT
BILE ACID BIOSYNTHETIC PROCESS
FATTY ACID BETA−OXIDATION USING ACYL−COA OXIDASE
REGULATION OF MACROPHAGE DIFFERENTIATION
HORMONE−MEDIATED SIGNALING PATHWAY
RESPONSE TO IMMOBILIZATION STRESS
EYE PHOTORECEPTOR CELL DEVELOPMENT
EPITHELIAL CELL MORPHOGENESIS
GLUCOSAMINE−CONTAINING COMPOUND METABOLIC PROCESS
MUSCLE CELL PROLIFERATION
Glutathione metabolism
EPOXYGENASE P450 PATHWAY
Ribosome biogenesis in eukaryotes
AMINOGLYCAN CATABOLIC PROCESS
CELLULAR CARBOHYDRATE CATABOLIC PROCESS
POSITIVE REGULATION OF VASCULAR PERMEABILITY
LINC−ROR
REGULATION OF KIDNEY DEVELOPMENT
LABYRINTHINE LAYER BLOOD VESSEL DEVELOPMENT
RNA PROCESSING
REGULATION OF CARDIOCYTE DIFFERENTIATION
BIOCARTA_PCAF_PATHWAY
REGULATION OF MITOCHONDRIAL OUTER MEMBRANE PERMEABILIZATION INVOLVED IN APOPTOTIC SIGNALING PATHWAY
'DE NOVO' POSTTRANSLATIONAL PROTEIN FOLDING
Circadian rhythm − mammal
ACTIVATION OF ADENYLATE CYCLASE ACTIVITY
CELLULAR RESPONSE TO ARSENIC−CONTAINING SUBSTANCE
CEREBRAL CORTEX RADIALLY ORIENTED CELL MIGRATION
POSITIVE REGULATION OF CYTOPLASMIC TRANSLATION

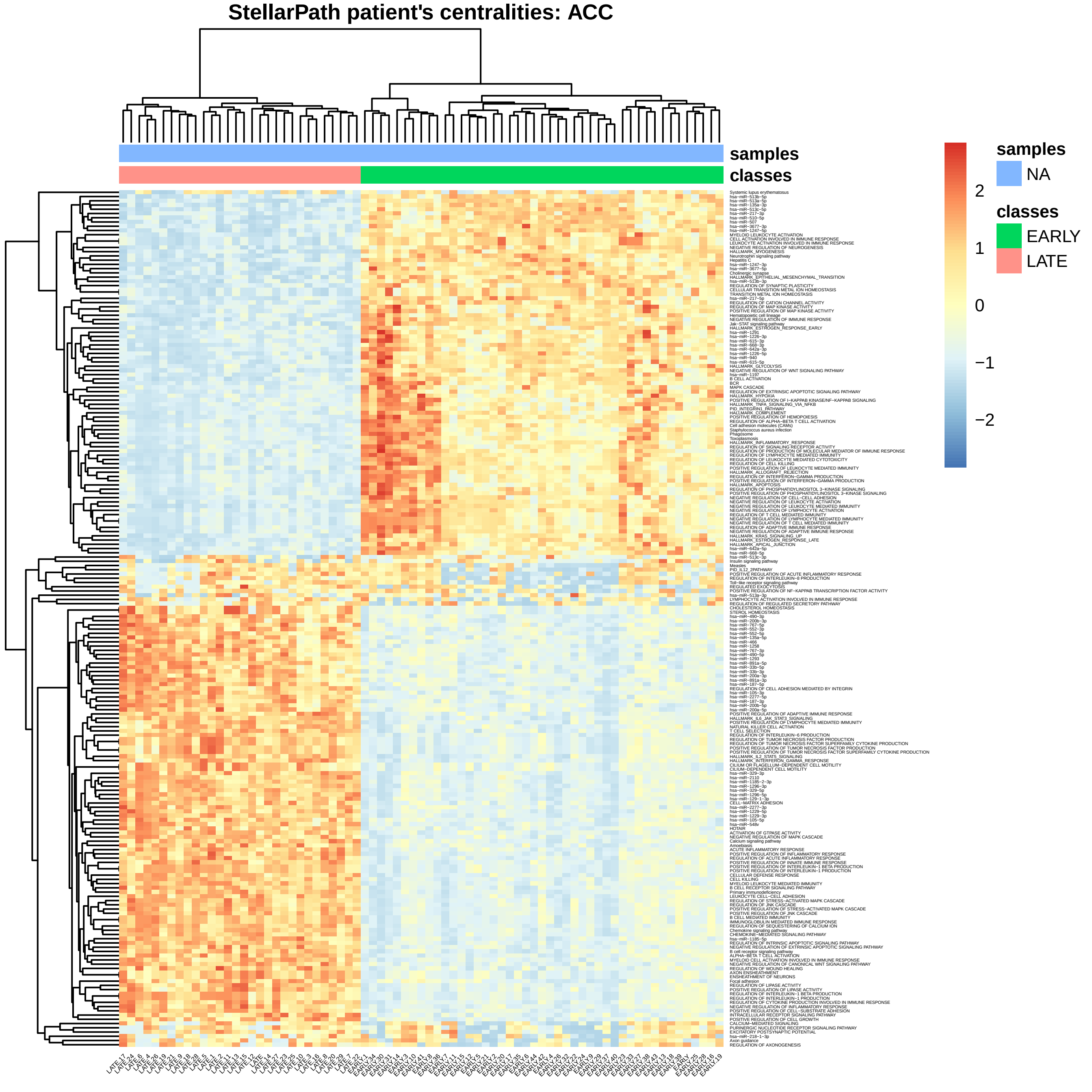
StellarPath patient's centralities: ACC
samples
classes
samples
NA
classes
EARLY
LATE
2
1
0
−1
−2

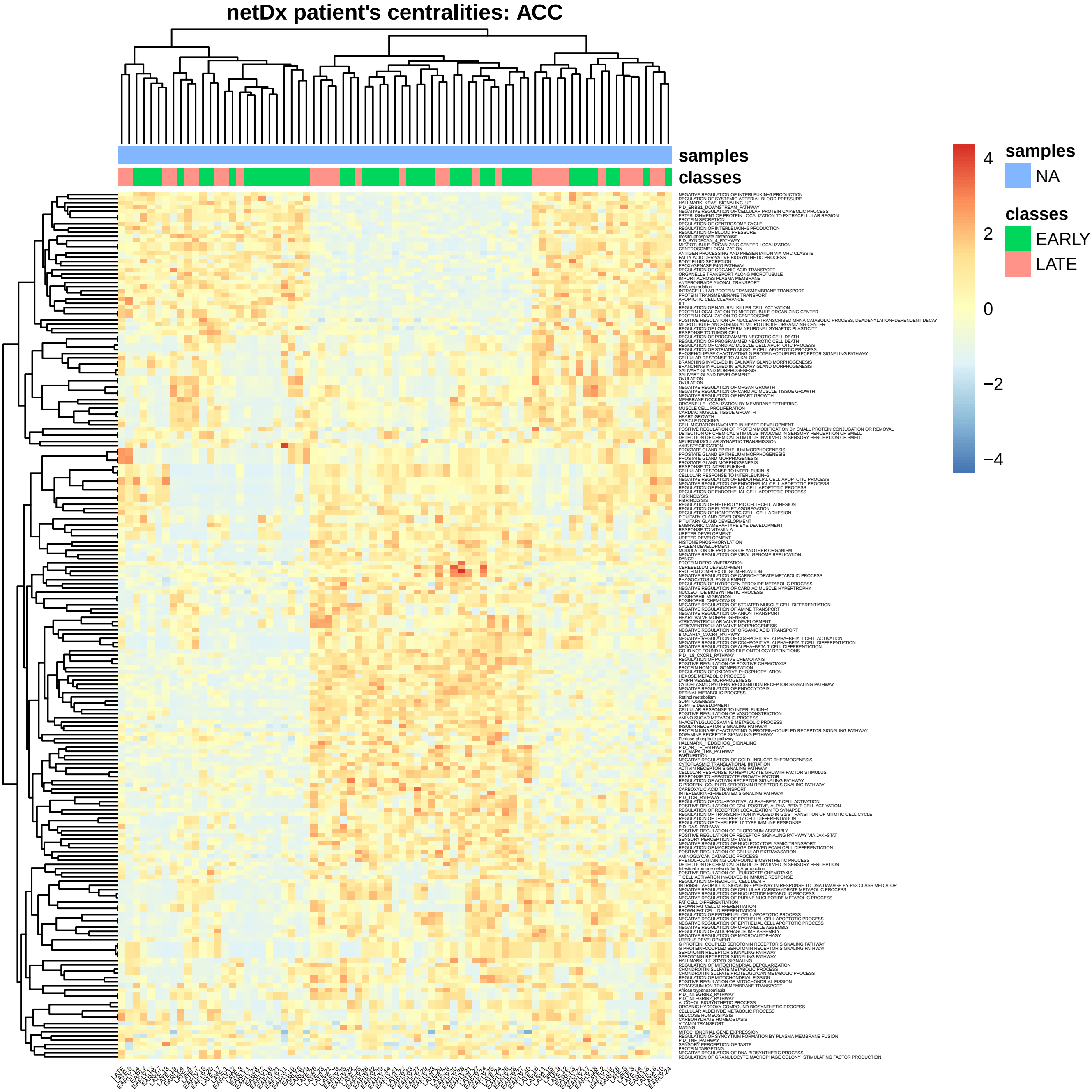
netDx patient's centralities: ACC
samples
classes
4
2
0
−2
−4
samples
NA
classes
EARLY
LATE

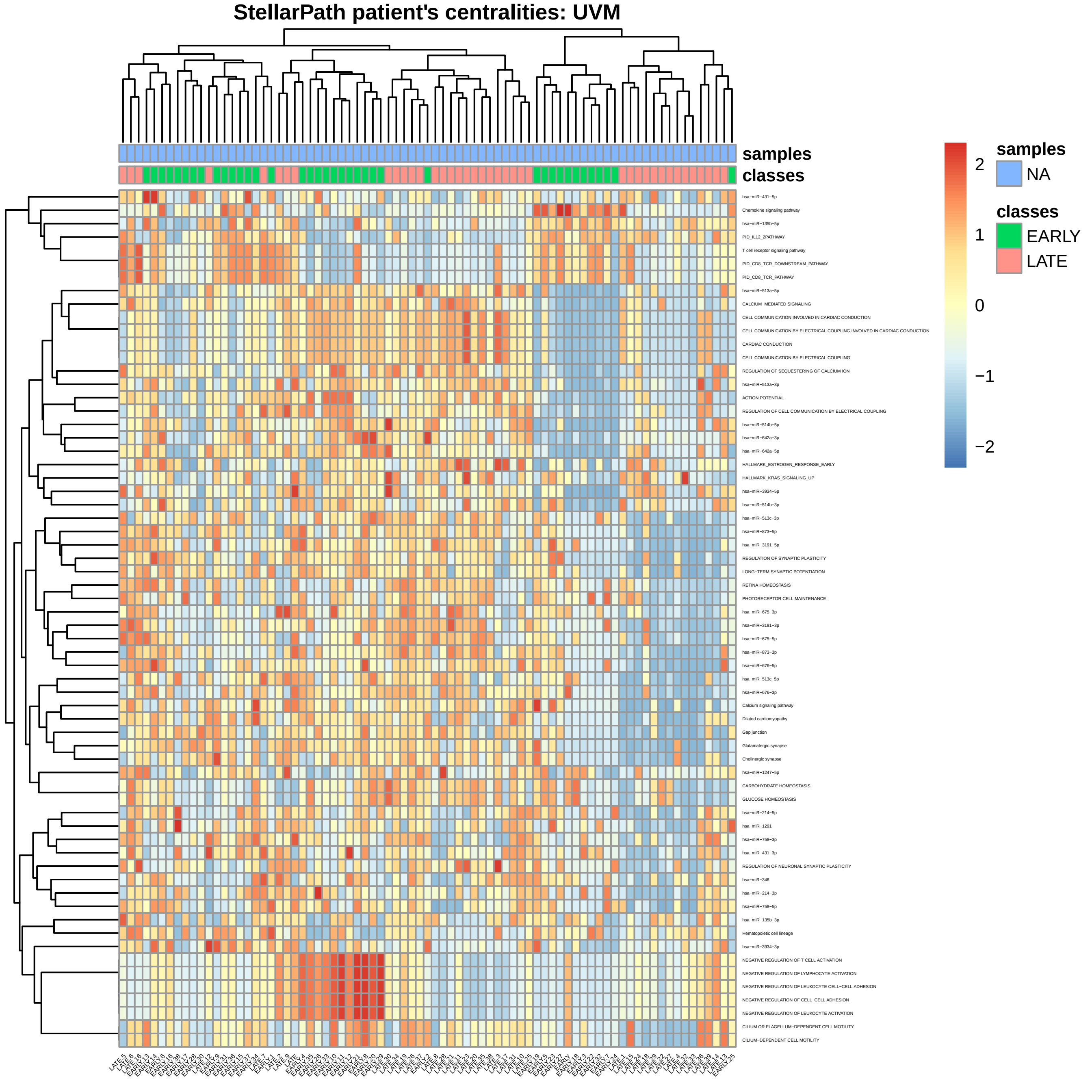
StellarPath patient's centralities: UVM
samples
classes
samples
NA
classes
EARLY
LATE
2
1
0
−1
−2
hsa−miR−431−5p
Chemokine signaling pathway
hsa−miR−135b−5p
PID_IL12_2PATHWAY
T cell receptor signaling pathway
PID_CD8_TCR_DOWNSTREAM_PATHWAY
PID_CD8_TCR_PATHWAY
hsa−miR−513a−5p
CALCIUM−MEDIATED SIGNALING
CELL COMMUNICATION INVOLVED IN CARDIAC CONDUCTION
CELL COMMUNICATION BY ELECTRICAL COUPLING INVOLVED IN CARDIAC CONDUCTION
CARDIAC CONDUCTION
CELL COMMUNICATION BY ELECTRICAL COUPLING
REGULATION OF SEQUESTERING OF CALCIUM ION
hsa−miR−513a−3p
ACTION POTENTIAL
REGULATION OF CELL COMMUNICATION BY ELECTRICAL COUPLING
hsa−miR−514b−5p
hsa−miR−642a−3p
hsa−miR−642a−5p
HALLMARK_ESTROGEN_RESPONSE_EARLY
HALLMARK_KRAS_SIGNALING_UP
hsa−miR−3934−5p
hsa−miR−514b−3p
hsa−miR−513c−3p
hsa−miR−873−5p
hsa−miR−3191−5p
REGULATION OF SYNAPTIC PLASTICITY
LONG−TERM SYNAPTIC POTENTIATION
RETINA HOMEOSTASIS
PHOTORECEPTOR CELL MAINTENANCE
hsa−miR−675−3p
hsa−miR−3191−3p
hsa−miR−675−5p
hsa−miR−873−3p
hsa−miR−676−5p
hsa−miR−513c−5p
hsa−miR−676−3p
Calcium signaling pathway
Dilated cardiomyopathy
Gap junction
Glutamatergic synapse
Cholinergic synapse
hsa−miR−1247−5p
CARBOHYDRATE HOMEOSTASIS
GLUCOSE HOMEOSTASIS
hsa−miR−214−5p
hsa−miR−1291
hsa−miR−758−3p
hsa−miR−431−3p
REGULATION OF NEURONAL SYNAPTIC PLASTICITY
hsa−miR−346
hsa−miR−214−3p
hsa−miR−758−5p
hsa−miR−135b−3p
Hematopoietic cell lineage
hsa−miR−3934−3p
NEGATIVE REGULATION OF T CELL ACTIVATION
NEGATIVE REGULATION OF LYMPHOCYTE ACTIVATION
NEGATIVE REGULATION OF LEUKOCYTE CELL−CELL ADHESION
NEGATIVE REGULATION OF CELL−CELL ADHESION
NEGATIVE REGULATION OF LEUKOCYTE ACTIVATION
CILIUM OR FLAGELLUM−DEPENDENT CELL MOTILITY
CILIUM−DEPENDENT CELL MOTILITY

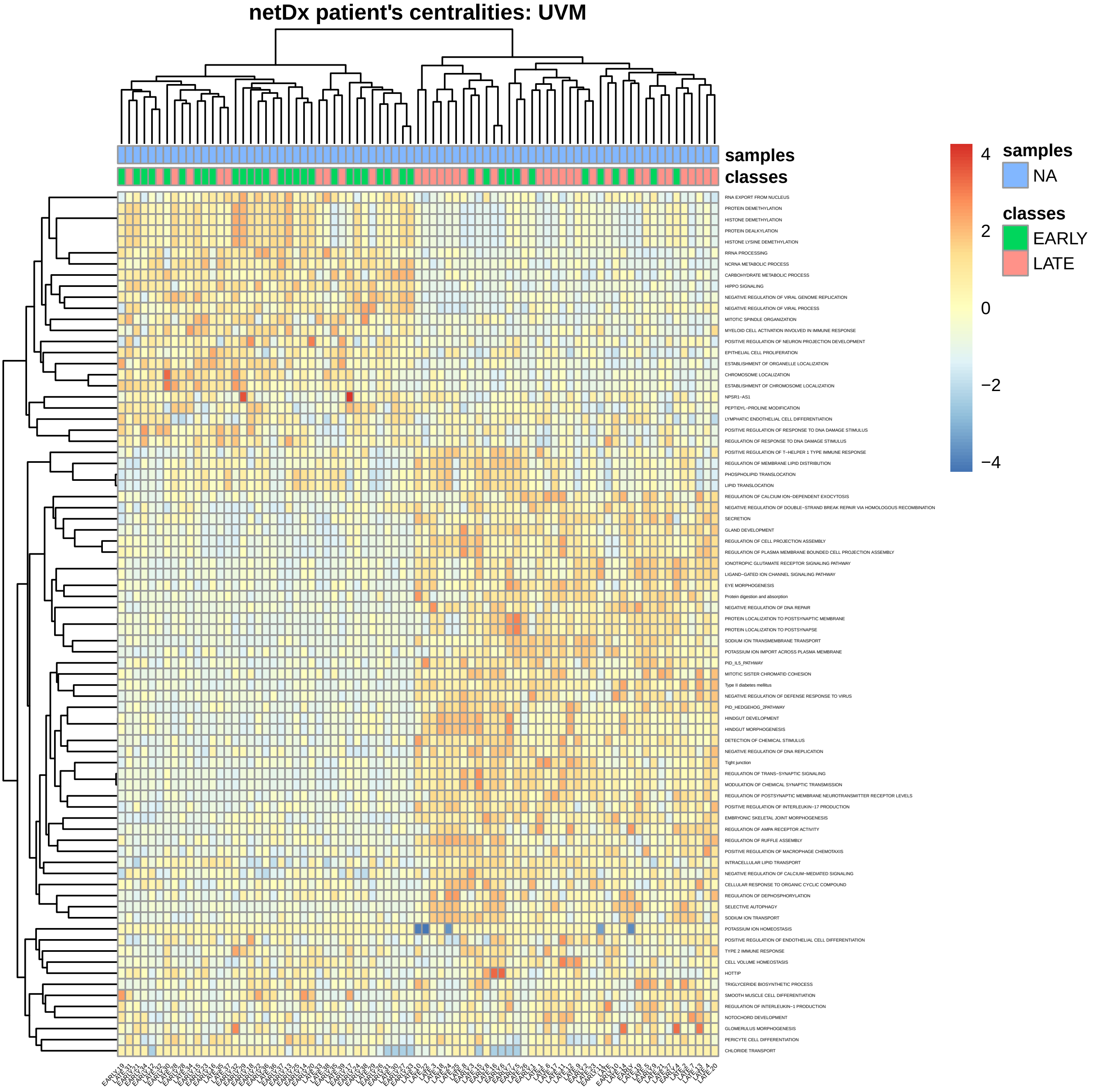
netDx patient's centralities: UVM
samples
classes
samples
NA
classes
EARLY
LATE
4
2
0
−2
−4
RNA EXPORT FROM NUCLEUS
PROTEIN DEMETHYLATION
HISTONE DEMETHYLATION
PROTEIN DEALKYLATION
HISTONE LYSINE DEMETHYLATION
RRNA PROCESSING
NCRNA METABOLIC PROCESS
CARBOHYDRATE METABOLIC PROCESS
HIPPO SIGNALING
NEGATIVE REGULATION OF VIRAL GENOME REPLICATION
NEGATIVE REGULATION OF VIRAL PROCESS
MITOTIC SPINDLE ORGANIZATION
MYELOID CELL ACTIVATION INVOLVED IN IMMUNE RESPONSE
POSITIVE REGULATION OF NEURON PROJECTION DEVELOPMENT
EPITHELIAL CELL PROLIFERATION
ESTABLISHMENT OF ORGANELLE LOCALIZATION
CHROMOSOME LOCALIZATION
ESTABLISHMENT OF CHROMOSOME LOCALIZATION
NPSR1−AS1
PEPTIDYL−PROLINE MODIFICATION
LYMPHATIC ENDOTHELIAL CELL DIFFERENTIATION
POSITIVE REGULATION OF RESPONSE TO DNA DAMAGE STIMULUS
REGULATION OF RESPONSE TO DNA DAMAGE STIMULUS
POSITIVE REGULATION OF T−HELPER 1 TYPE IMMUNE RESPONSE
REGULATION OF MEMBRANE LIPID DISTRIBUTION
PHOSPHOLIPID TRANSLOCATION
LIPID TRANSLOCATION
REGULATION OF CALCIUM ION−DEPENDENT EXOCYTOSIS
NEGATIVE REGULATION OF DOUBLE−STRAND BREAK REPAIR VIA HOMOLOGOUS RECOMBINATION
SECRETION
GLAND DEVELOPMENT
REGULATION OF CELL PROJECTION ASSEMBLY
REGULATION OF PLASMA MEMBRANE BOUNDED CELL PROJECTION ASSEMBLY
IONOTROPIC GLUTAMATE RECEPTOR SIGNALING PATHWAY
LIGAND−GATED ION CHANNEL SIGNALING PATHWAY
EYE MORPHOGENESIS
Protein digestion and absorption
NEGATIVE REGULATION OF DNA REPAIR
PROTEIN LOCALIZATION TO POSTSYNAPTIC MEMBRANE
PROTEIN LOCALIZATION TO POSTSYNAPSE
SODIUM ION TRANSMEMBRANE TRANSPORT
POTASSIUM ION IMPORT ACROSS PLASMA MEMBRANE
PID_IL5_PATHWAY
MITOTIC SISTER CHROMATID COHESION
Type II diabetes mellitus
NEGATIVE REGULATION OF DEFENSE RESPONSE TO VIRUS
PID_HEDGEHOG_2PATHWAY
HINDGUT DEVELOPMENT
HINDGUT MORPHOGENESIS
DETECTION OF CHEMICAL STIMULUS
NEGATIVE REGULATION OF DNA REPLICATION
Tight junction
REGULATION OF TRANS−SYNAPTIC SIGNALING
MODULATION OF CHEMICAL SYNAPTIC TRANSMISSION
REGULATION OF POSTSYNAPTIC MEMBRANE NEUROTRANSMITTER RECEPTOR LEVELS
POSITIVE REGULATION OF INTERLEUKIN−17 PRODUCTION
EMBRYONIC SKELETAL JOINT MORPHOGENESIS
REGULATION OF AMPA RECEPTOR ACTIVITY
REGULATION OF RUFFLE ASSEMBLY
POSITIVE REGULATION OF MACROPHAGE CHEMOTAXIS
INTRACELLULAR LIPID TRANSPORT
NEGATIVE REGULATION OF CALCIUM−MEDIATED SIGNALING
CELLULAR RESPONSE TO ORGANIC CYCLIC COMPOUND
REGULATION OF DEPHOSPHORYLATION
SELECTIVE AUTOPHAGY
SODIUM ION TRANSPORT
POTASSIUM ION HOMEOSTASIS
POSITIVE REGULATION OF ENDOTHELIAL CELL DIFFERENTIATION
TYPE 2 IMMUNE RESPONSE
CELL VOLUME HOMEOSTASIS
HOTTIP
TRIGLYCERIDE BIOSYNTHETIC PROCESS
SMOOTH MUSCLE CELL DIFFERENTIATION
REGULATION OF INTERLEUKIN−1 PRODUCTION
NOTOCHORD DEVELOPMENT
GLOMERULUS MORPHOGENESIS
PERICYTE CELL DIFFERENTIATION
CHLORIDE TRANSPORT

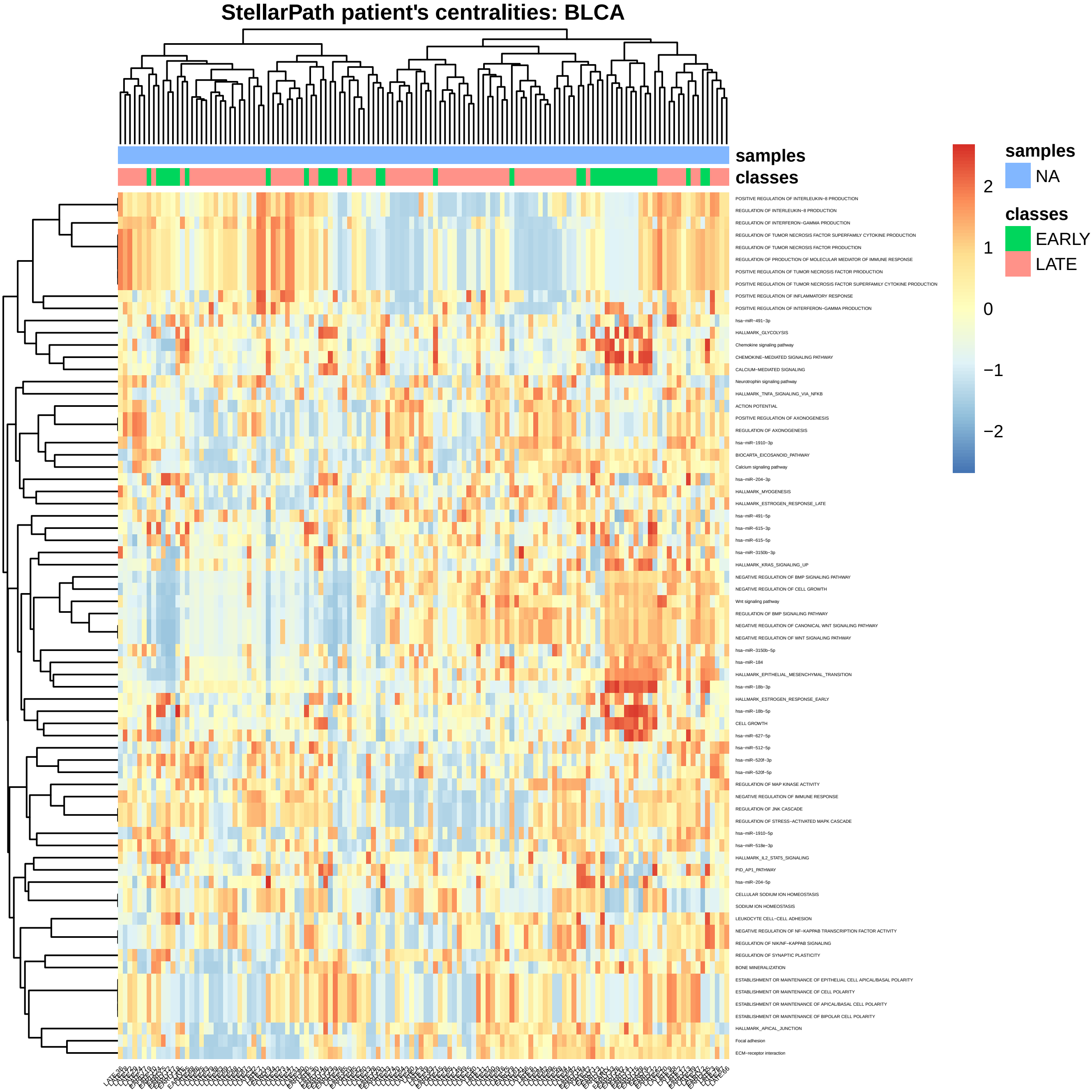
StellarPath patient's centralities: BLCA
samples
classes
samples
NA
classes
EARLY
LATE
2
1
0
−1
−2
POSITIVE REGULATION OF INTERLEUKIN−8 PRODUCTION
REGULATION OF INTERLEUKIN−8 PRODUCTION
REGULATION OF INTERFERON−GAMMA PRODUCTION
REGULATION OF TUMOR NECROSIS FACTOR SUPERFAMILY CYTOKINE PRODUCTION
REGULATION OF TUMOR NECROSIS FACTOR PRODUCTION
REGULATION OF PRODUCTION OF MOLECULAR MEDIATOR OF IMMUNE RESPONSE
POSITIVE REGULATION OF TUMOR NECROSIS FACTOR PRODUCTION
POSITIVE REGULATION OF TUMOR NECROSIS FACTOR SUPERFAMILY CYTOKINE PRODUCTION
POSITIVE REGULATION OF INFLAMMATORY RESPONSE
POSITIVE REGULATION OF INTERFERON−GAMMA PRODUCTION
hsa−miR−491−3p
HALLMARK_GLYCOLYSIS
Chemokine signaling pathway
CHEMOKINE−MEDIATED SIGNALING PATHWAY
CALCIUM−MEDIATED SIGNALING
Neurotrophin signaling pathway
HALLMARK_TNFA_SIGNALING_VIA_NFKB
ACTION POTENTIAL
POSITIVE REGULATION OF AXONOGENESIS
REGULATION OF AXONOGENESIS
hsa−miR−1910−3p
BIOCARTA_EICOSANOID_PATHWAY
Calcium signaling pathway
hsa−miR−204−3p
HALLMARK_MYOGENESIS
HALLMARK_ESTROGEN_RESPONSE_LATE
hsa−miR−491−5p
hsa−miR−615−3p
hsa−miR−615−5p
hsa−miR−3150b−3p
HALLMARK_KRAS_SIGNALING_UP
NEGATIVE REGULATION OF BMP SIGNALING PATHWAY
NEGATIVE REGULATION OF CELL GROWTH
Wnt signaling pathway
REGULATION OF BMP SIGNALING PATHWAY
NEGATIVE REGULATION OF CANONICAL WNT SIGNALING PATHWAY
NEGATIVE REGULATION OF WNT SIGNALING PATHWAY
hsa−miR−3150b−5p
hsa−miR−184
HALLMARK_EPITHELIAL_MESENCHYMAL_TRANSITION
hsa−miR−18b−3p
HALLMARK_ESTROGEN_RESPONSE_EARLY
hsa−miR−18b−5p
CELL GROWTH
hsa−miR−627−5p
hsa−miR−512−5p
hsa−miR−520f−3p
hsa−miR−520f−5p
REGULATION OF MAP KINASE ACTIVITY
NEGATIVE REGULATION OF IMMUNE RESPONSE
REGULATION OF JNK CASCADE
REGULATION OF STRESS−ACTIVATED MAPK CASCADE
hsa−miR−1910−5p
hsa−miR−518e−3p
HALLMARK_IL2_STAT5_SIGNALING
PID_AP1_PATHWAY
hsa−miR−204−5p
CELLULAR SODIUM ION HOMEOSTASIS
SODIUM ION HOMEOSTASIS
LEUKOCYTE CELL−CELL ADHESION
NEGATIVE REGULATION OF NF−KAPPAB TRANSCRIPTION FACTOR ACTIVITY
REGULATION OF NIK/NF−KAPPAB SIGNALING
REGULATION OF SYNAPTIC PLASTICITY
BONE MINERALIZATION
ESTABLISHMENT OR MAINTENANCE OF EPITHELIAL CELL APICAL/BASAL POLARITY
ESTABLISHMENT OR MAINTENANCE OF CELL POLARITY
ESTABLISHMENT OR MAINTENANCE OF APICAL/BASAL CELL POLARITY
ESTABLISHMENT OR MAINTENANCE OF BIPOLAR CELL POLARITY
HALLMARK_APICAL_JUNCTION
Focal adhesion
ECM−receptor interaction

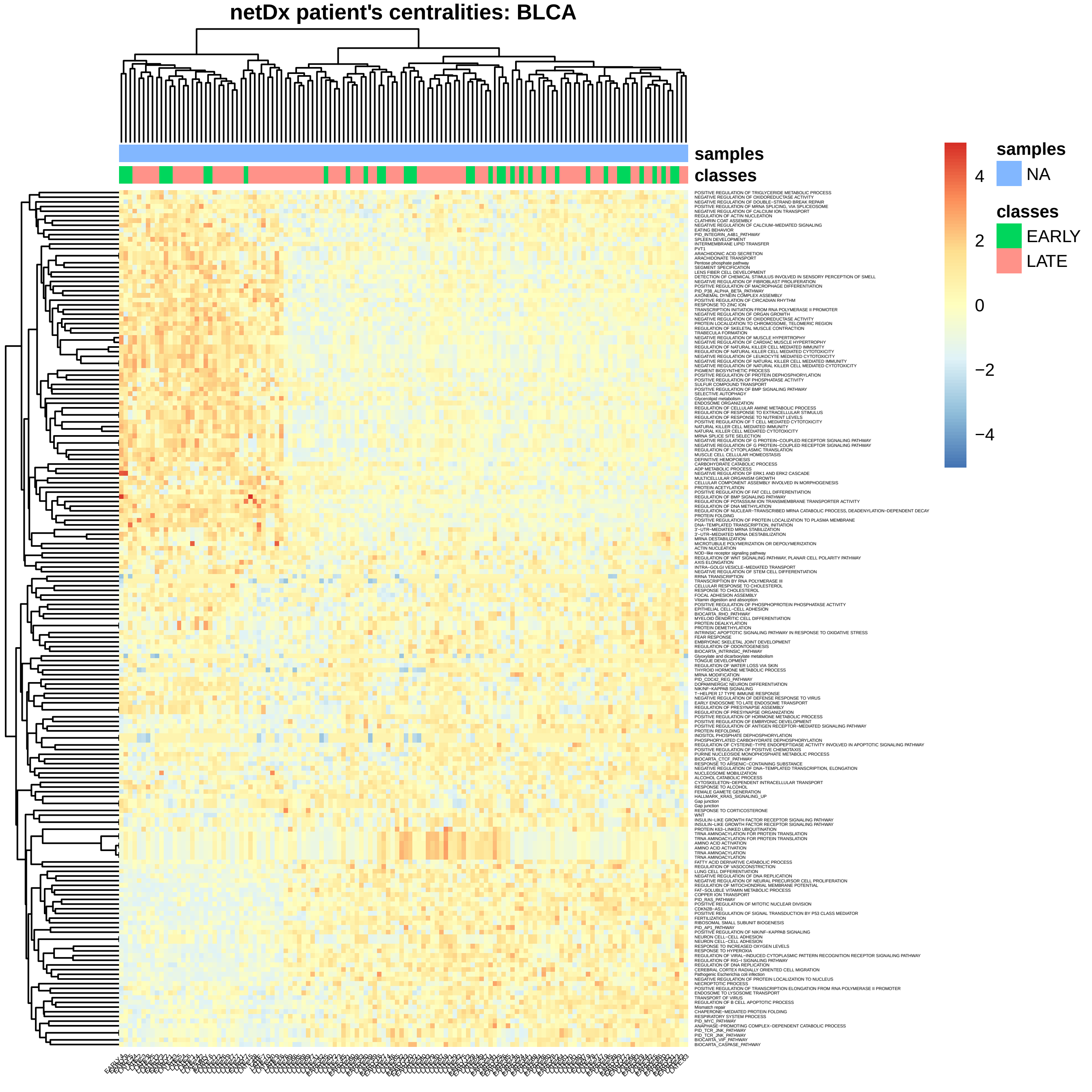
netDx patient's centralities: BLCA
samples
classes
samples
NA
classes
EARLY
LATE
4
2
0
−2
−4
POSITIVE REGULATION OF TRIGLYCERIDE METABOLIC PROCESS
NEGATIVE REGULATION OF OXIDOREDUCTASE ACTIVITY
NEGATIVE REGULATION OF DOUBLE−STRAND BREAK REPAIR
POSITIVE REGULATION OF MRNA SPLICING, VIA SPLICEOSOME
NEGATIVE REGULATION OF CALCIUM ION TRANSPORT
REGULATION OF ACTIN NUCLEATION
CLATHRIN COAT ASSEMBLY
NEGATIVE REGULATION OF CALCIUM−MEDIATED SIGNALING
EATING BEHAVIOR
PID_INTEGRIN_A4B1_PATHWAY
SPLEEN DEVELOPMENT
INTERMEMBRANE LIPID TRANSFER
PVT1
ARACHIDONIC ACID SECRETION
ARACHIDONATE TRANSPORT
Pentose phosphate pathway
SEGMENT SPECIFICATION
LENS FIBER CELL DEVELOPMENT
DETECTION OF CHEMICAL STIMULUS INVOLVED IN SENSORY PERCEPTION OF SMELL
NEGATIVE REGULATION OF FIBROBLAST PROLIFERATION
POSITIVE REGULATION OF MACROPHAGE DIFFERENTIATION
PID_P38_ALPHA_BETA_PATHWAY
AXONEMAL DYNEIN COMPLEX ASSEMBLY
POSITIVE REGULATION OF CIRCADIAN RHYTHM
RESPONSE TO ZINC ION
TRANSCRIPTION INITIATION FROM RNA POLYMERASE II PROMOTER
NEGATIVE REGULATION OF ORGAN GROWTH
NEGATIVE REGULATION OF OXIDOREDUCTASE ACTIVITY
PROTEIN LOCALIZATION TO CHROMOSOME, TELOMERIC REGION
REGULATION OF SKELETAL MUSCLE CONTRACTION
TRABECULA FORMATION
NEGATIVE REGULATION OF MUSCLE HYPERTROPHY
NEGATIVE REGULATION OF CARDIAC MUSCLE HYPERTROPHY
REGULATION OF NATURAL KILLER CELL MEDIATED IMMUNITY
REGULATION OF NATURAL KILLER CELL MEDIATED CYTOTOXICITY
NEGATIVE REGULATION OF LEUKOCYTE MEDIATED CYTOTOXICITY
NEGATIVE REGULATION OF NATURAL KILLER CELL MEDIATED IMMUNITY
NEGATIVE REGULATION OF NATURAL KILLER CELL MEDIATED CYTOTOXICITY
PIGMENT BIOSYNTHETIC PROCESS
POSITIVE REGULATION OF PROTEIN DEPHOSPHORYLATION
POSITIVE REGULATION OF PHOSPHATASE ACTIVITY
SULFUR COMPOUND TRANSPORT
POSITIVE REGULATION OF BMP SIGNALING PATHWAY
SELECTIVE AUTOPHAGY
Glycerolipid metabolism
ENDOSOME ORGANIZATION
REGULATION OF CELLULAR AMINE METABOLIC PROCESS
REGULATION OF RESPONSE TO EXTRACELLULAR STIMULUS
REGULATION OF RESPONSE TO NUTRIENT LEVELS
POSITIVE REGULATION OF T CELL MEDIATED CYTOTOXICITY
NATURAL KILLER CELL MEDIATED IMMUNITY
NATURAL KILLER CELL MEDIATED CYTOTOXICITY
MRNA SPLICE SITE SELECTION
NEGATIVE REGULATION OF G PROTEIN−COUPLED RECEPTOR SIGNALING PATHWAY
NEGATIVE REGULATION OF G PROTEIN−COUPLED RECEPTOR SIGNALING PATHWAY
REGULATION OF CYTOPLASMIC TRANSLATION
MUSCLE CELL CELLULAR HOMEOSTASIS
DEFINITIVE HEMOPOIESIS
CARBOHYDRATE CATABOLIC PROCESS
ADP METABOLIC PROCESS
NEGATIVE REGULATION OF ERK1 AND ERK2 CASCADE
MULTICELLULAR ORGANISM GROWTH
CELLULAR COMPONENT ASSEMBLY INVOLVED IN MORPHOGENESIS
PROTEIN ACETYLATION
POSITIVE REGULATION OF FAT CELL DIFFERENTIATION
REGULATION OF BMP SIGNALING PATHWAY
REGULATION OF POTASSIUM ION TRANSMEMBRANE TRANSPORTER ACTIVITY
REGULATION OF DNA METHYLATION
REGULATION OF NUCLEAR−TRANSCRIBED MRNA CATABOLIC PROCESS, DEADENYLATION−DEPENDENT DECAY
PROTEIN FOLDING
POSITIVE REGULATION OF PROTEIN LOCALIZATION TO PLASMA MEMBRANE
DNA−TEMPLATED TRANSCRIPTION, INITIATION
3'−UTR−MEDIATED MRNA STABILIZATION
3'−UTR−MEDIATED MRNA DESTABILIZATION
MRNA DESTABILIZATION
MICROTUBULE POLYMERIZATION OR DEPOLYMERIZATION
ACTIN NUCLEATION
NOD−like receptor signaling pathway
REGULATION OF WNT SIGNALING PATHWAY, PLANAR CELL POLARITY PATHWAY
AXIS ELONGATION
INTRA−GOLGI VESICLE−MEDIATED TRANSPORT
NEGATIVE REGULATION OF STEM CELL DIFFERENTIATION
RRNA TRANSCRIPTION
TRANSCRIPTION BY RNA POLYMERASE III
CELLULAR RESPONSE TO CHOLESTEROL
RESPONSE TO CHOLESTEROL
FOCAL ADHESION ASSEMBLY
Vitamin digestion and absorption
POSITIVE REGULATION OF PHOSPHOPROTEIN PHOSPHATASE ACTIVITY
EPITHELIAL CELL−CELL ADHESION
BIOCARTA_RHO_PATHWAY
MYELOID DENDRITIC CELL DIFFERENTIATION
PROTEIN DEALKYLATION
PROTEIN DEMETHYLATION
INTRINSIC APOPTOTIC SIGNALING PATHWAY IN RESPONSE TO OXIDATIVE STRESS
FEAR RESPONSE
EMBRYONIC SKELETAL JOINT DEVELOPMENT
REGULATION OF ODONTOGENESIS
BIOCARTA_INTRINSIC_PATHWAY
Glyoxylate and dicarboxylate metabolism
TONGUE DEVELOPMENT
REGULATION OF WATER LOSS VIA SKIN
THYROID HORMONE METABOLIC PROCESS
MRNA MODIFICATION
PID_CDC42_REG_PATHWAY
DOPAMINERGIC NEURON DIFFERENTIATION
NIK/NF−KAPPAB SIGNALING
T−HELPER 17 TYPE IMMUNE RESPONSE
NEGATIVE REGULATION OF DEFENSE RESPONSE TO VIRUS
EARLY ENDOSOME TO LATE ENDOSOME TRANSPORT
REGULATION OF PRESYNAPSE ASSEMBLY
REGULATION OF PRESYNAPSE ORGANIZATION
POSITIVE REGULATION OF HORMONE METABOLIC PROCESS
POSITIVE REGULATION OF EMBRYONIC DEVELOPMENT
POSITIVE REGULATION OF ANTIGEN RECEPTOR−MEDIATED SIGNALING PATHWAY
PROTEIN REFOLDING
INOSITOL PHOSPHATE DEPHOSPHORYLATION
PHOSPHORYLATED CARBOHYDRATE DEPHOSPHORYLATION
REGULATION OF CYSTEINE−TYPE ENDOPEPTIDASE ACTIVITY INVOLVED IN APOPTOTIC SIGNALING PATHWAY
POSITIVE REGULATION OF POSITIVE CHEMOTAXIS
PURINE NUCLEOSIDE MONOPHOSPHATE METABOLIC PROCESS
BIOCARTA_CTCF_PATHWAY
RESPONSE TO ARSENIC−CONTAINING SUBSTANCE
NEGATIVE REGULATION OF DNA−TEMPLATED TRANSCRIPTION, ELONGATION
NUCLEOSOME MOBILIZATION
ALCOHOL CATABOLIC PROCESS
CYTOSKELETON−DEPENDENT INTRACELLULAR TRANSPORT
RESPONSE TO ALCOHOL
FEMALE GAMETE GENERATION
HALLMARK_KRAS_SIGNALING_UP
Gap junction
Gap junction
RESPONSE TO CORTICOSTERONE
WNT
INSULIN−LIKE GROWTH FACTOR RECEPTOR SIGNALING PATHWAY
INSULIN−LIKE GROWTH FACTOR RECEPTOR SIGNALING PATHWAY
PROTEIN K63−LINKED UBIQUITINATION
TRNA AMINOACYLATION FOR PROTEIN TRANSLATION
TRNA AMINOACYLATION FOR PROTEIN TRANSLATION
AMINO ACID ACTIVATION
AMINO ACID ACTIVATION
TRNA AMINOACYLATION
TRNA AMINOACYLATION
FATTY ACID DERIVATIVE CATABOLIC PROCESS
REGULATION OF VASOCONSTRICTION
LUNG CELL DIFFERENTIATION
NEGATIVE REGULATION OF DNA REPLICATION
NEGATIVE REGULATION OF NEURAL PRECURSOR CELL PROLIFERATION
REGULATION OF MITOCHONDRIAL MEMBRANE POTENTIAL
FAT−SOLUBLE VITAMIN METABOLIC PROCESS
COPPER ION TRANSPORT
PID_RAS_PATHWAY
POSITIVE REGULATION OF MITOTIC NUCLEAR DIVISION
CDKN2B−AS1
POSITIVE REGULATION OF SIGNAL TRANSDUCTION BY P53 CLASS MEDIATOR
FERTILIZATION
RIBOSOMAL SMALL SUBUNIT BIOGENESIS
PID_AP1_PATHWAY
POSITIVE REGULATION OF NIK/NF−KAPPAB SIGNALING
NEURON CELL−CELL ADHESION
NEURON CELL−CELL ADHESION
RESPONSE TO INCREASED OXYGEN LEVELS
RESPONSE TO HYPEROXIA
REGULATION OF VIRAL−INDUCED CYTOPLASMIC PATTERN RECOGNITION RECEPTOR SIGNALING PATHWAY
REGULATION OF RIG−I SIGNALING PATHWAY
REGULATION OF DNA REPLICATION
CEREBRAL CORTEX RADIALLY ORIENTED CELL MIGRATION
Pathogenic Escherichia coli infection
NEGATIVE REGULATION OF PROTEIN LOCALIZATION TO NUCLEUS
NECROPTOTIC PROCESS
POSITIVE REGULATION OF TRANSCRIPTION ELONGATION FROM RNA POLYMERASE II PROMOTER
ENDOSOME TO LYSOSOME TRANSPORT
TRANSPORT OF VIRUS
REGULATION OF B CELL APOPTOTIC PROCESS
Mismatch repair
CHAPERONE−MEDIATED PROTEIN FOLDING
RESPIRATORY SYSTEM PROCESS
PID_MYC_PATHWAY
ANAPHASE−PROMOTING COMPLEX−DEPENDENT CATABOLIC PROCESS
PID_TCR_JNK_PATHWAY
PID_TCR_JNK_PATHWAY
BIOCARTA_VIP_PATHWAY
BIOCARTA_CASPASE_PATHWAY

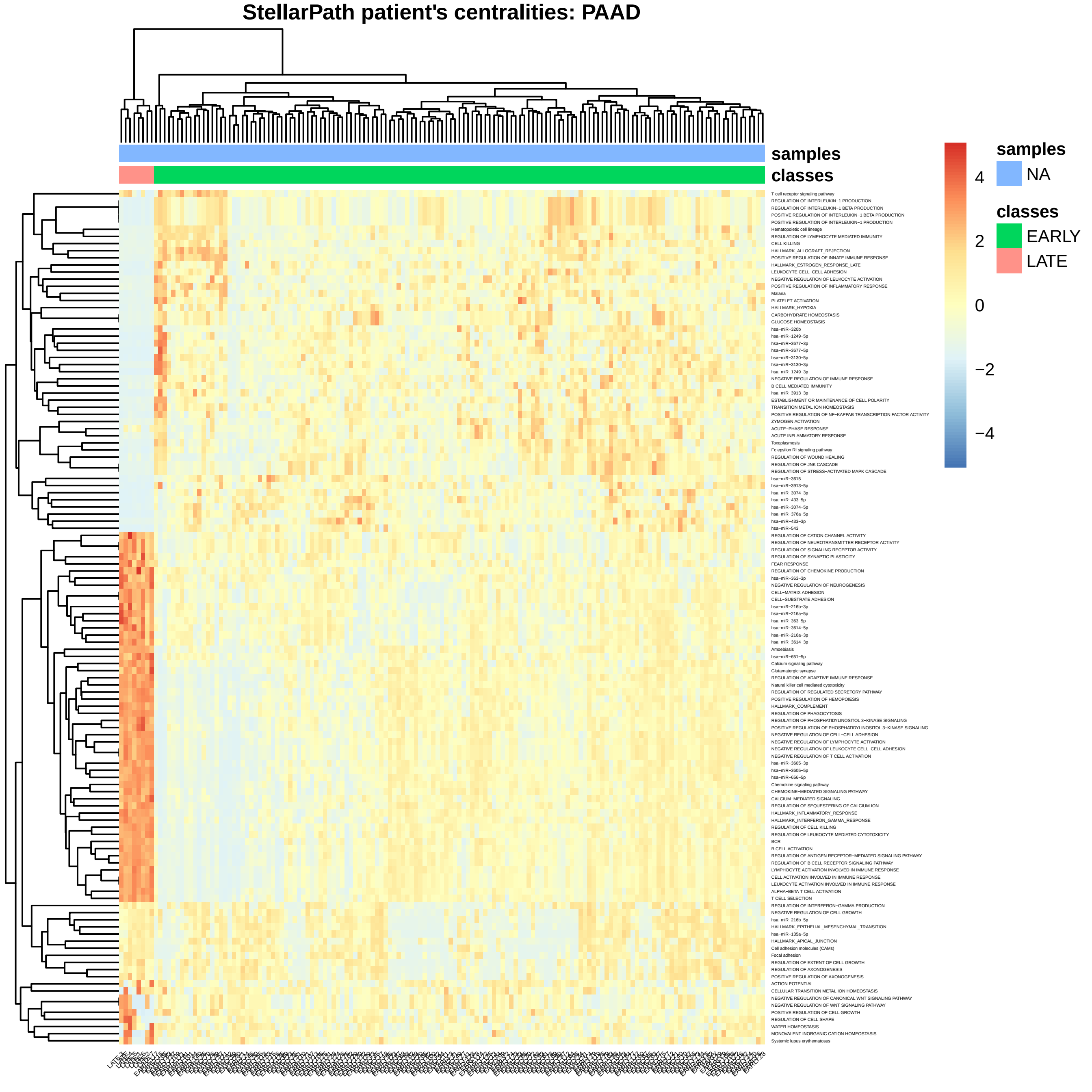
StellarPath patient's centralities: PAAD
samples
classes
4
2
0
−2
−4
samples
NA
classes
EARLY
LATE
T cell receptor signaling pathway
REGULATION OF INTERLEUKIN−1 PRODUCTION
REGULATION OF INTERLEUKIN−1 BETA PRODUCTION
POSITIVE REGULATION OF INTERLEUKIN−1 BETA PRODUCTION
POSITIVE REGULATION OF INTERLEUKIN−1 PRODUCTION
Hematopoietic cell lineage
REGULATION OF LYMPHOCYTE MEDIATED IMMUNITY
CELL KILLING
HALLMARK_ALLOGRAFT_REJECTION
POSITIVE REGULATION OF INNATE IMMUNE RESPONSE
HALLMARK_ESTROGEN_RESPONSE_LATE
LEUKOCYTE CELL−CELL ADHESION
NEGATIVE REGULATION OF LEUKOCYTE ACTIVATION
POSITIVE REGULATION OF INFLAMMATORY RESPONSE
Malaria
PLATELET ACTIVATION
HALLMARK_HYPOXIA
CARBOHYDRATE HOMEOSTASIS
GLUCOSE HOMEOSTASIS
hsa−miR−320b
hsa−miR−1249−5p
hsa−miR−3677−3p
hsa−miR−3677−5p
hsa−miR−3130−5p
hsa−miR−3130−3p
hsa−miR−1249−3p
NEGATIVE REGULATION OF IMMUNE RESPONSE
B CELL MEDIATED IMMUNITY
hsa−miR−3913−3p
ESTABLISHMENT OR MAINTENANCE OF CELL POLARITY
TRANSITION METAL ION HOMEOSTASIS
POSITIVE REGULATION OF NF−KAPPAB TRANSCRIPTION FACTOR ACTIVITY
ZYMOGEN ACTIVATION
ACUTE−PHASE RESPONSE
ACUTE INFLAMMATORY RESPONSE
Toxoplasmosis
Fc epsilon RI signaling pathway
REGULATION OF WOUND HEALING
REGULATION OF JNK CASCADE
REGULATION OF STRESS−ACTIVATED MAPK CASCADE
hsa−miR−3615
hsa−miR−3913−5p
hsa−miR−3074−3p
hsa−miR−433−5p
hsa−miR−3074−5p
hsa−miR−376a−5p
hsa−miR−433−3p
hsa−miR−543
REGULATION OF CATION CHANNEL ACTIVITY
REGULATION OF NEUROTRANSMITTER RECEPTOR ACTIVITY
REGULATION OF SIGNALING RECEPTOR ACTIVITY
REGULATION OF SYNAPTIC PLASTICITY
FEAR RESPONSE
REGULATION OF CHEMOKINE PRODUCTION
hsa−miR−363−3p
NEGATIVE REGULATION OF NEUROGENESIS
CELL−MATRIX ADHESION
CELL−SUBSTRATE ADHESION
hsa−miR−216b−3p
hsa−miR−216a−5p
hsa−miR−363−5p
hsa−miR−3614−5p
hsa−miR−216a−3p
hsa−miR−3614−3p
Amoebiasis
hsa−miR−651−5p
Calcium signaling pathway
Glutamatergic synapse
REGULATION OF ADAPTIVE IMMUNE RESPONSE
Natural killer cell mediated cytotoxicity
REGULATION OF REGULATED SECRETORY PATHWAY
POSITIVE REGULATION OF HEMOPOIESIS
HALLMARK_COMPLEMENT
REGULATION OF PHAGOCYTOSIS
REGULATION OF PHOSPHATIDYLINOSITOL 3−KINASE SIGNALING
POSITIVE REGULATION OF PHOSPHATIDYLINOSITOL 3−KINASE SIGNALING
NEGATIVE REGULATION OF CELL−CELL ADHESION
NEGATIVE REGULATION OF LYMPHOCYTE ACTIVATION
NEGATIVE REGULATION OF LEUKOCYTE CELL−CELL ADHESION
NEGATIVE REGULATION OF T CELL ACTIVATION
hsa−miR−3605−3p
hsa−miR−3605−5p
hsa−miR−656−5p
Chemokine signaling pathway
CHEMOKINE−MEDIATED SIGNALING PATHWAY
CALCIUM−MEDIATED SIGNALING
REGULATION OF SEQUESTERING OF CALCIUM ION
HALLMARK_INFLAMMATORY_RESPONSE
HALLMARK_INTERFERON_GAMMA_RESPONSE
REGULATION OF CELL KILLING
REGULATION OF LEUKOCYTE MEDIATED CYTOTOXICITY
BCR
B CELL ACTIVATION
REGULATION OF ANTIGEN RECEPTOR−MEDIATED SIGNALING PATHWAY
REGULATION OF B CELL RECEPTOR SIGNALING PATHWAY
LYMPHOCYTE ACTIVATION INVOLVED IN IMMUNE RESPONSE
CELL ACTIVATION INVOLVED IN IMMUNE RESPONSE
LEUKOCYTE ACTIVATION INVOLVED IN IMMUNE RESPONSE
ALPHA−BETA T CELL ACTIVATION
T CELL SELECTION
REGULATION OF INTERFERON−GAMMA PRODUCTION
NEGATIVE REGULATION OF CELL GROWTH
hsa−miR−216b−5p
HALLMARK_EPITHELIAL_MESENCHYMAL_TRANSITION
hsa−miR−135a−5p
HALLMARK_APICAL_JUNCTION
Cell adhesion molecules (CAMs)
Focal adhesion
REGULATION OF EXTENT OF CELL GROWTH
REGULATION OF AXONOGENESIS
POSITIVE REGULATION OF AXONOGENESIS
ACTION POTENTIAL
CELLULAR TRANSITION METAL ION HOMEOSTASIS
NEGATIVE REGULATION OF CANONICAL WNT SIGNALING PATHWAY
NEGATIVE REGULATION OF WNT SIGNALING PATHWAY
POSITIVE REGULATION OF CELL GROWTH
REGULATION OF CELL SHAPE
WATER HOMEOSTASIS
MONOVALENT INORGANIC CATION HOMEOSTASIS
Systemic lupus erythematosus

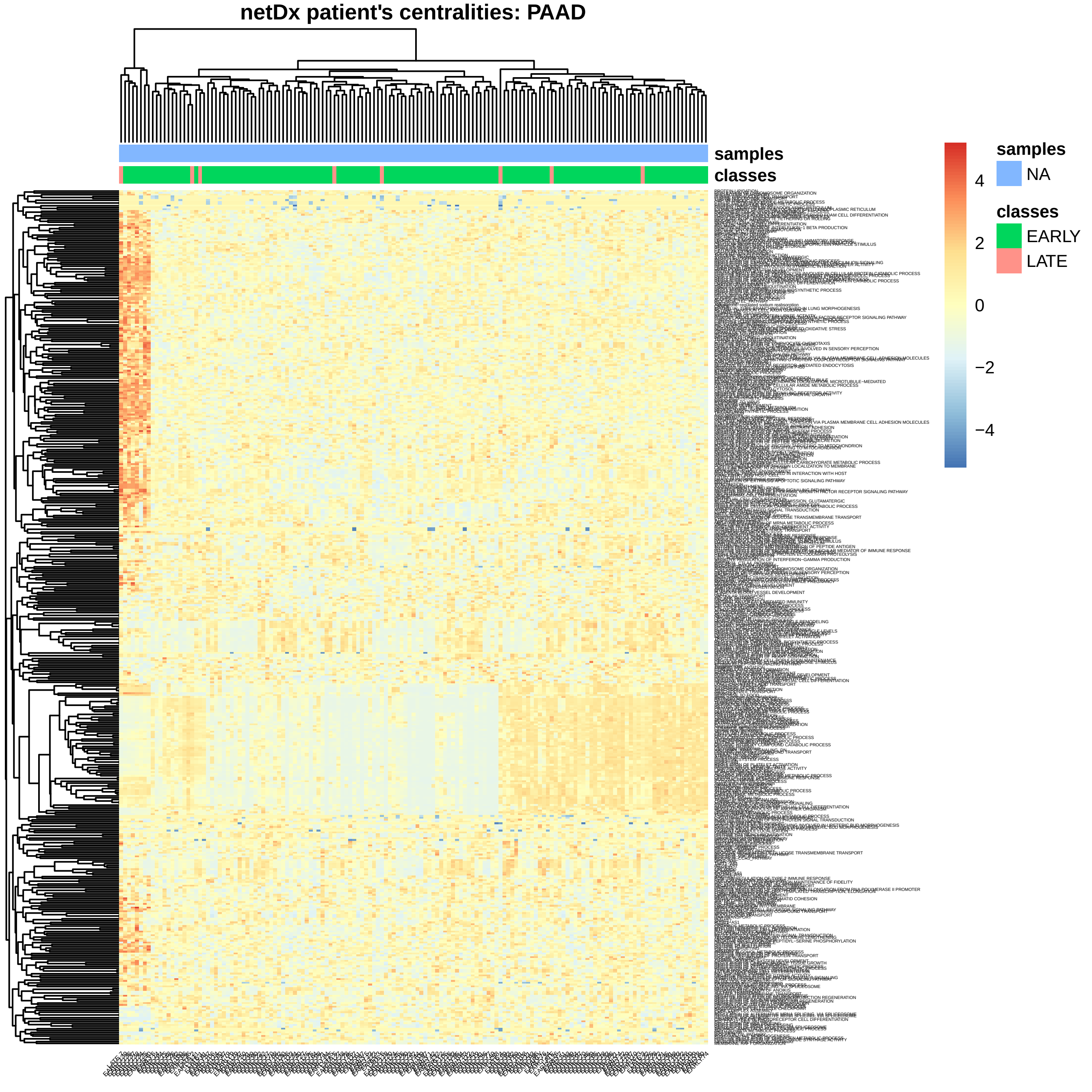
netDx patient's centralities: PAAD
samples
classes
samples
NA
classes
EARLY
LATE
4
2
0
−2
−4

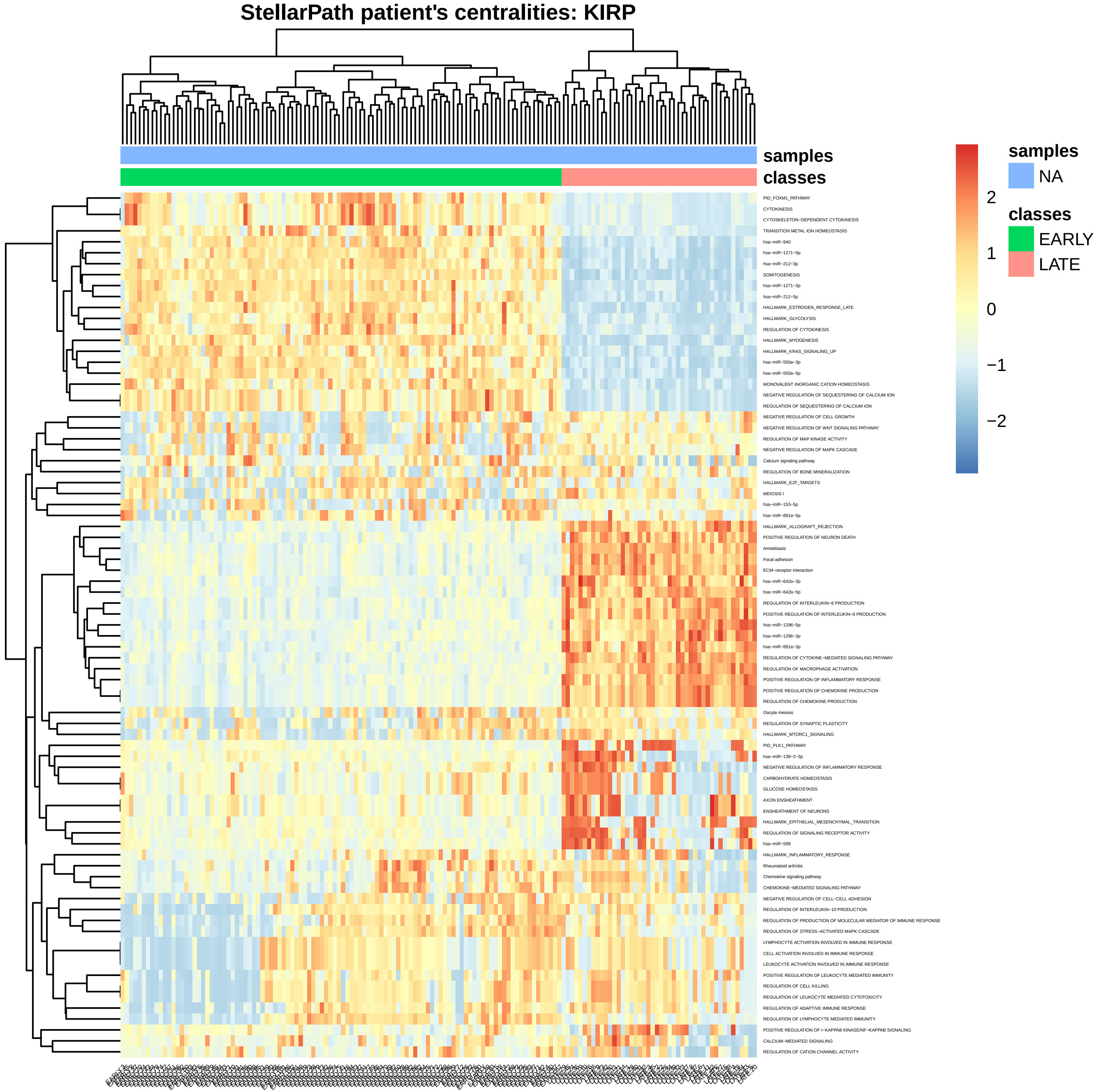

StellarPath patient's centralities: KIRP
samples
classes
samples
NA
classes
EARLY
LATE
2
1
0
−1
−2
PID_FOXM1_PATHWAY
CYTOKINESIS
CYTOSKELETON−DEPENDENT CYTOKINESIS
TRANSITION METAL ION HOMEOSTASIS
hsa−miR−940
hsa−miR−1271−5p
hsa−miR−212−3p
SOMITOGENESIS
hsa−miR−1271−3p
hsa−miR−212−5p
HALLMARK_ESTROGEN_RESPONSE_LATE
HALLMARK_GLYCOLYSIS
REGULATION OF CYTOKINESIS
HALLMARK_MYOGENESIS
HALLMARK_KRAS_SIGNALING_UP
hsa−miR−550a−3p
hsa−miR−550a−5p
MONOVALENT INORGANIC CATION HOMEOSTASIS
NEGATIVE REGULATION OF SEQUESTERING OF CALCIUM ION
REGULATION OF SEQUESTERING OF CALCIUM ION
NEGATIVE REGULATION OF CELL GROWTH
NEGATIVE REGULATION OF WNT SIGNALING PATHWAY
REGULATION OF MAP KINASE ACTIVITY
NEGATIVE REGULATION OF MAPK CASCADE
Calcium signaling pathway
REGULATION OF BONE MINERALIZATION
HALLMARK_E2F_TARGETS
MEIOSIS I
hsa−miR−153−5p
hsa−miR−891a−5p
HALLMARK_ALLOGRAFT_REJECTION
POSITIVE REGULATION OF NEURON DEATH
Amoebiasis
Focal adhesion
ECM−receptor interaction
hsa−miR−642a−3p
hsa−miR−642a−5p
REGULATION OF INTERLEUKIN−6 PRODUCTION
POSITIVE REGULATION OF INTERLEUKIN−6 PRODUCTION
hsa−miR−1296−5p
hsa−miR−1296−3p
hsa−miR−891a−3p
REGULATION OF CYTOKINE−MEDIATED SIGNALING PATHWAY
REGULATION OF MACROPHAGE ACTIVATION
POSITIVE REGULATION OF INFLAMMATORY RESPONSE
POSITIVE REGULATION OF CHEMOKINE PRODUCTION
REGULATION OF CHEMOKINE PRODUCTION
Oocyte meiosis
REGULATION OF SYNAPTIC PLASTICITY
HALLMARK_MTORC1_SIGNALING
PID_PLK1_PATHWAY
hsa−miR−138−2−3p
NEGATIVE REGULATION OF INFLAMMATORY RESPONSE
CARBOHYDRATE HOMEOSTASIS
GLUCOSE HOMEOSTASIS
AXON ENSHEATHMENT
ENSHEATHMENT OF NEURONS
HALLMARK_EPITHELIAL_MESENCHYMAL_TRANSITION
REGULATION OF SIGNALING RECEPTOR ACTIVITY
hsa−miR−599
HALLMARK_INFLAMMATORY_RESPONSE
Rheumatoid arthritis
Chemokine signaling pathway
CHEMOKINE−MEDIATED SIGNALING PATHWAY
NEGATIVE REGULATION OF CELL−CELL ADHESION
REGULATION OF INTERLEUKIN−10 PRODUCTION
REGULATION OF PRODUCTION OF MOLECULAR MEDIATOR OF IMMUNE RESPONSE
REGULATION OF STRESS−ACTIVATED MAPK CASCADE
LYMPHOCYTE ACTIVATION INVOLVED IN IMMUNE RESPONSE
CELL ACTIVATION INVOLVED IN IMMUNE RESPONSE
LEUKOCYTE ACTIVATION INVOLVED IN IMMUNE RESPONSE
POSITIVE REGULATION OF LEUKOCYTE MEDIATED IMMUNITY
REGULATION OF CELL KILLING
REGULATION OF LEUKOCYTE MEDIATED CYTOTOXICITY
REGULATION OF ADAPTIVE IMMUNE RESPONSE
REGULATION OF LYMPHOCYTE MEDIATED IMMUNITY
POSITIVE REGULATION OF I−KAPPAB KINASE/NF−KAPPAB SIGNALING
CALCIUM−MEDIATED SIGNALING
REGULATION OF CATION CHANNEL ACTIVITY

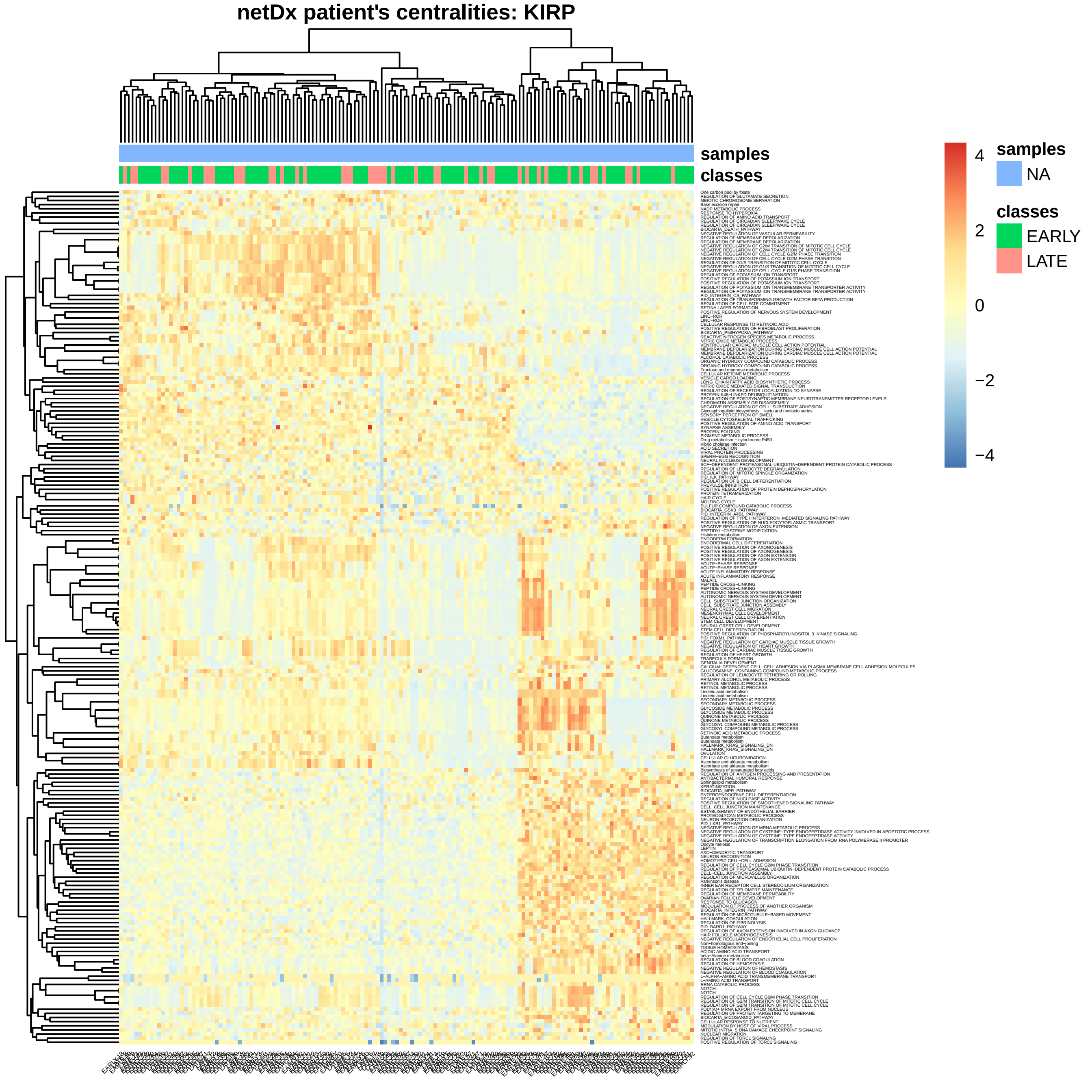
netDx patient's centralities: KIRP
samples
classes
4
2
0
−2
−4
samples
NA
classes
EARLY
LATE

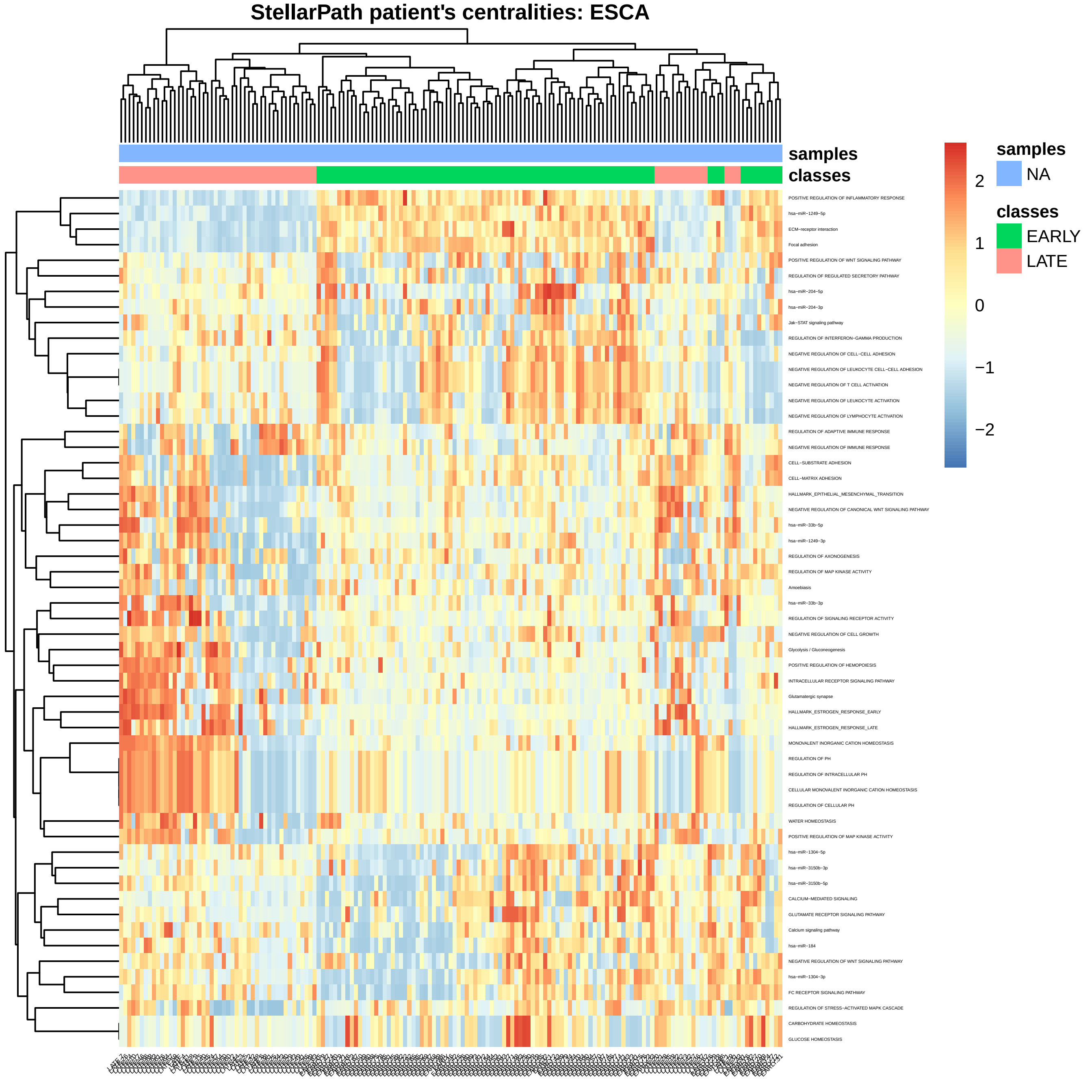
StellarPath patient's centralities: ESCA
samples
classes
samples
NA
classes
EARLY
LATE
2
1
0
−1
−2
POSITIVE REGULATION OF INFLAMMATORY RESPONSE
hsa−miR−1249−5p
ECM−receptor interaction
Focal adhesion
POSITIVE REGULATION OF WNT SIGNALING PATHWAY
REGULATION OF REGULATED SECRETORY PATHWAY
hsa−miR−204−5p
hsa−miR−204−3p
Jak−STAT signaling pathway
REGULATION OF INTERFERON−GAMMA PRODUCTION
NEGATIVE REGULATION OF CELL−CELL ADHESION
NEGATIVE REGULATION OF LEUKOCYTE CELL−CELL ADHESION
NEGATIVE REGULATION OF T CELL ACTIVATION
NEGATIVE REGULATION OF LEUKOCYTE ACTIVATION
NEGATIVE REGULATION OF LYMPHOCYTE ACTIVATION
REGULATION OF ADAPTIVE IMMUNE RESPONSE
NEGATIVE REGULATION OF IMMUNE RESPONSE
CELL−SUBSTRATE ADHESION
CELL−MATRIX ADHESION
HALLMARK_EPITHELIAL_MESENCHYMAL_TRANSITION
NEGATIVE REGULATION OF CANONICAL WNT SIGNALING PATHWAY
hsa−miR−33b−5p
hsa−miR−1249−3p
REGULATION OF AXONOGENESIS
REGULATION OF MAP KINASE ACTIVITY
Amoebiasis
hsa−miR−33b−3p
REGULATION OF SIGNALING RECEPTOR ACTIVITY
NEGATIVE REGULATION OF CELL GROWTH
Glycolysis / Gluconeogenesis
POSITIVE REGULATION OF HEMOPOIESIS
INTRACELLULAR RECEPTOR SIGNALING PATHWAY
Glutamatergic synapse
HALLMARK_ESTROGEN_RESPONSE_EARLY
HALLMARK_ESTROGEN_RESPONSE_LATE
MONOVALENT INORGANIC CATION HOMEOSTASIS
REGULATION OF PH
REGULATION OF INTRACELLULAR PH
CELLULAR MONOVALENT INORGANIC CATION HOMEOSTASIS
REGULATION OF CELLULAR PH
WATER HOMEOSTASIS
POSITIVE REGULATION OF MAP KINASE ACTIVITY
hsa−miR−1304−5p
hsa−miR−3150b−3p
hsa−miR−3150b−5p
CALCIUM−MEDIATED SIGNALING
GLUTAMATE RECEPTOR SIGNALING PATHWAY
Calcium signaling pathway
hsa−miR−184
NEGATIVE REGULATION OF WNT SIGNALING PATHWAY
hsa−miR−1304−3p
FC RECEPTOR SIGNALING PATHWAY
REGULATION OF STRESS−ACTIVATED MAPK CASCADE
CARBOHYDRATE HOMEOSTASIS
GLUCOSE HOMEOSTASIS

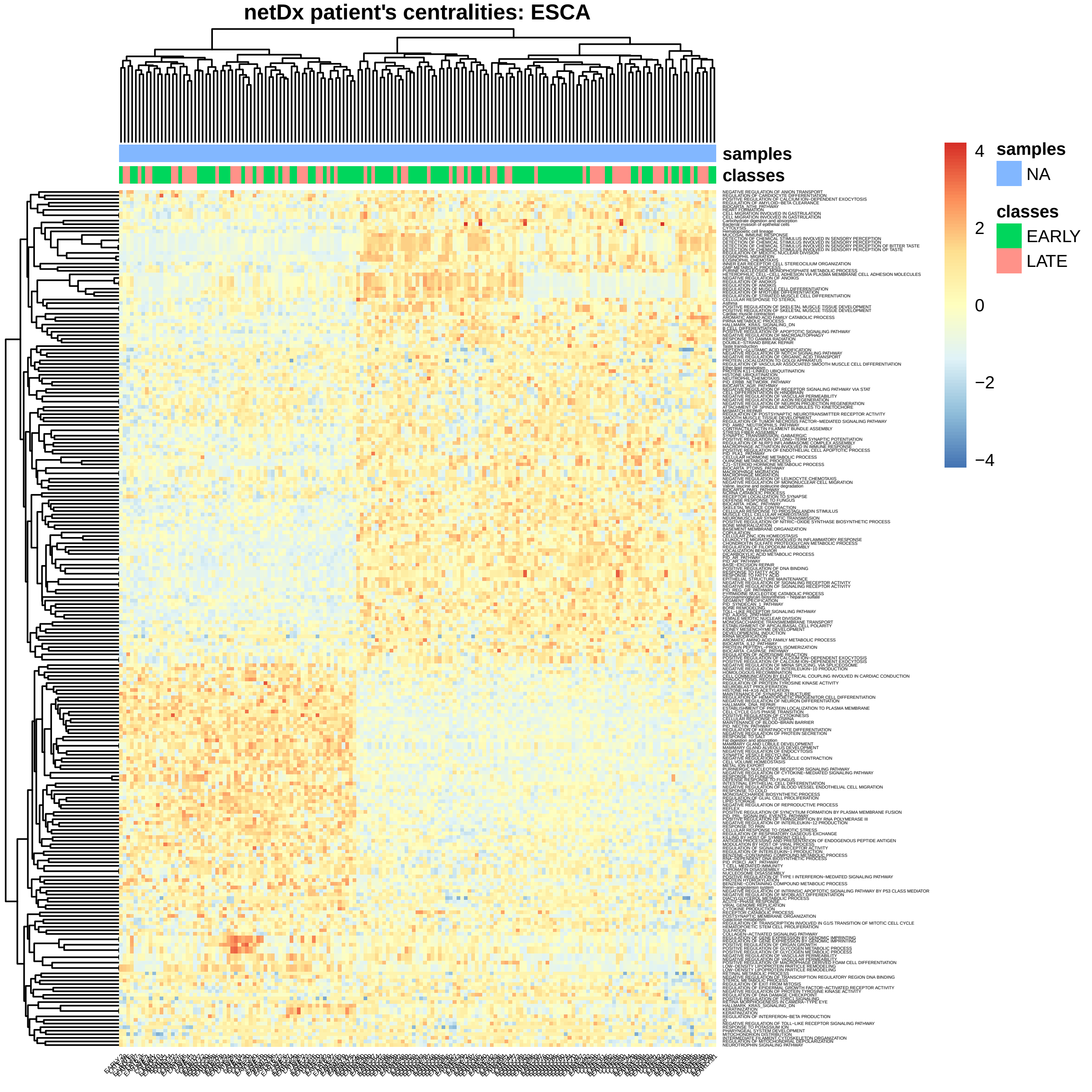
netDx patient's centralities: ESCA
samples
classes
4
2
0
−2
−4
samples
NA
classes
EARLY
LATE

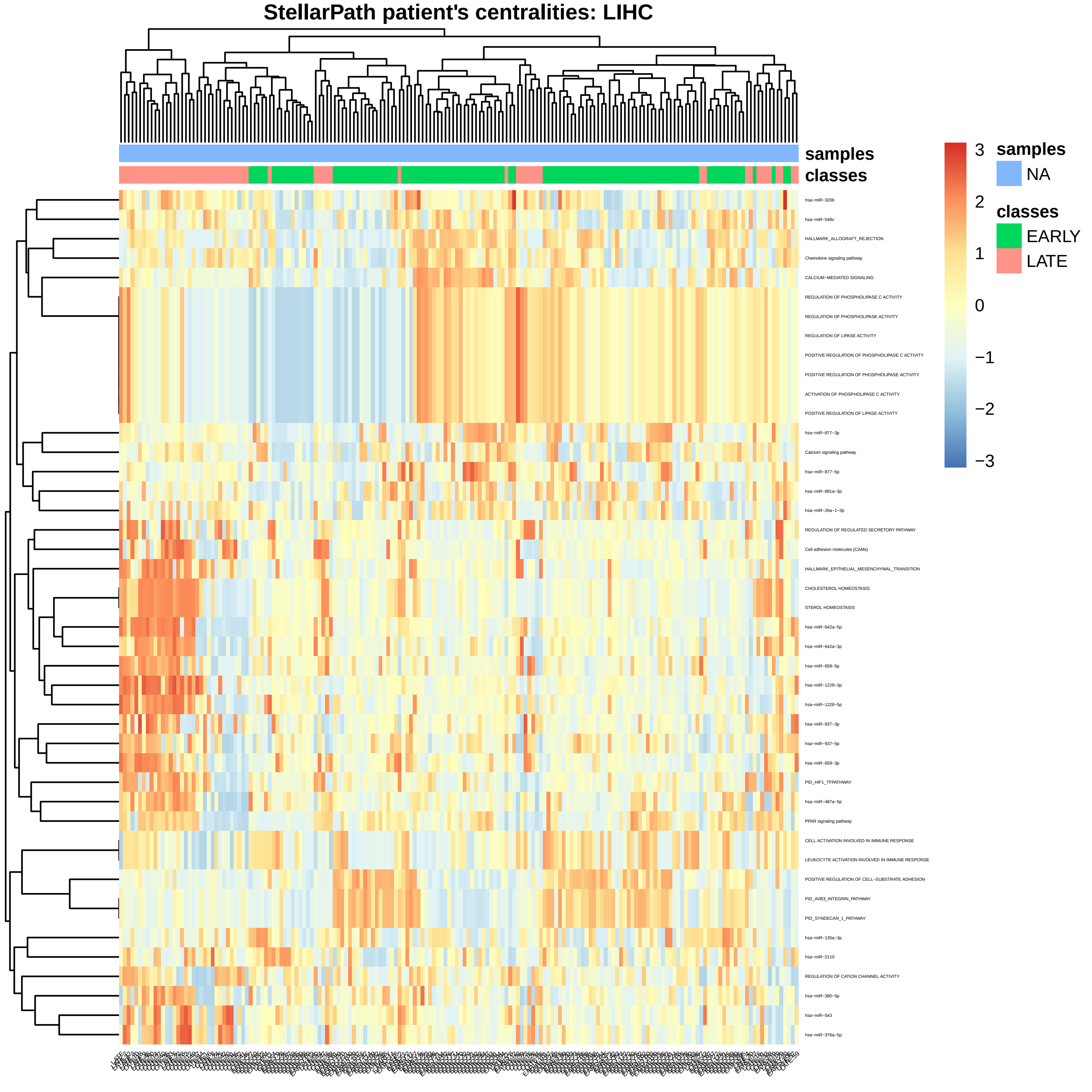
StellarPath patient's centralities: LIHC
samples
classes
samples
NA
classes
EARLY
LATE
3
2
1
0
−1
−2
−3
hsa−miR−320b
hsa−miR−548v
HALLMARK_ALLOGRAFT_REJECTION
Chemokine signaling pathway
CALCIUM−MEDIATED SIGNALING
REGULATION OF PHOSPHOLIPASE C ACTIVITY
REGULATION OF PHOSPHOLIPASE ACTIVITY
REGULATION OF LIPASE ACTIVITY
POSITIVE REGULATION OF PHOSPHOLIPASE C ACTIVITY
POSITIVE REGULATION OF PHOSPHOLIPASE ACTIVITY
ACTIVATION OF PHOSPHOLIPASE C ACTIVITY
POSITIVE REGULATION OF LIPASE ACTIVITY
hsa−miR−877−3p
Calcium signaling pathway
hsa−miR−877−5p
hsa−miR−891a−3p
hsa−miR−26a−1−3p
REGULATION OF REGULATED SECRETORY PATHWAY
Cell adhesion molecules (CAMs)
HALLMARK_EPITHELIAL_MESENCHYMAL_TRANSITION
CHOLESTEROL HOMEOSTASIS
STEROL HOMEOSTASIS
hsa−miR−642a−5p
hsa−miR−642a−3p
hsa−miR−659−5p
hsa−miR−1228−3p
hsa−miR−1228−5p
hsa−miR−937−3p
hsa−miR−937−5p
hsa−miR−659−3p
PID_HIF1_TFPATHWAY
hsa−miR−487a−5p
PPAR signaling pathway
CELL ACTIVATION INVOLVED IN IMMUNE RESPONSE
LEUKOCYTE ACTIVATION INVOLVED IN IMMUNE RESPONSE
POSITIVE REGULATION OF CELL−SUBSTRATE ADHESION
PID_AVB3_INTEGRIN_PATHWAY
PID_SYNDECAN_1_PATHWAY
hsa−miR−135a−3p
hsa−miR−2110
REGULATION OF CATION CHANNEL ACTIVITY
hsa−miR−380−5p
hsa−miR−543
hsa−miR−376a−5p

netDx patient's centralities: LIHC

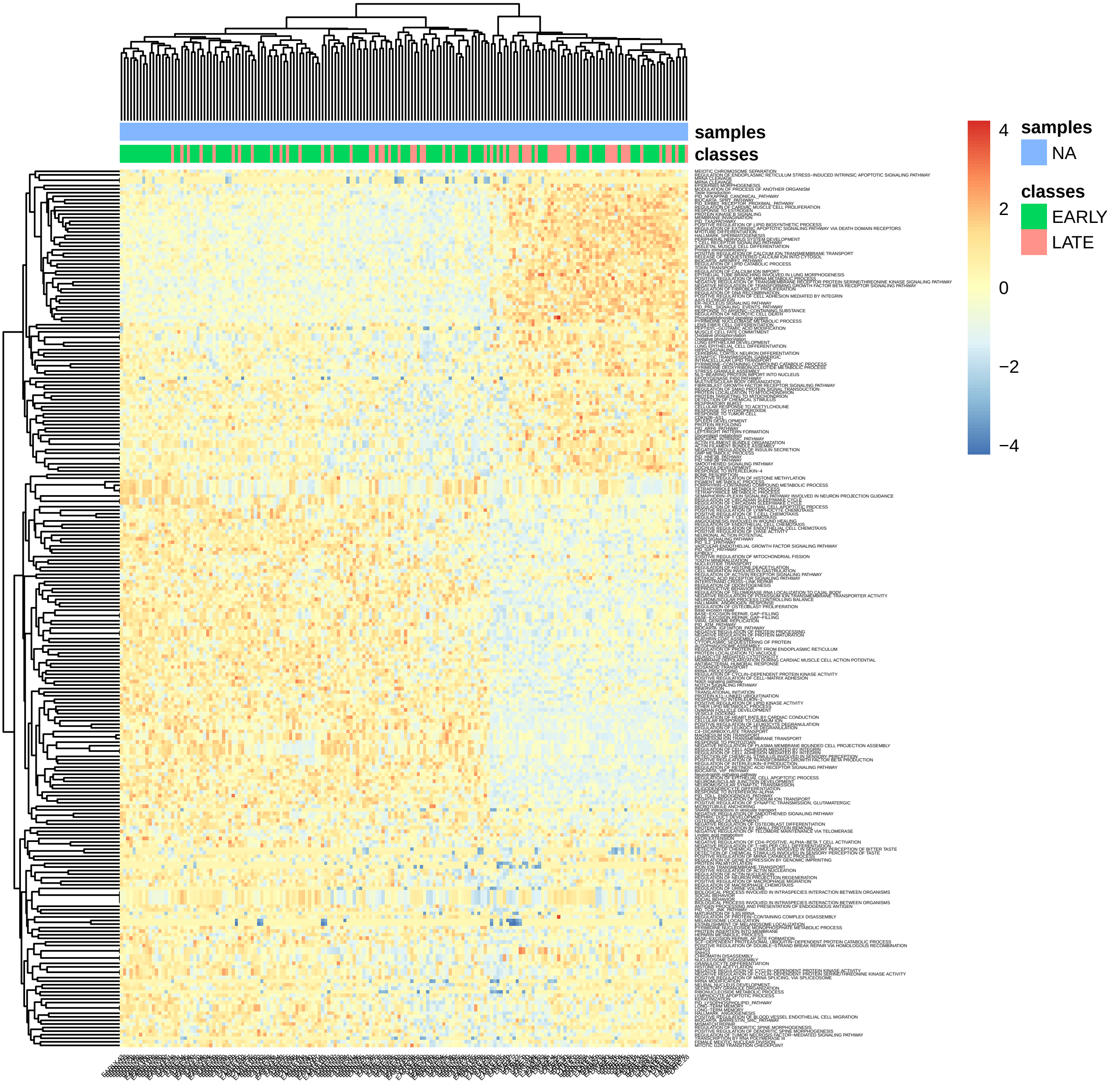

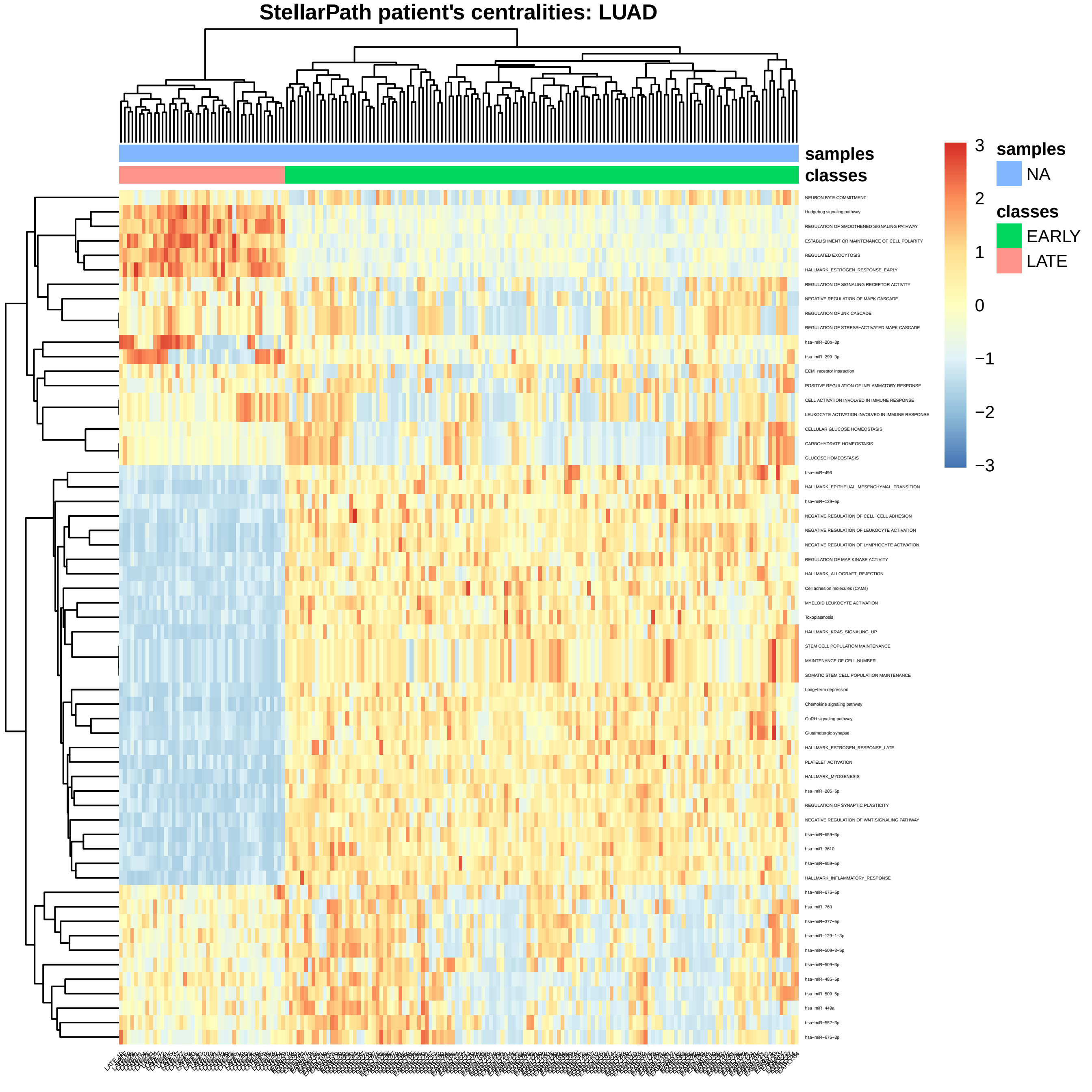
StellarPath patient's centralities: LUAD
samples
classes
NEURON FATE COMMITMENT
Hedgehog signaling pathway
REGULATION OF SMOOTHENED SIGNALING PATHWAY
ESTABLISHMENT OR MAINTENANCE OF CELL POLARITY
REGULATED EXOCYTOSIS
HALLMARK_ESTROGEN_RESPONSE_EARLY
REGULATION OF SIGNALING RECEPTOR ACTIVITY
NEGATIVE REGULATION OF MAPK CASCADE
REGULATION OF JNK CASCADE
REGULATION OF STRESS−ACTIVATED MAPK CASCADE
hsa−miR−20b−3p
hsa−miR−299−3p
ECM−receptor interaction
POSITIVE REGULATION OF INFLAMMATORY RESPONSE
CELL ACTIVATION INVOLVED IN IMMUNE RESPONSE
LEUKOCYTE ACTIVATION INVOLVED IN IMMUNE RESPONSE
CELLULAR GLUCOSE HOMEOSTASIS
CARBOHYDRATE HOMEOSTASIS
GLUCOSE HOMEOSTASIS
hsa−miR−496
HALLMARK_EPITHELIAL_MESENCHYMAL_TRANSITION
hsa−miR−129−5p
NEGATIVE REGULATION OF CELL−CELL ADHESION
NEGATIVE REGULATION OF LEUKOCYTE ACTIVATION
NEGATIVE REGULATION OF LYMPHOCYTE ACTIVATION
REGULATION OF MAP KINASE ACTIVITY
HALLMARK_ALLOGRAFT_REJECTION
Cell adhesion molecules (CAMs)
MYELOID LEUKOCYTE ACTIVATION
Toxoplasmosis
HALLMARK_KRAS_SIGNALING_UP
STEM CELL POPULATION MAINTENANCE
MAINTENANCE OF CELL NUMBER
SOMATIC STEM CELL POPULATION MAINTENANCE
Long−term depression
Chemokine signaling pathway
GnRH signaling pathway
Glutamatergic synapse
HALLMARK_ESTROGEN_RESPONSE_LATE
PLATELET ACTIVATION
HALLMARK_MYOGENESIS
hsa−miR−205−5p
REGULATION OF SYNAPTIC PLASTICITY
NEGATIVE REGULATION OF WNT SIGNALING PATHWAY
hsa−miR−659−3p
hsa−miR−3610
hsa−miR−659−5p
HALLMARK_INFLAMMATORY_RESPONSE
hsa−miR−675−5p
hsa−miR−760
hsa−miR−377−5p
hsa−miR−129−1−3p
hsa−miR−509−3−5p
hsa−miR−509−3p
hsa−miR−485−5p
hsa−miR−509−5p
hsa−miR−449a
hsa−miR−552−3p
hsa−miR−675−3p
3
2
1
0
−1
−2
−3
samples
NA
classes
EARLY
LATE

netDx patient's centralities: LUAD

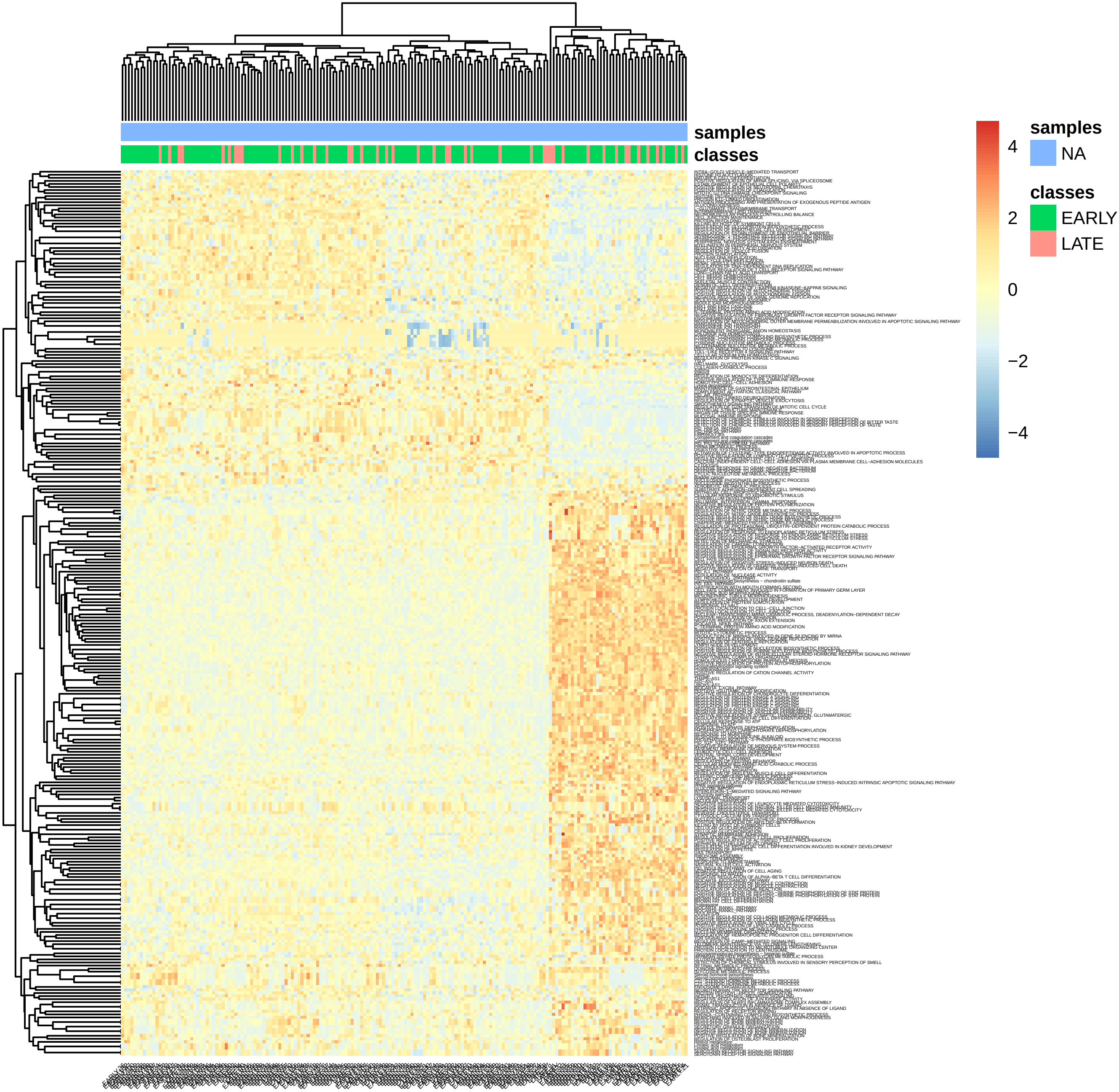

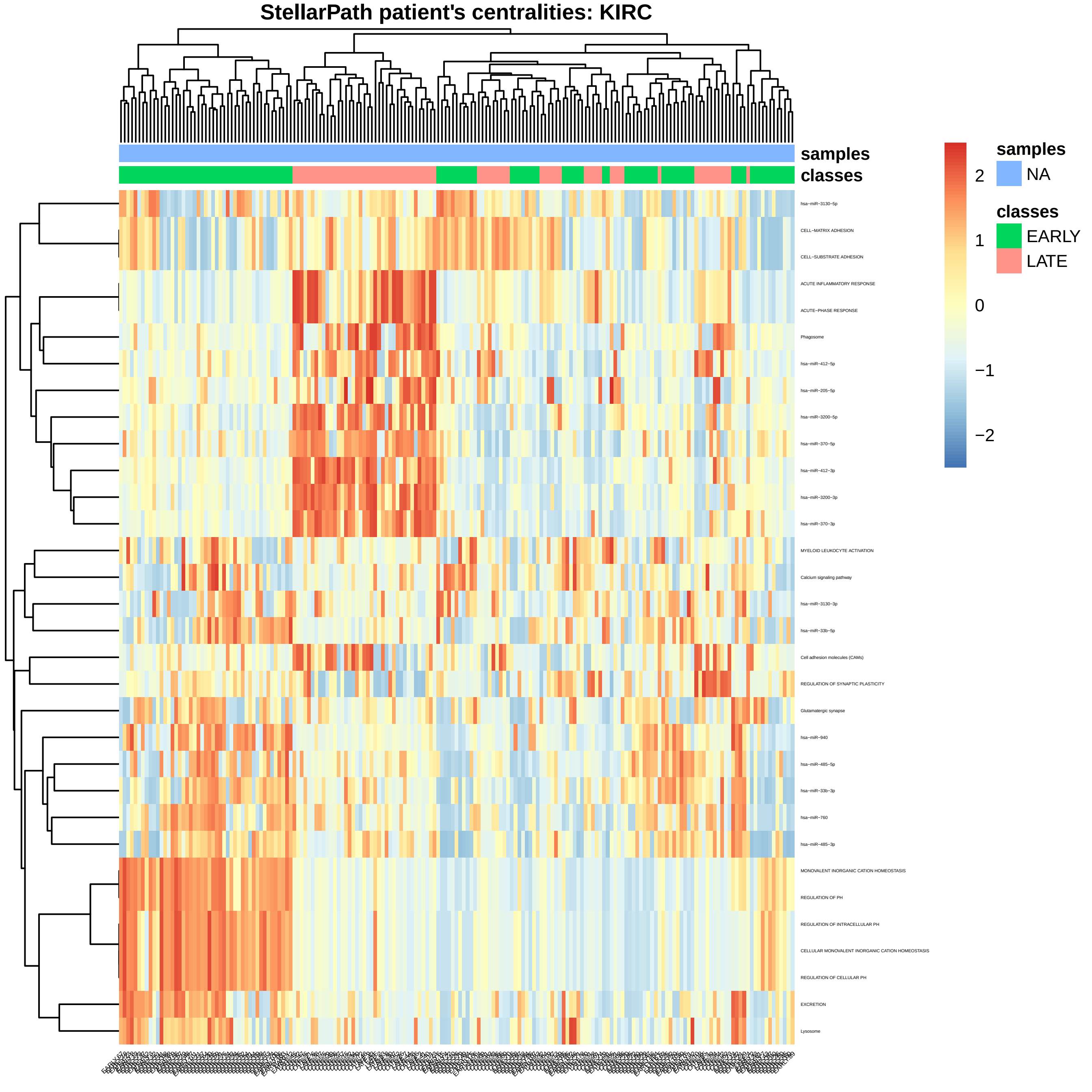
StellarPath patient's centralities: KIRC
samples
classes
samples
NA
classes
EARLY
LATE
2
1
0
−1
−2
hsa−miR−3130−5p
CELL−MATRIX ADHESION
CELL−SUBSTRATE ADHESION
ACUTE INFLAMMATORY RESPONSE
ACUTE−PHASE RESPONSE
Phagosome
hsa−miR−412−5p
hsa−miR−205−5p
hsa−miR−3200−5p
hsa−miR−370−5p
hsa−miR−412−3p
hsa−miR−3200−3p
hsa−miR−370−3p
MYELOID LEUKOCYTE ACTIVATION
Calcium signaling pathway
hsa−miR−3130−3p
hsa−miR−33b−5p
Cell adhesion molecules (CAMs)
REGULATION OF SYNAPTIC PLASTICITY
Glutamatergic synapse
hsa−miR−940
hsa−miR−485−5p
hsa−miR−33b−3p
hsa−miR−760
hsa−miR−485−3p
MONOVALENT INORGANIC CATION HOMEOSTASIS
REGULATION OF PH
REGULATION OF INTRACELLULAR PH
CELLULAR MONOVALENT INORGANIC CATION HOMEOSTASIS
REGULATION OF CELLULAR PH
EXCRETION
Lysosome

netDx patient's centralities: KIRC

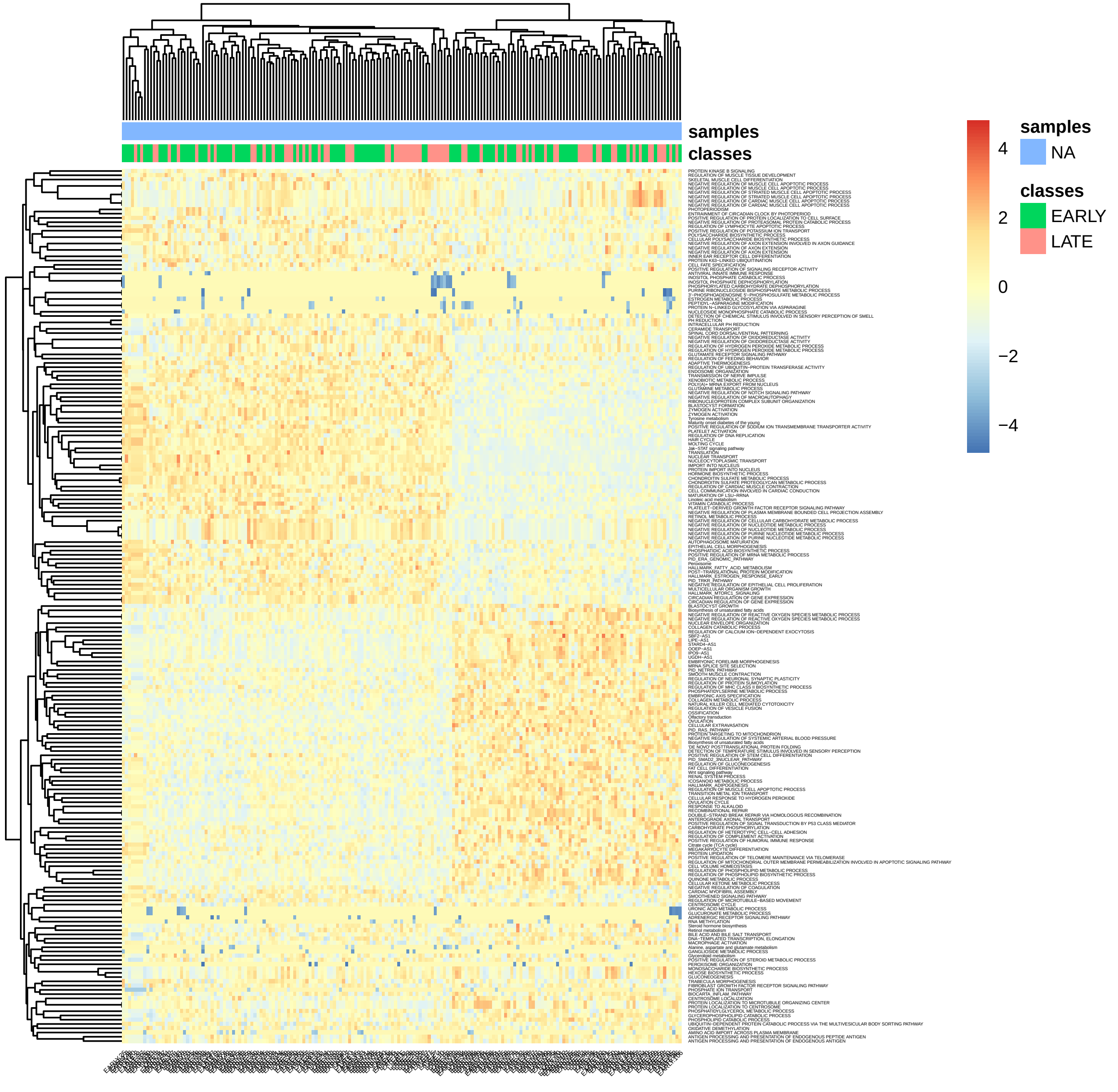

StellarPath patient's centralities: HNSC

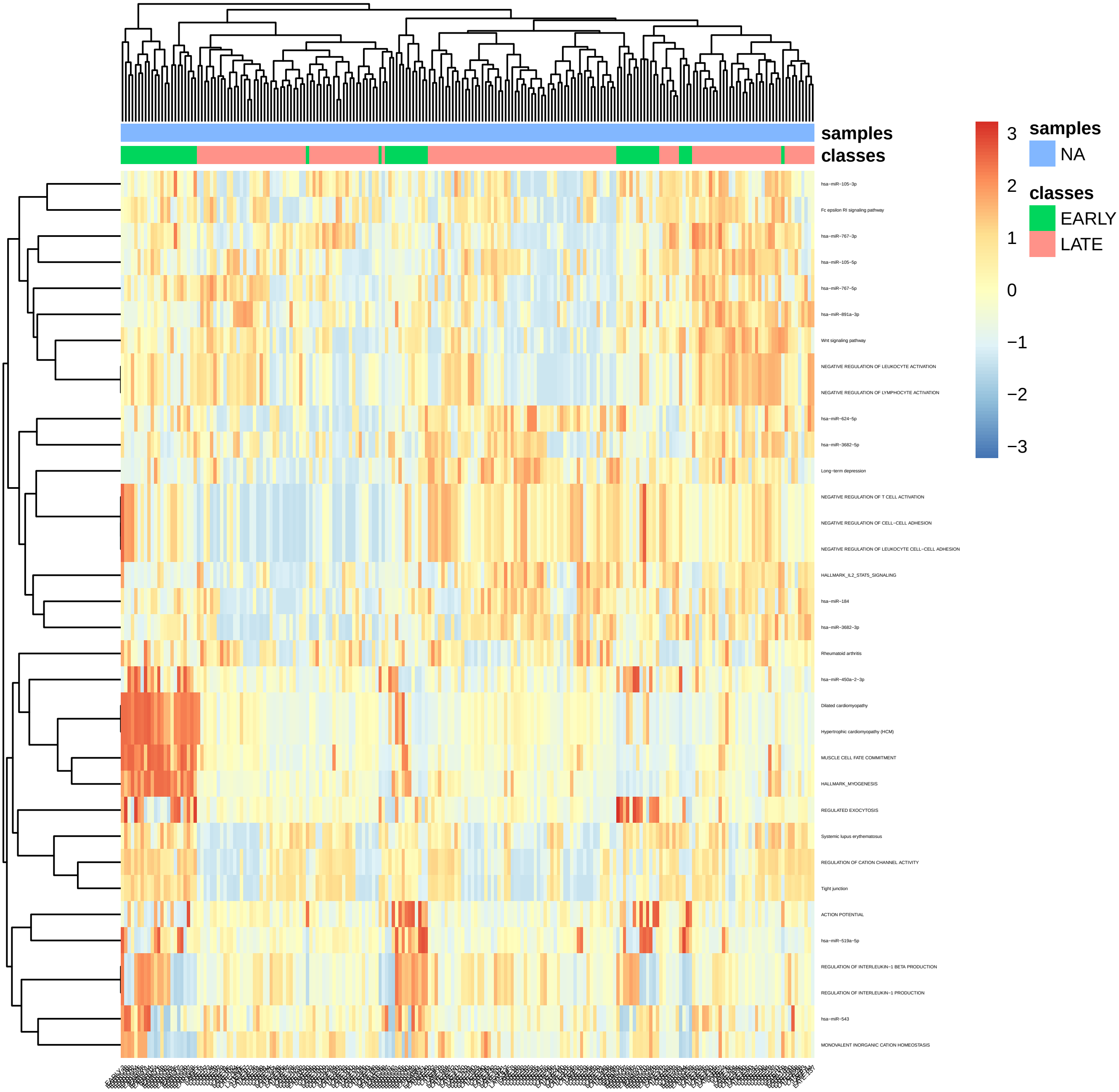

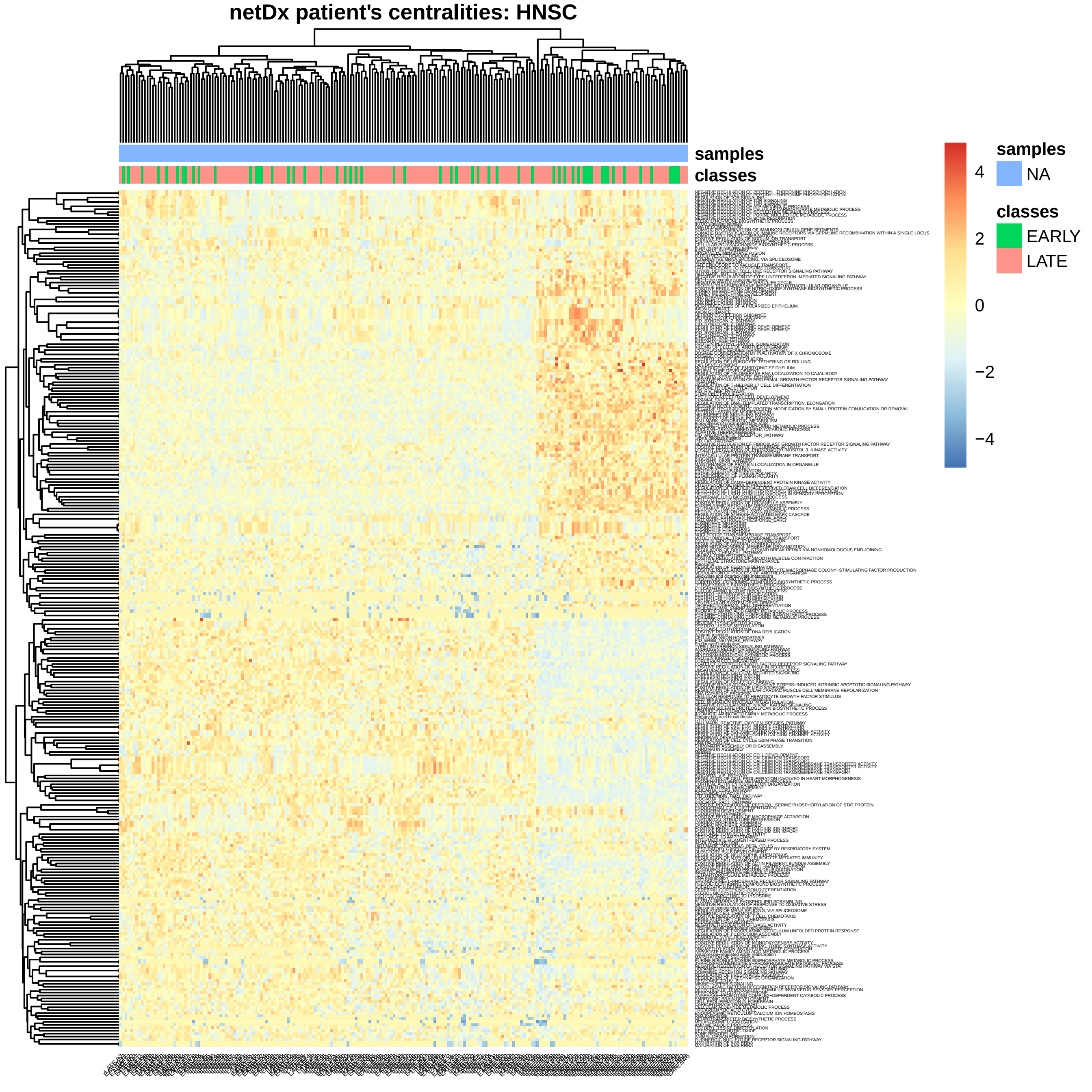
netDx patient's centralities: HNSC
samples
classes
4
2
0
−2
−4
samples
NA
classes
EARLY
LATE

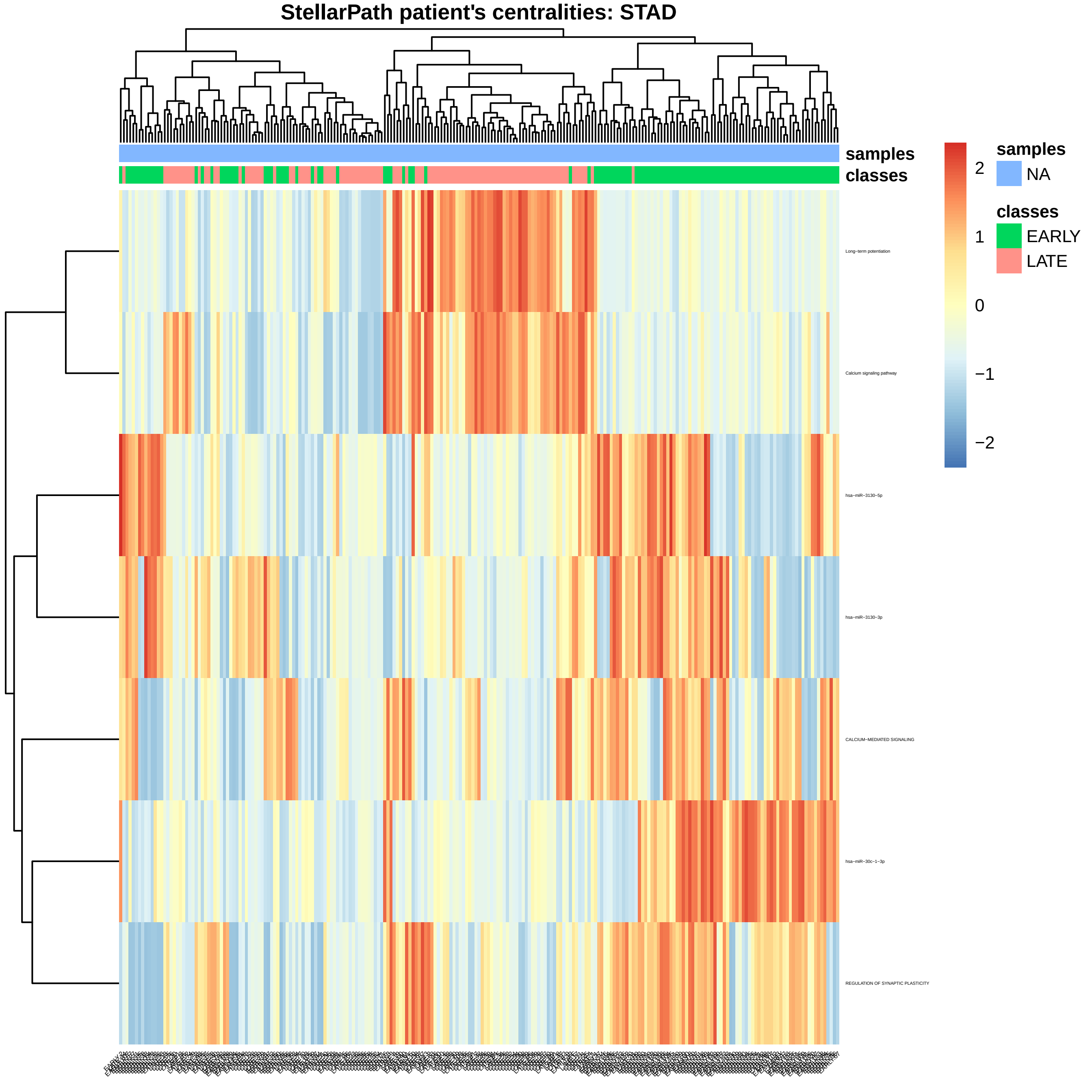
StellarPath patient's centralities: STAD
samples
classes
Long−term potentiation
Calcium signaling pathway
hsa−miR−3130−5p
hsa−miR−3130−3p
CALCIUM−MEDIATED SIGNALING
hsa−miR−30c−1−3p
REGULATION OF SYNAPTIC PLASTICITY
2
1
0
−1
−2
samples
NA
classes
EARLY
LATE

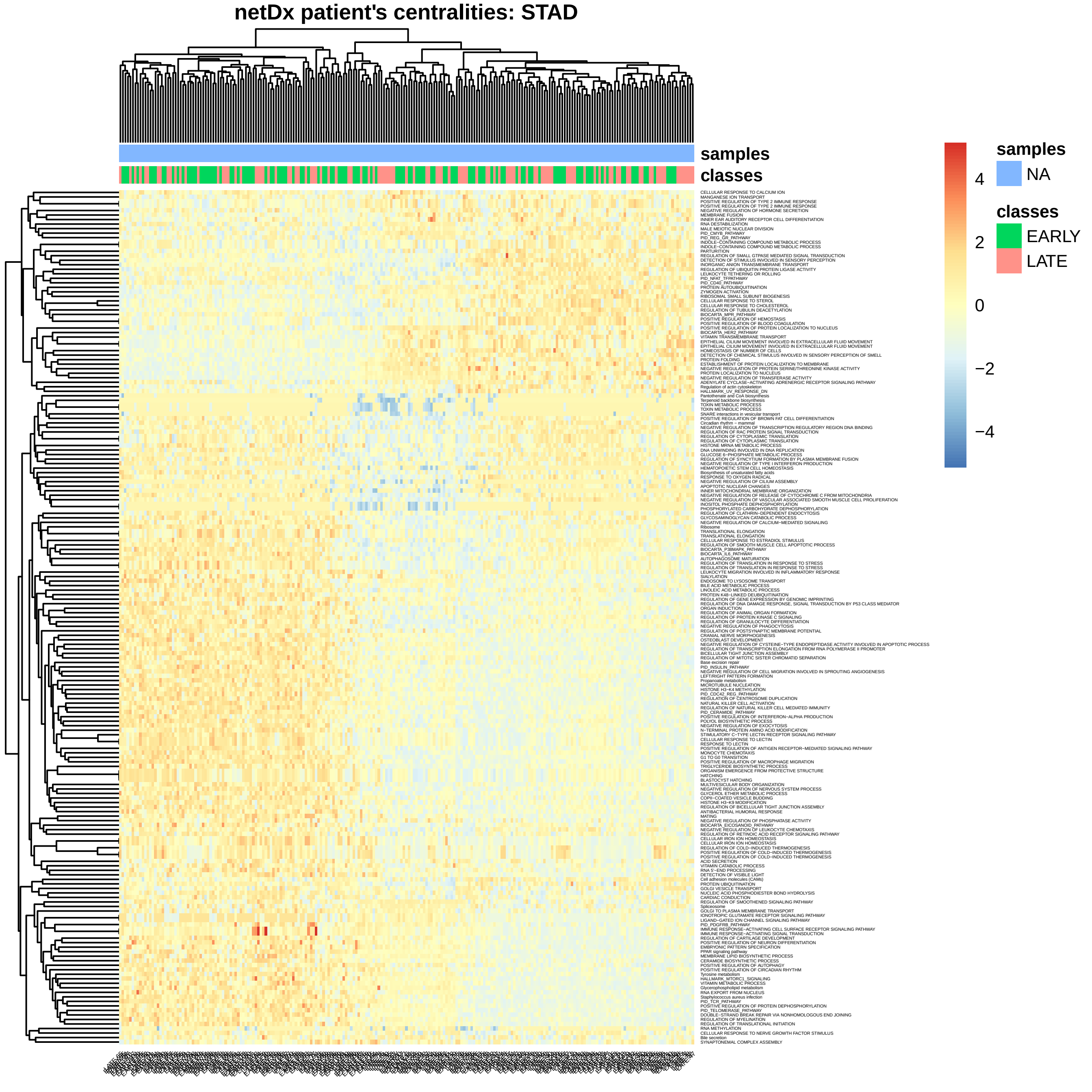
netDx patient's centralities: STAD
samples
classes
samples
NA
classes
EARLY
LATE
4
2
0
−2
−4
CELLULAR RESPONSE TO CALCIUM ION
MANGANESE ION TRANSPORT
POSITIVE REGULATION OF TYPE 2 IMMUNE RESPONSE
POSITIVE REGULATION OF TYPE 2 IMMUNE RESPONSE
NEGATIVE REGULATION OF HORMONE SECRETION
MEMBRANE FUSION
INNER EAR AUDITORY RECEPTOR CELL DIFFERENTIATION
RNA DESTABILIZATION
MALE MEIOTIC NUCLEAR DIVISION
PID_CMYB_PATHWAY
PID_REG_GR_PATHWAY
INDOLE−CONTAINING COMPOUND METABOLIC PROCESS
INDOLE−CONTAINING COMPOUND METABOLIC PROCESS
PARTURITION
REGULATION OF SMALL GTPASE MEDIATED SIGNAL TRANSDUCTION
DETECTION OF STIMULUS INVOLVED IN SENSORY PERCEPTION
INORGANIC ANION TRANSMEMBRANE TRANSPORT
REGULATION OF UBIQUITIN PROTEIN LIGASE ACTIVITY
LEUKOCYTE TETHERING OR ROLLING
PID_NFAT_TFPATHWAY
PID_CD40_PATHWAY
PROTEIN AUTOUBIQUITINATION
ZYMOGEN ACTIVATION
RIBOSOMAL SMALL SUBUNIT BIOGENESIS
CELLULAR RESPONSE TO STEROL
CELLULAR RESPONSE TO CHOLESTEROL
REGULATION OF TUBULIN DEACETYLATION
BIOCARTA_MPR_PATHWAY
POSITIVE REGULATION OF HEMOSTASIS
POSITIVE REGULATION OF BLOOD COAGULATION
POSITIVE REGULATION OF PROTEIN LOCALIZATION TO NUCLEUS
BIOCARTA_HER2_PATHWAY
VITAMIN TRANSMEMBRANE TRANSPORT
EPITHELIAL CILIUM MOVEMENT INVOLVED IN EXTRACELLULAR FLUID MOVEMENT
EPITHELIAL CILIUM MOVEMENT INVOLVED IN EXTRACELLULAR FLUID MOVEMENT
HOMEOSTASIS OF NUMBER OF CELLS
DETECTION OF CHEMICAL STIMULUS INVOLVED IN SENSORY PERCEPTION OF SMELL
PROTEIN FOLDING
ESTABLISHMENT OF PROTEIN LOCALIZATION TO MEMBRANE
NEGATIVE REGULATION OF PROTEIN SERINE/THREONINE KINASE ACTIVITY
PROTEIN LOCALIZATION TO NUCLEUS
NEGATIVE REGULATION OF TRANSFERASE ACTIVITY
ADENYLATE CYCLASE−ACTIVATING ADRENERGIC RECEPTOR SIGNALING PATHWAY
Regulation of actin cytoskeleton
HALLMARK_UV_RESPONSE_DN
Pantothenate and CoA biosynthesis
Terpenoid backbone biosynthesis
TOXIN METABOLIC PROCESS
TOXIN METABOLIC PROCESS
SNARE interactions in vesicular transport
POSITIVE REGULATION OF BROWN FAT CELL DIFFERENTIATION
Circadian rhythm − mammal
NEGATIVE REGULATION OF TRANSCRIPTION REGULATORY REGION DNA BINDING
REGULATION OF RAC PROTEIN SIGNAL TRANSDUCTION
REGULATION OF CYTOPLASMIC TRANSLATION
REGULATION OF CYTOPLASMIC TRANSLATION
HISTONE MRNA METABOLIC PROCESS
DNA UNWINDING INVOLVED IN DNA REPLICATION
GLUCOSE 6−PHOSPHATE METABOLIC PROCESS
REGULATION OF SYNCYTIUM FORMATION BY PLASMA MEMBRANE FUSION
NEGATIVE REGULATION OF TYPE I INTERFERON PRODUCTION
HEMATOPOIETIC STEM CELL HOMEOSTASIS
Biosynthesis of unsaturated fatty acids
RESPONSE TO OXYGEN RADICAL
NEGATIVE REGULATION OF CILIUM ASSEMBLY
APOPTOTIC NUCLEAR CHANGES
INNER MITOCHONDRIAL MEMBRANE ORGANIZATION
NEGATIVE REGULATION OF RELEASE OF CYTOCHROME C FROM MITOCHONDRIA
NEGATIVE REGULATION OF VASCULAR ASSOCIATED SMOOTH MUSCLE CELL PROLIFERATION
INOSITOL PHOSPHATE DEPHOSPHORYLATION
PHOSPHORYLATED CARBOHYDRATE DEPHOSPHORYLATION
REGULATION OF CLATHRIN−DEPENDENT ENDOCYTOSIS
GLYCOSAMINOGLYCAN CATABOLIC PROCESS
NEGATIVE REGULATION OF CALCIUM−MEDIATED SIGNALING
Ribosome
TRANSLATIONAL ELONGATION
TRANSLATIONAL ELONGATION
CELLULAR RESPONSE TO ESTRADIOL STIMULUS
REGULATION OF SMOOTH MUSCLE CELL APOPTOTIC PROCESS
BIOCARTA_P38MAPK_PATHWAY
BIOCARTA_IL6_PATHWAY
AUTOPHAGOSOME MATURATION
REGULATION OF TRANSLATION IN RESPONSE TO STRESS
REGULATION OF TRANSLATION IN RESPONSE TO STRESS
LEUKOCYTE MIGRATION INVOLVED IN INFLAMMATORY RESPONSE
SIALYLATION
ENDOSOME TO LYSOSOME TRANSPORT
BILE ACID METABOLIC PROCESS
LINOLEIC ACID METABOLIC PROCESS
PROTEIN K48−LINKED DEUBIQUITINATION
REGULATION OF GENE EXPRESSION BY GENOMIC IMPRINTING
REGULATION OF DNA DAMAGE RESPONSE, SIGNAL TRANSDUCTION BY P53 CLASS MEDIATOR
ORGAN INDUCTION
REGULATION OF ANIMAL ORGAN FORMATION
REGULATION OF PROTEIN KINASE C SIGNALING
REGULATION OF GRANULOCYTE DIFFERENTIATION
NEGATIVE REGULATION OF PHAGOCYTOSIS
REGULATION OF POSTSYNAPTIC MEMBRANE POTENTIAL
CRANIAL NERVE MORPHOGENESIS
OSTEOBLAST DEVELOPMENT
NEGATIVE REGULATION OF CYSTEINE−TYPE ENDOPEPTIDASE ACTIVITY INVOLVED IN APOPTOTIC PROCESS
REGULATION OF TRANSCRIPTION ELONGATION FROM RNA POLYMERASE II PROMOTER
BICELLULAR TIGHT JUNCTION ASSEMBLY
REGULATION OF MITOTIC SISTER CHROMATID SEPARATION
Base excision repair
PID_INSULIN_PATHWAY
NEGATIVE REGULATION OF CELL MIGRATION INVOLVED IN SPROUTING ANGIOGENESIS
LEFT/RIGHT PATTERN FORMATION
Propanoate metabolism
MICROTUBULE NUCLEATION
HISTONE H3−K4 METHYLATION
PID_CDC42_REG_PATHWAY
REGULATION OF CENTROSOME DUPLICATION
NATURAL KILLER CELL ACTIVATION
REGULATION OF NATURAL KILLER CELL MEDIATED IMMUNITY
PID_CERAMIDE_PATHWAY
POSITIVE REGULATION OF INTERFERON−ALPHA PRODUCTION
POLYOL BIOSYNTHETIC PROCESS
NEGATIVE REGULATION OF EXOCYTOSIS
N−TERMINAL PROTEIN AMINO ACID MODIFICATION
STIMULATORY C−TYPE LECTIN RECEPTOR SIGNALING PATHWAY
CELLULAR RESPONSE TO LECTIN
RESPONSE TO LECTIN
POSITIVE REGULATION OF ANTIGEN RECEPTOR−MEDIATED SIGNALING PATHWAY
MONOCYTE CHEMOTAXIS
G1 TO G0 TRANSITION
POSITIVE REGULATION OF MACROPHAGE MIGRATION
TRIGLYCERIDE BIOSYNTHETIC PROCESS
ORGANISM EMERGENCE FROM PROTECTIVE STRUCTURE
HATCHING
BLASTOCYST HATCHING
MULTIVESICULAR BODY ORGANIZATION
NEGATIVE REGULATION OF NERVOUS SYSTEM PROCESS
GLYCEROL ETHER METABOLIC PROCESS
COPII−COATED VESICLE BUDDING
HISTONE H3−K9 MODIFICATION
REGULATION OF BICELLULAR TIGHT JUNCTION ASSEMBLY
ANTIBACTERIAL HUMORAL RESPONSE
MATING
NEGATIVE REGULATION OF PHOSPHATASE ACTIVITY
BIOCARTA_EICOSANOID_PATHWAY
NEGATIVE REGULATION OF LEUKOCYTE CHEMOTAXIS
REGULATION OF RETINOIC ACID RECEPTOR SIGNALING PATHWAY
CELLULAR IRON ION HOMEOSTASIS
CELLULAR IRON ION HOMEOSTASIS
REGULATION OF COLD−INDUCED THERMOGENESIS
POSITIVE REGULATION OF COLD−INDUCED THERMOGENESIS
POSITIVE REGULATION OF COLD−INDUCED THERMOGENESIS
ACID SECRETION
VITAMIN CATABOLIC PROCESS
RNA 5'−END PROCESSING
DETECTION OF VISIBLE LIGHT
Cell adhesion molecules (CAMs)
PROTEIN UBIQUITINATION
GOLGI VESICLE TRANSPORT
NUCLEIC ACID PHOSPHODIESTER BOND HYDROLYSIS
CARDIAC CONDUCTION
REGULATION OF SMOOTHENED SIGNALING PATHWAY
Spliceosome
GOLGI TO PLASMA MEMBRANE TRANSPORT
IONOTROPIC GLUTAMATE RECEPTOR SIGNALING PATHWAY
LIGAND−GATED ION CHANNEL SIGNALING PATHWAY
PID_PDGFRB_PATHWAY
IMMUNE RESPONSE−ACTIVATING CELL SURFACE RECEPTOR SIGNALING PATHWAY
IMMUNE RESPONSE−ACTIVATING SIGNAL TRANSDUCTION
REGULATION OF CARTILAGE DEVELOPMENT
POSITIVE REGULATION OF NEURON DIFFERENTIATION
EMBRYONIC PATTERN SPECIFICATION
PPAR signaling pathway
MEMBRANE LIPID BIOSYNTHETIC PROCESS
CERAMIDE BIOSYNTHETIC PROCESS
POSITIVE REGULATION OF AUTOPHAGY
POSITIVE REGULATION OF CIRCADIAN RHYTHM
Tyrosine metabolism
HALLMARK_MTORC1_SIGNALING
VITAMIN METABOLIC PROCESS
Glycerophospholipid metabolism
RNA EXPORT FROM NUCLEUS
Staphylococcus aureus infection
PID_TCR_PATHWAY
POSITIVE REGULATION OF PROTEIN DEPHOSPHORYLATION
PID_TELOMERASE_PATHWAY
DOUBLE−STRAND BREAK REPAIR VIA NONHOMOLOGOUS END JOINING
REGULATION OF MYELINATION
REGULATION OF TRANSLATIONAL INITIATION
RNA METHYLATION
CELLULAR RESPONSE TO NERVE GROWTH FACTOR STIMULUS
Bile secretion
SYNAPTONEMAL COMPLEX ASSEMBLY

StellarPath patient's centralities: THCA

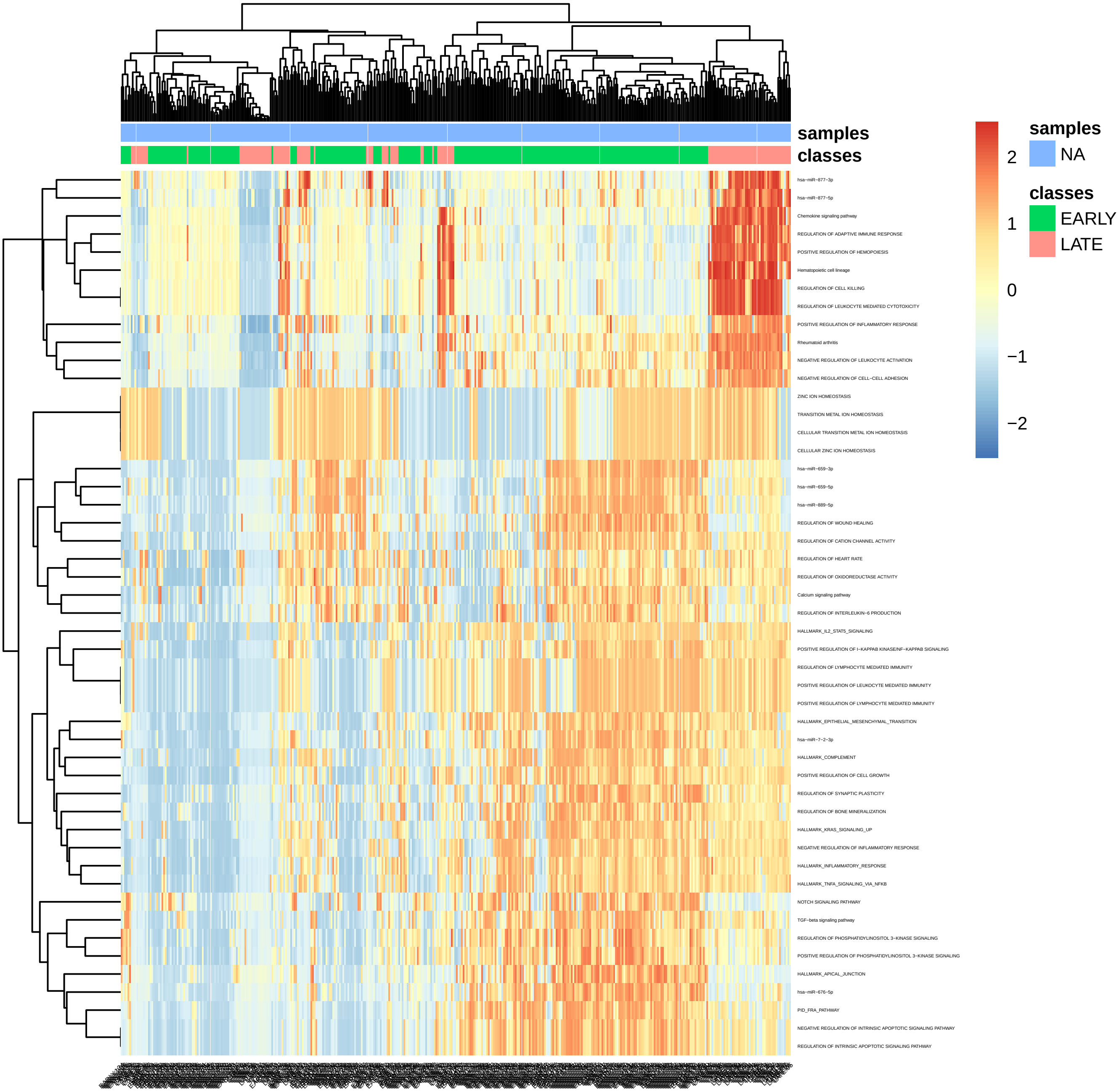

netDx patient's centralities: THCA

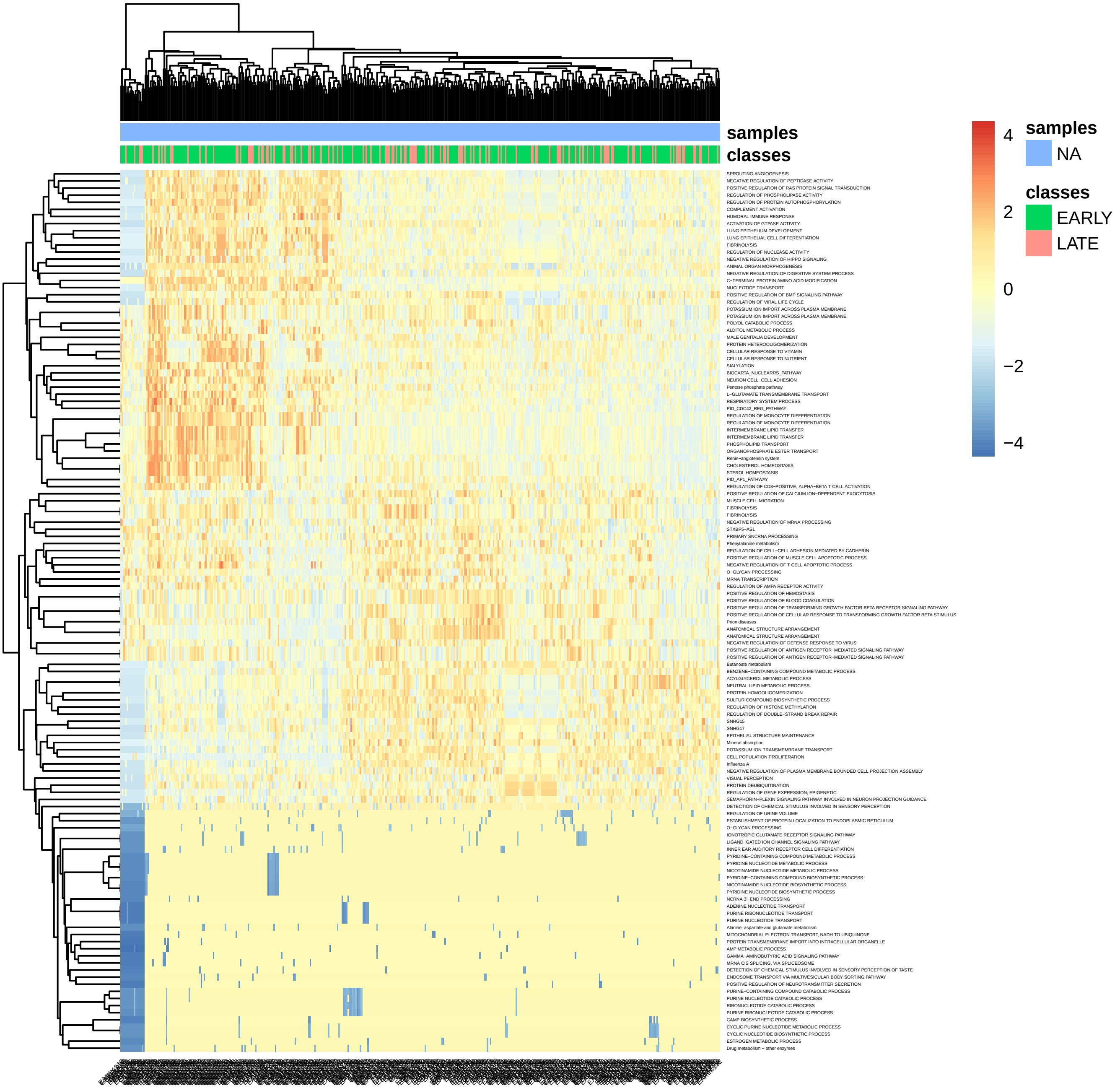

Supplement: S1 File — File of heatmaps. An heatmap is associated to a specific dataset classified by StellarPath. An heatmap represents the patients of a dataset at the columns, the predictive and enriched pathways at the rows, an entry contains the centrality score of a column patient measured in pathway-specific (row) patient similarity network. (PDF) [file pcbi.1012022.s003.pdf]
