# Supplementary material for: StellarPath: Hierarchical-vertical multi-omics classifier synergizes stable markers and interpretable similarity networks for patient profiling
Source: PLoS Comput Biol. 2024 Apr 12;20(4):e1012022. doi: 10.1371/journal.pcbi.1012022 (PMC11042724; doi:10.1371/journal.pcbi.1012022)

Focal adhesion || activated in UM with power 2

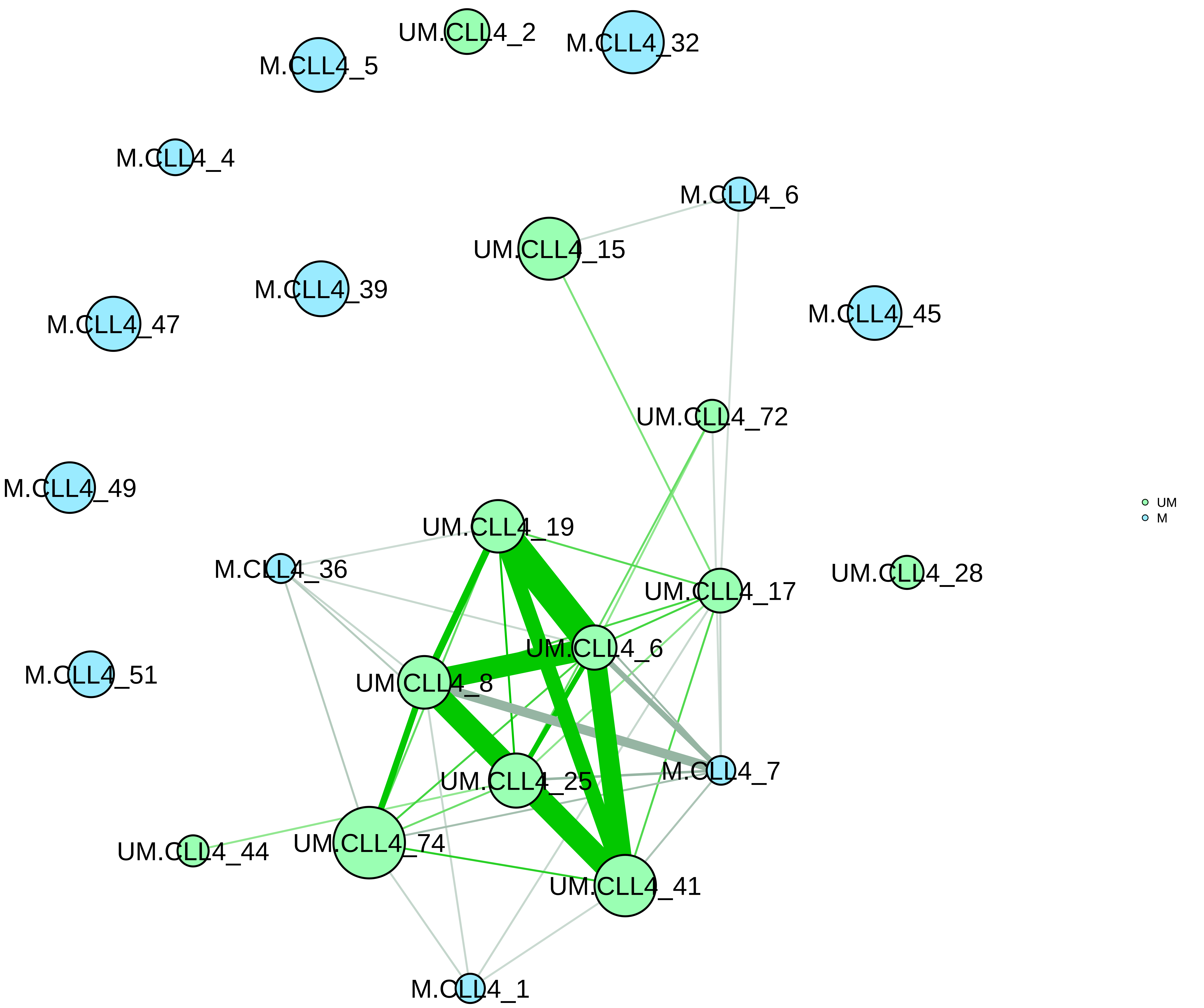

HALLMARK_ALLOGRAFT_REJECTION || activated in UM with power 5

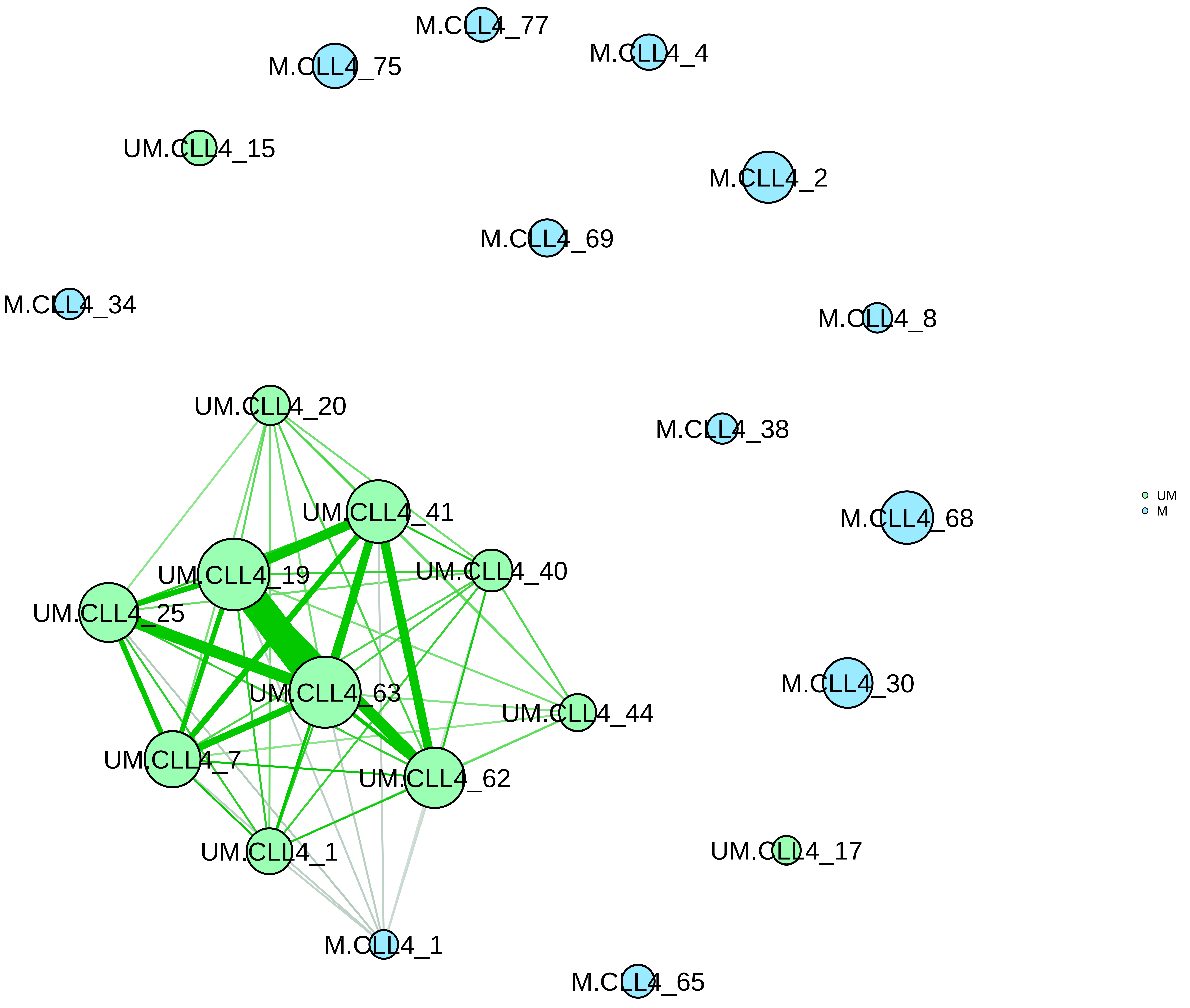

HALLMARK_EPITHELIAL_MESENCHYMAL_TRANSITION || activated in UM with power 3

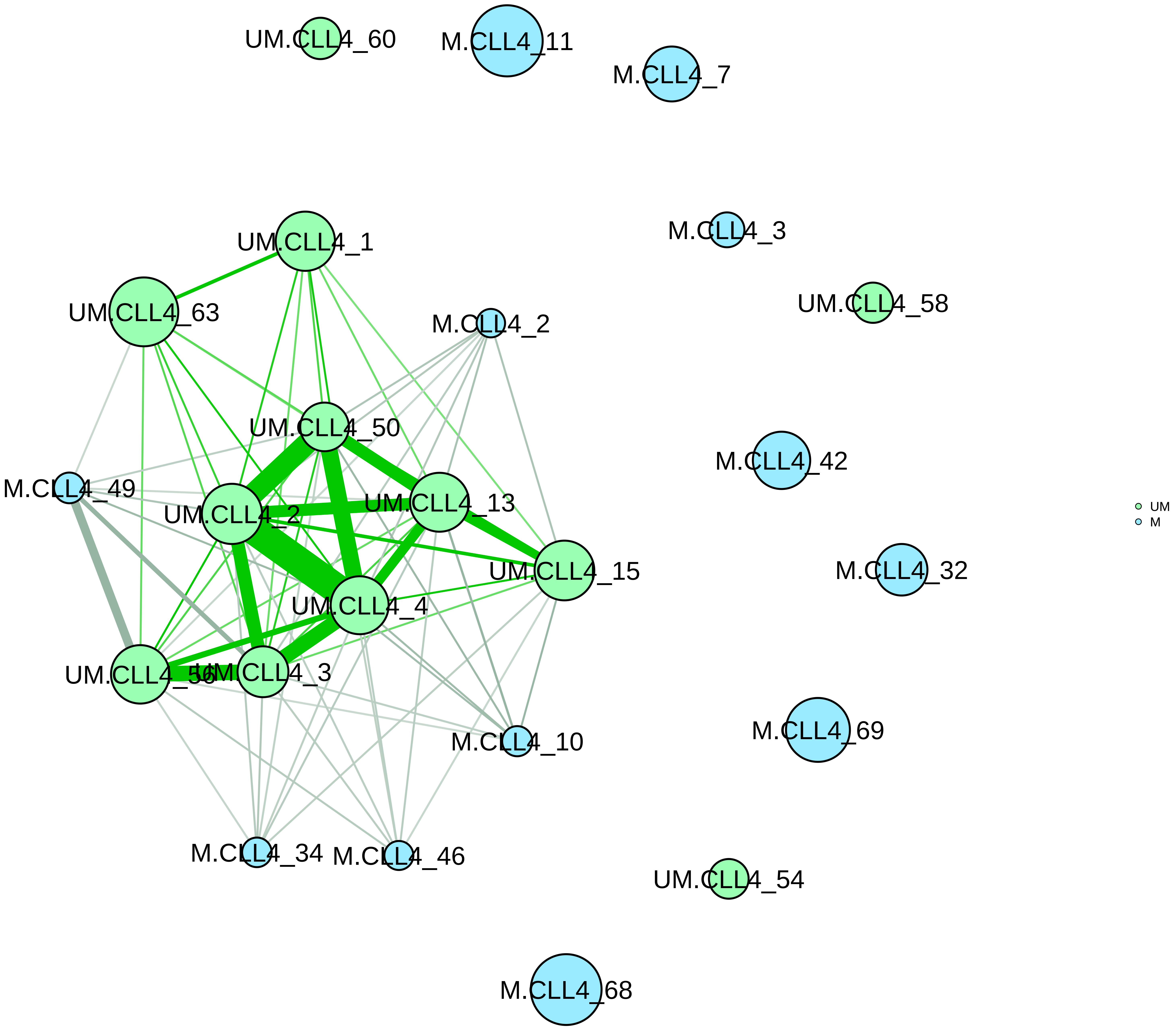

HALLMARK_TNFA_SIGNALING_VIA_NFKB || inhibited in UM with power 1

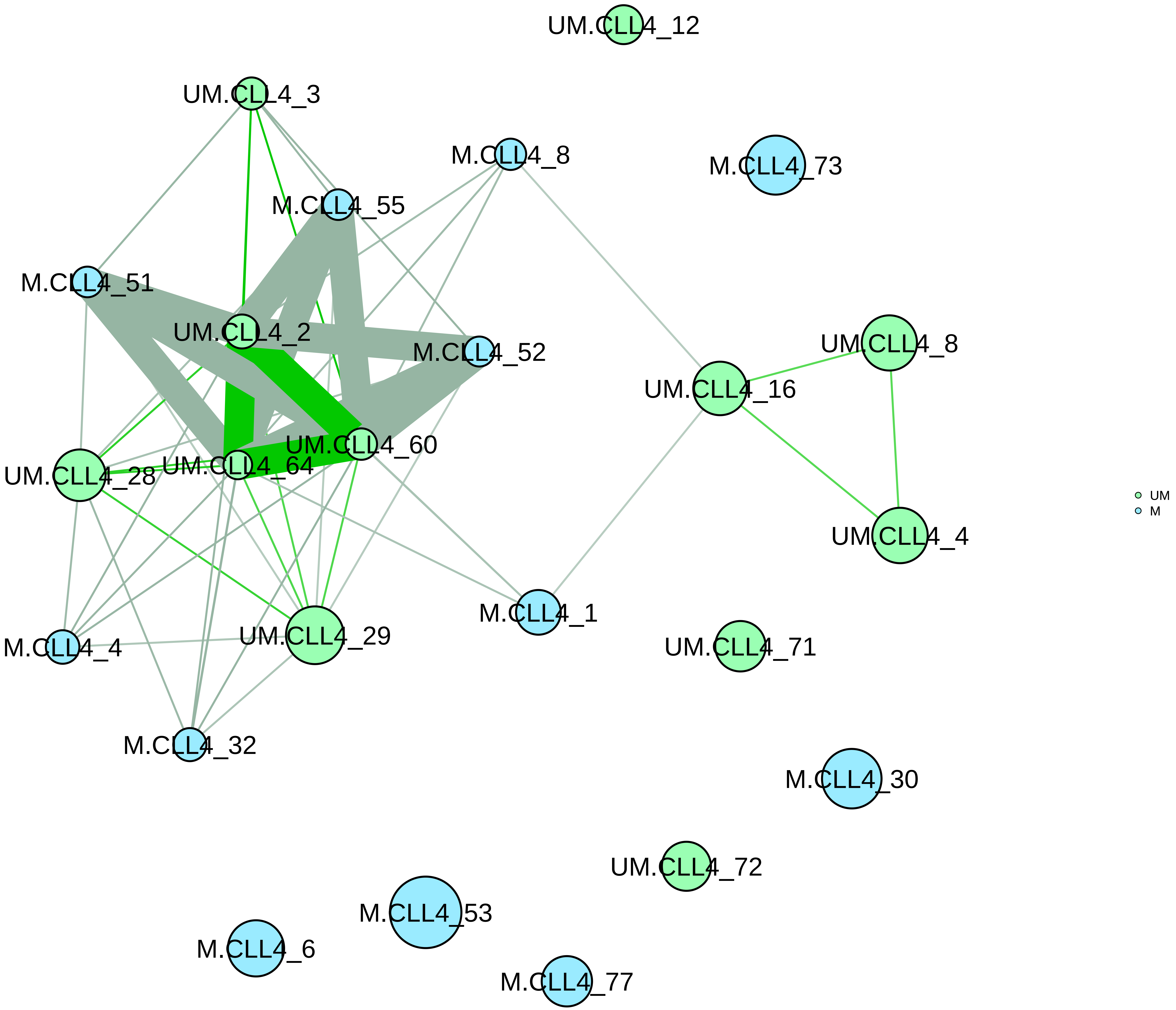

Intestinal immune network for IgA production || activated in UM with power 4

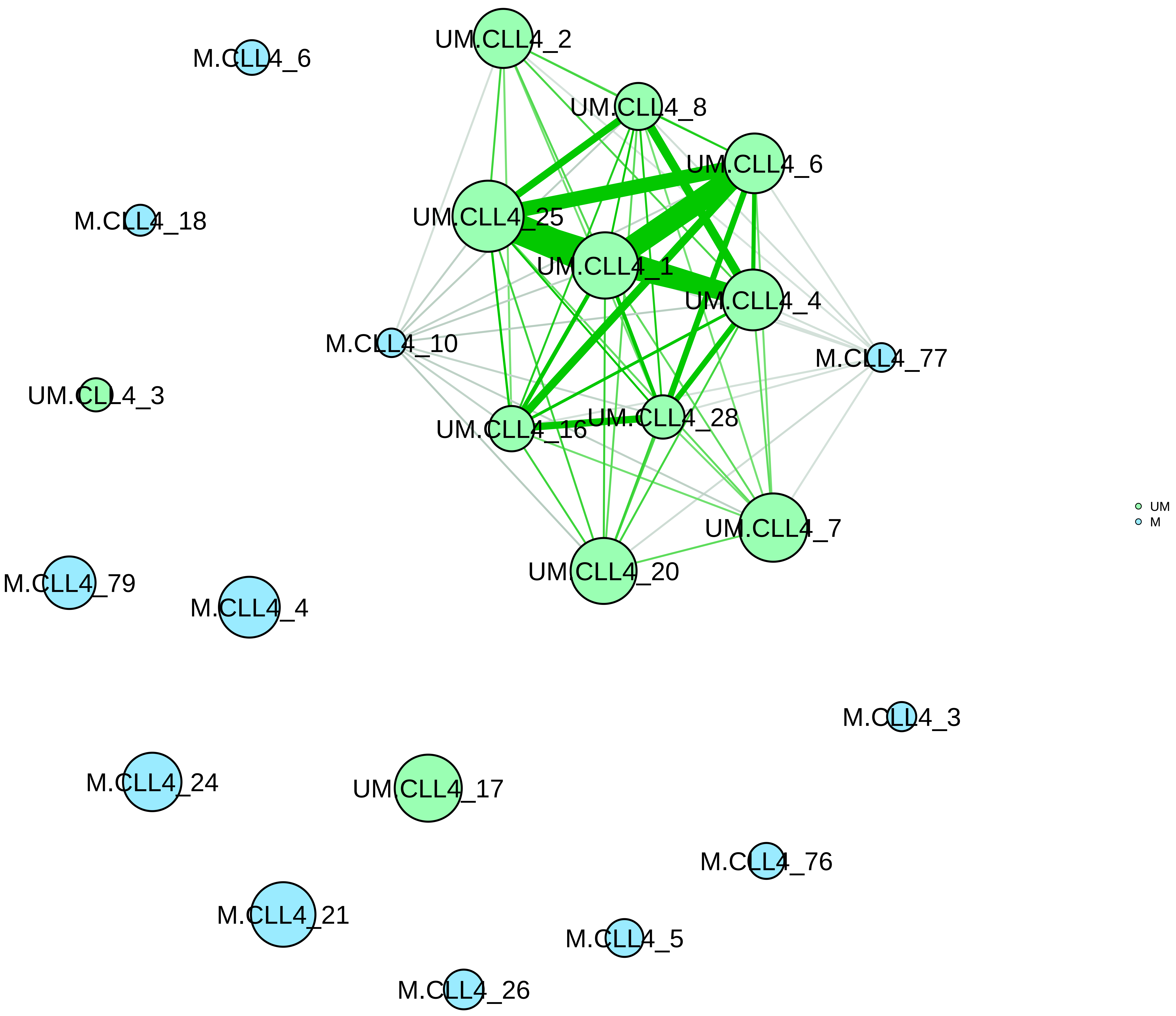

Natural killer cell mediated cytotoxicity || activated in UM with power 4

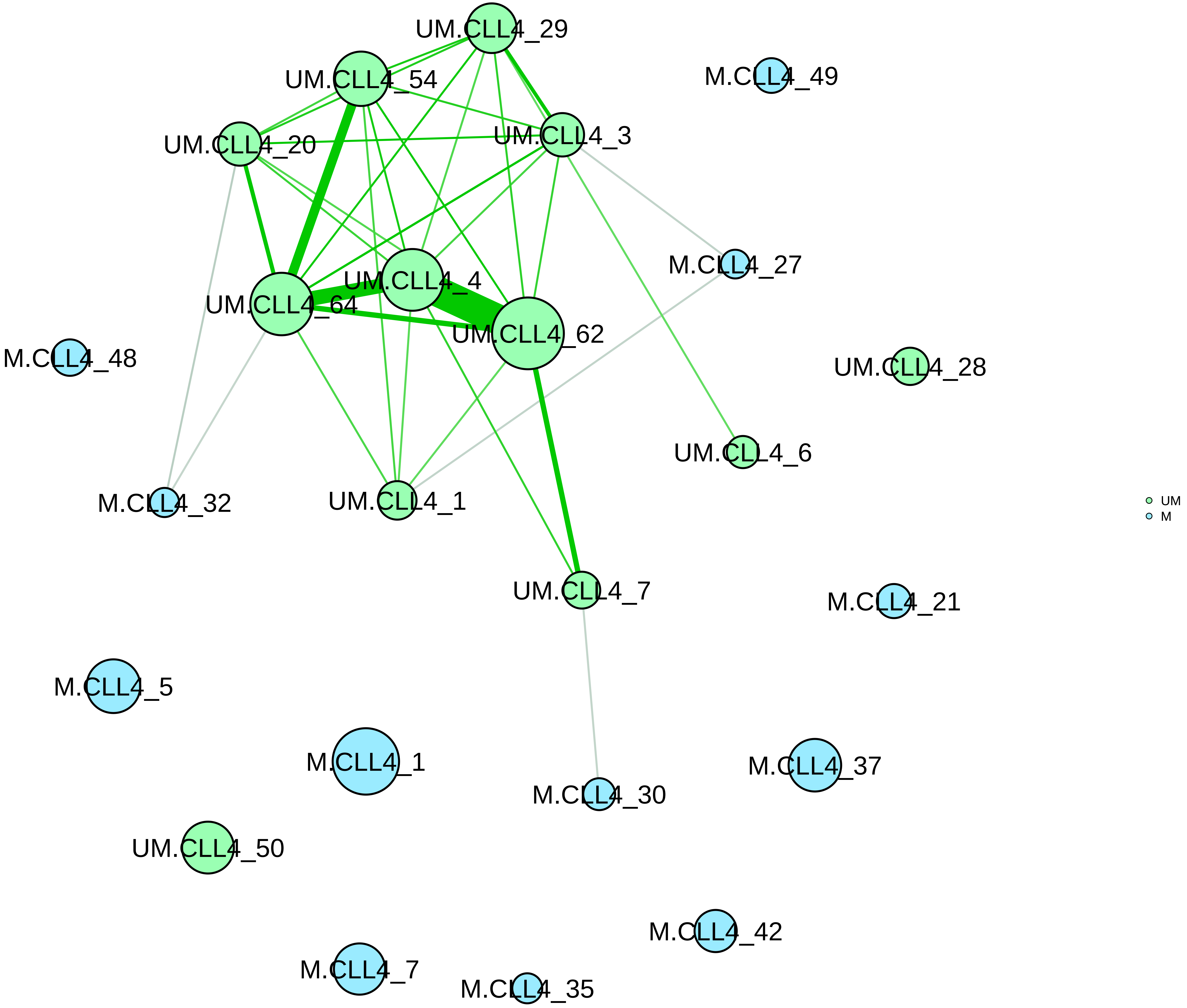

NEGATIVE REGULATION OF CELL−CELL ADHESION || activated in UM with power 1

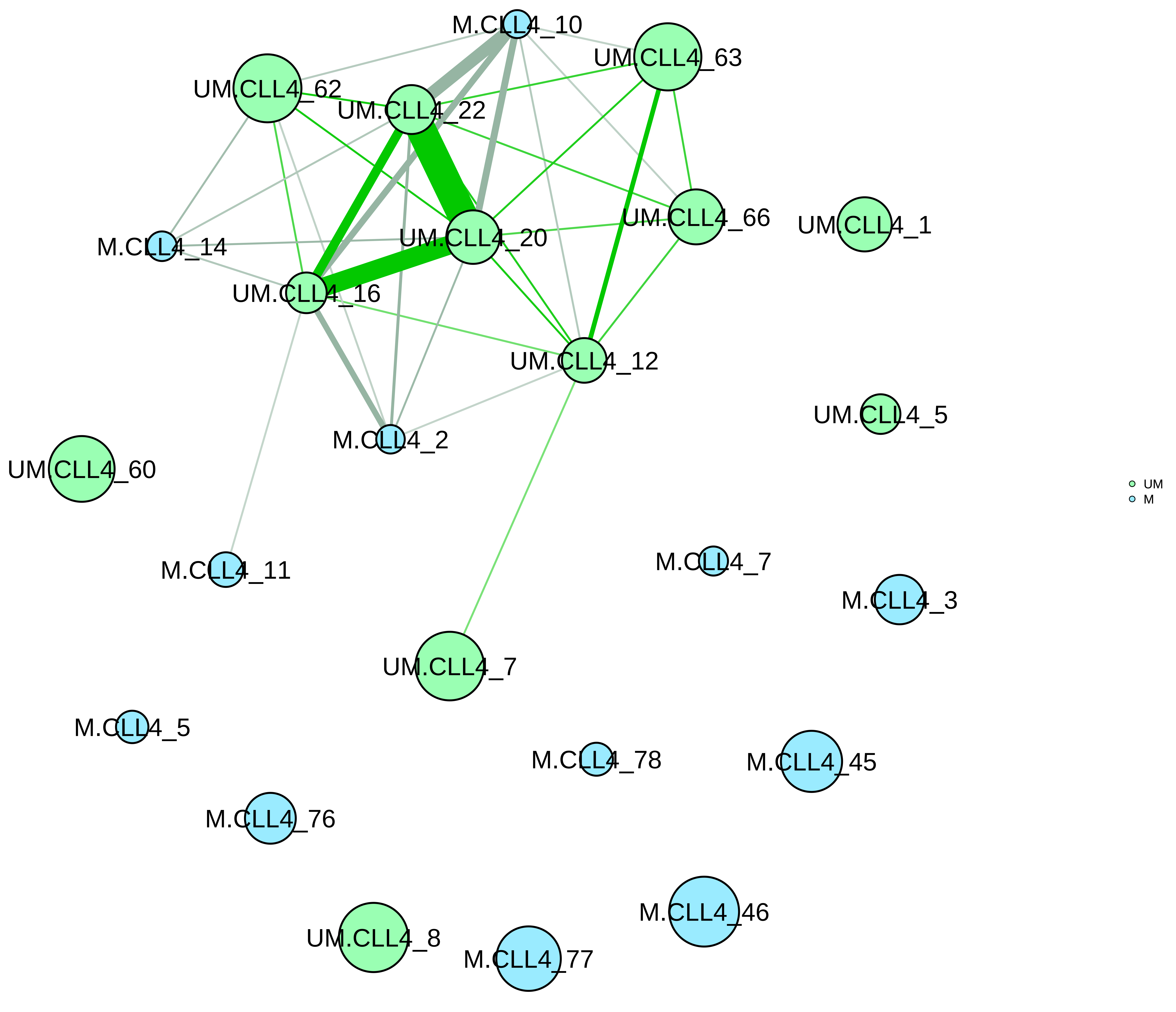

NEGATIVE REGULATION OF LEUKOCYTE ACTIVATION || activated in UM with power 3

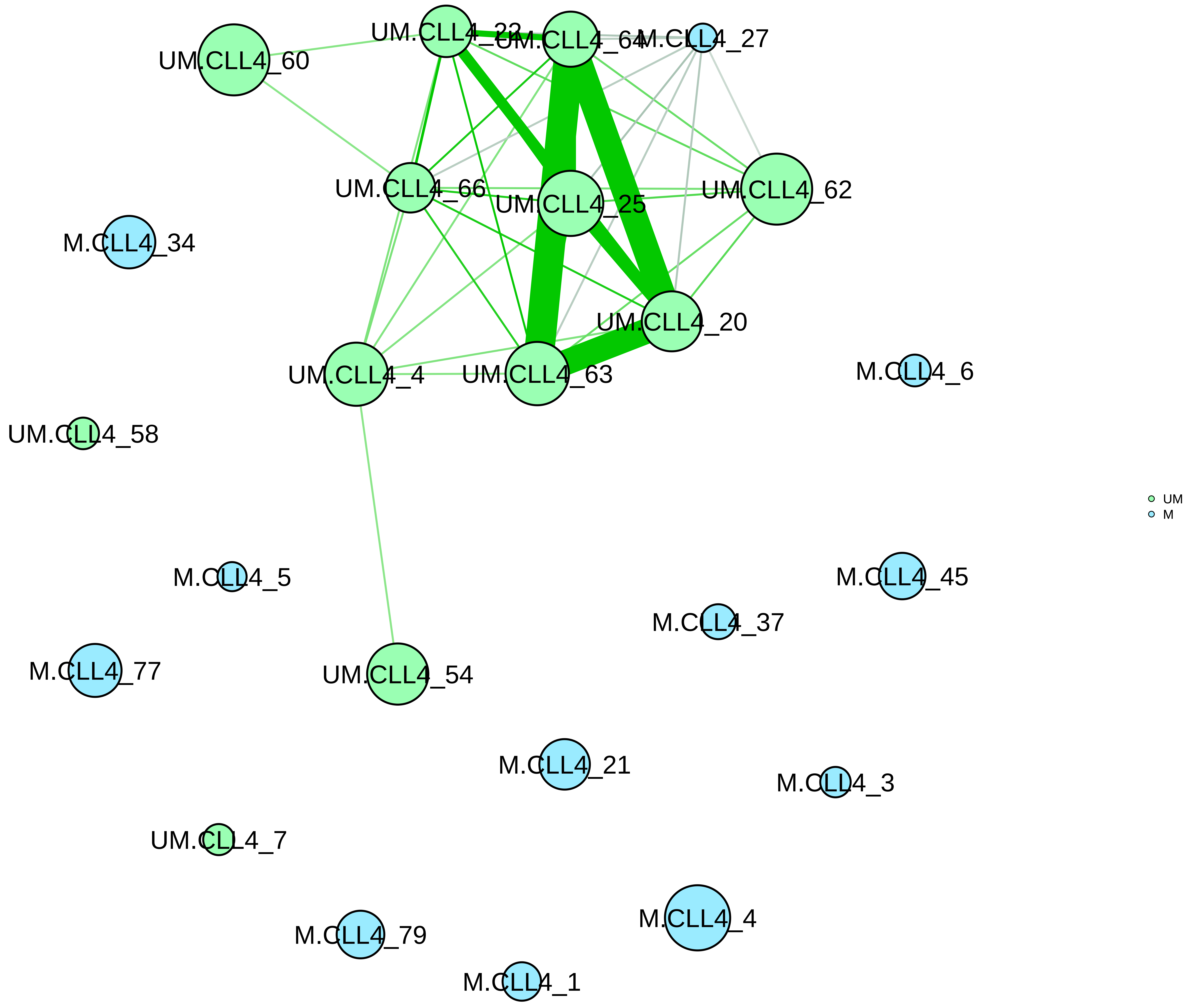

NEGATIVE REGULATION OF LEUKOCYTE CELL−CELL ADHESION || activated in UM with power

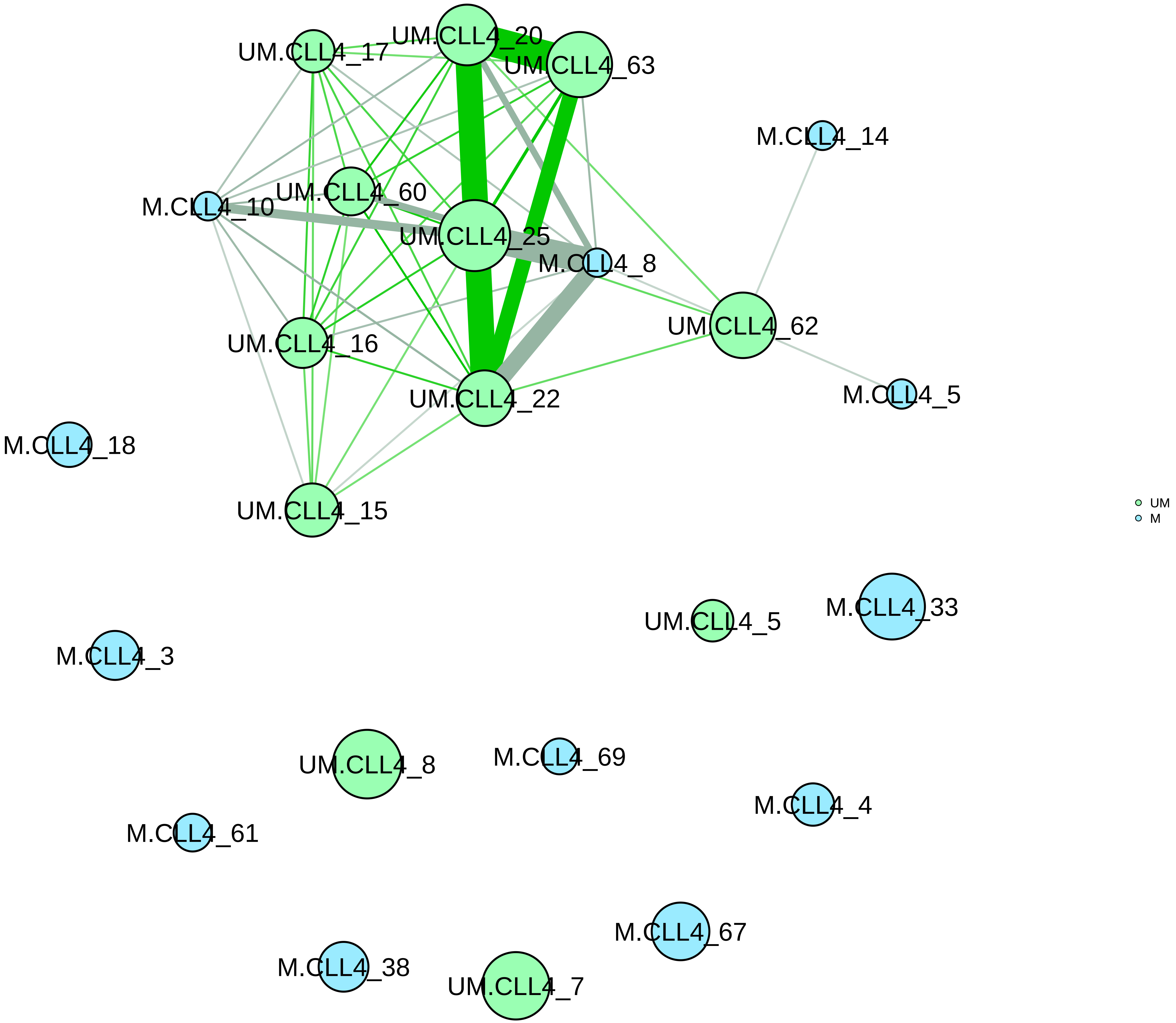

NEGATIVE REGULATION OF LYMPHOCYTE ACTIVATION || activated in UM with power 3

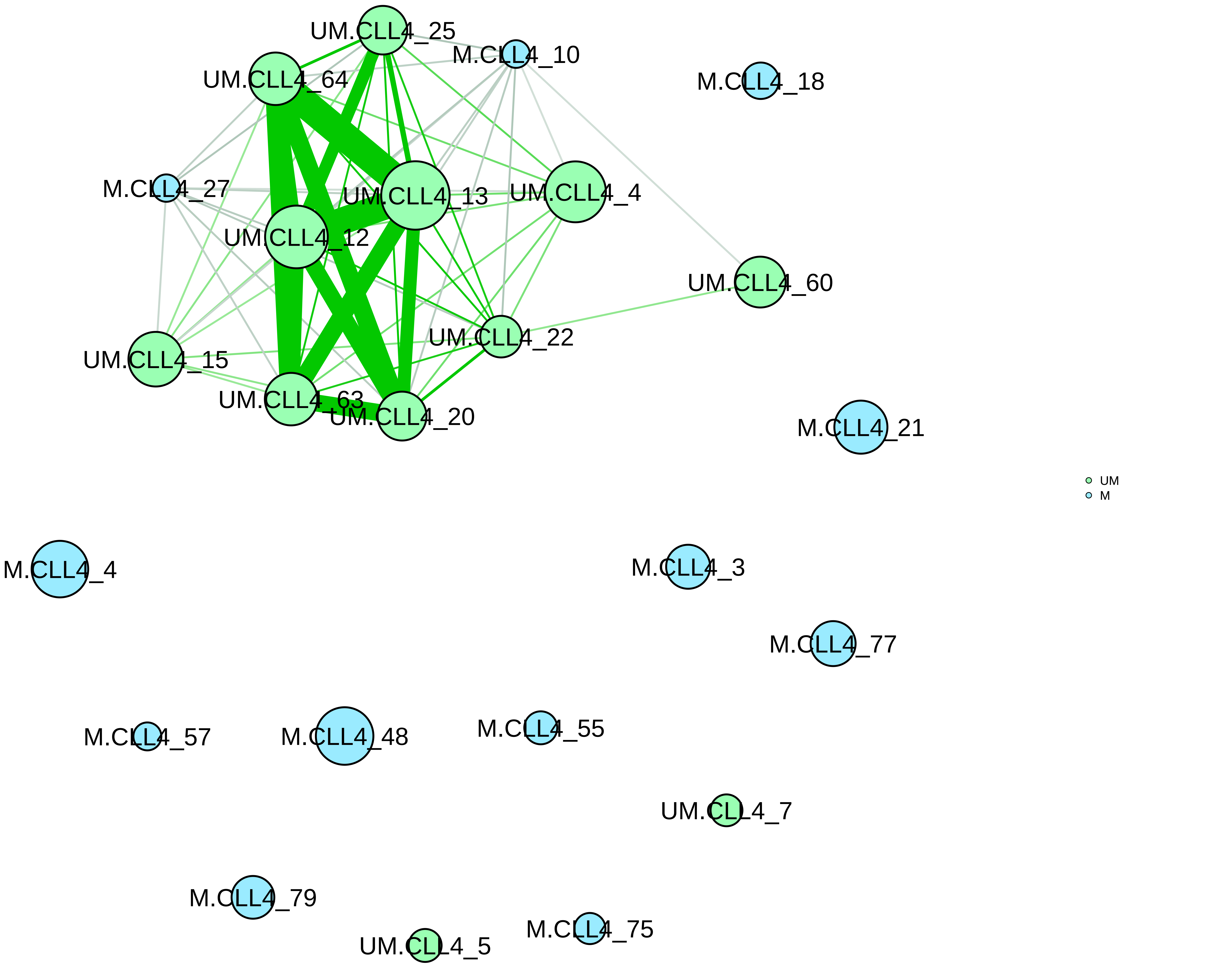

NEGATIVE REGULATION OF T CELL ACTIVATION || activated in UM with power 2

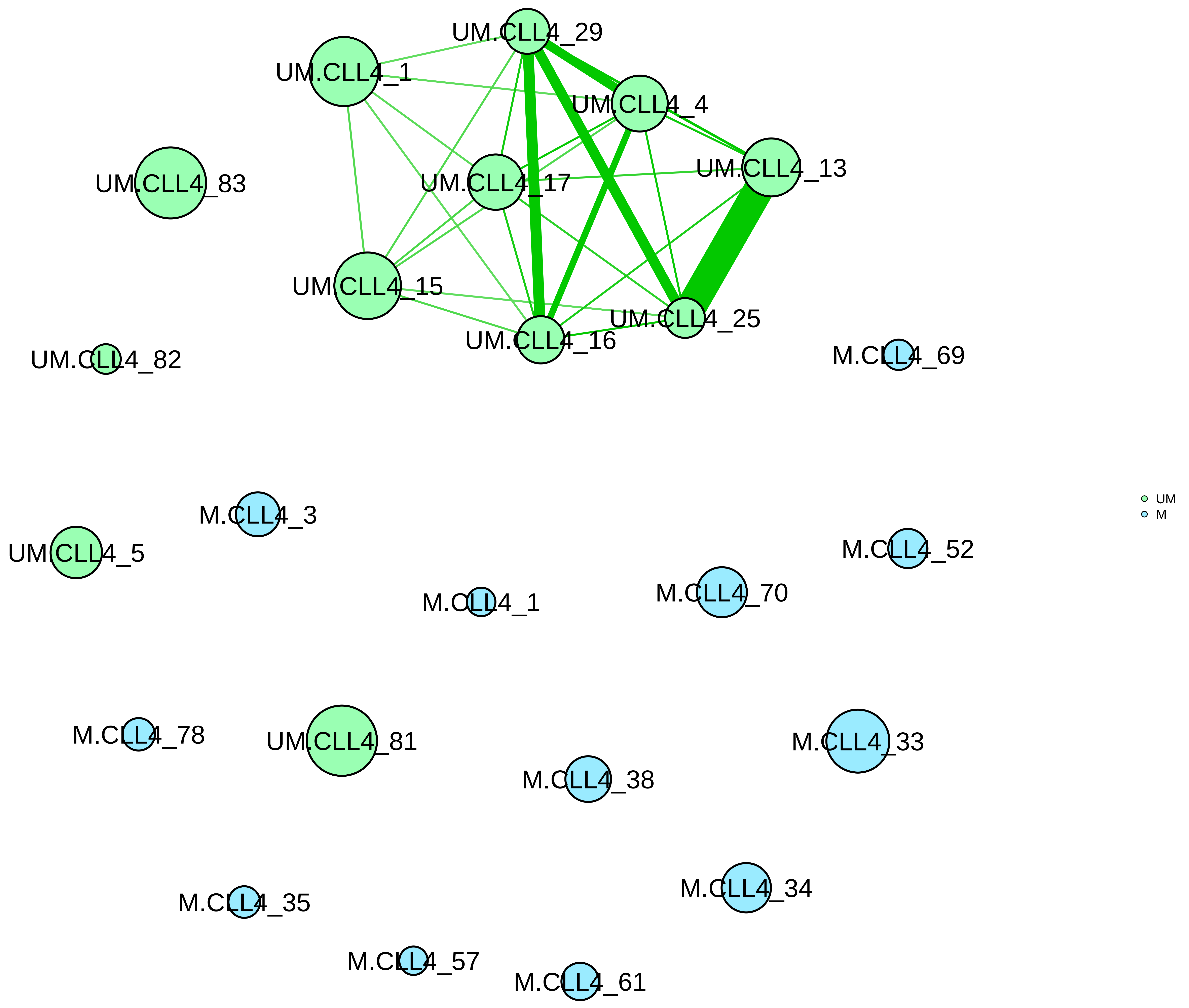

POSITIVE REGULATION OF B CELL ACTIVATION || activated in UM with power 2

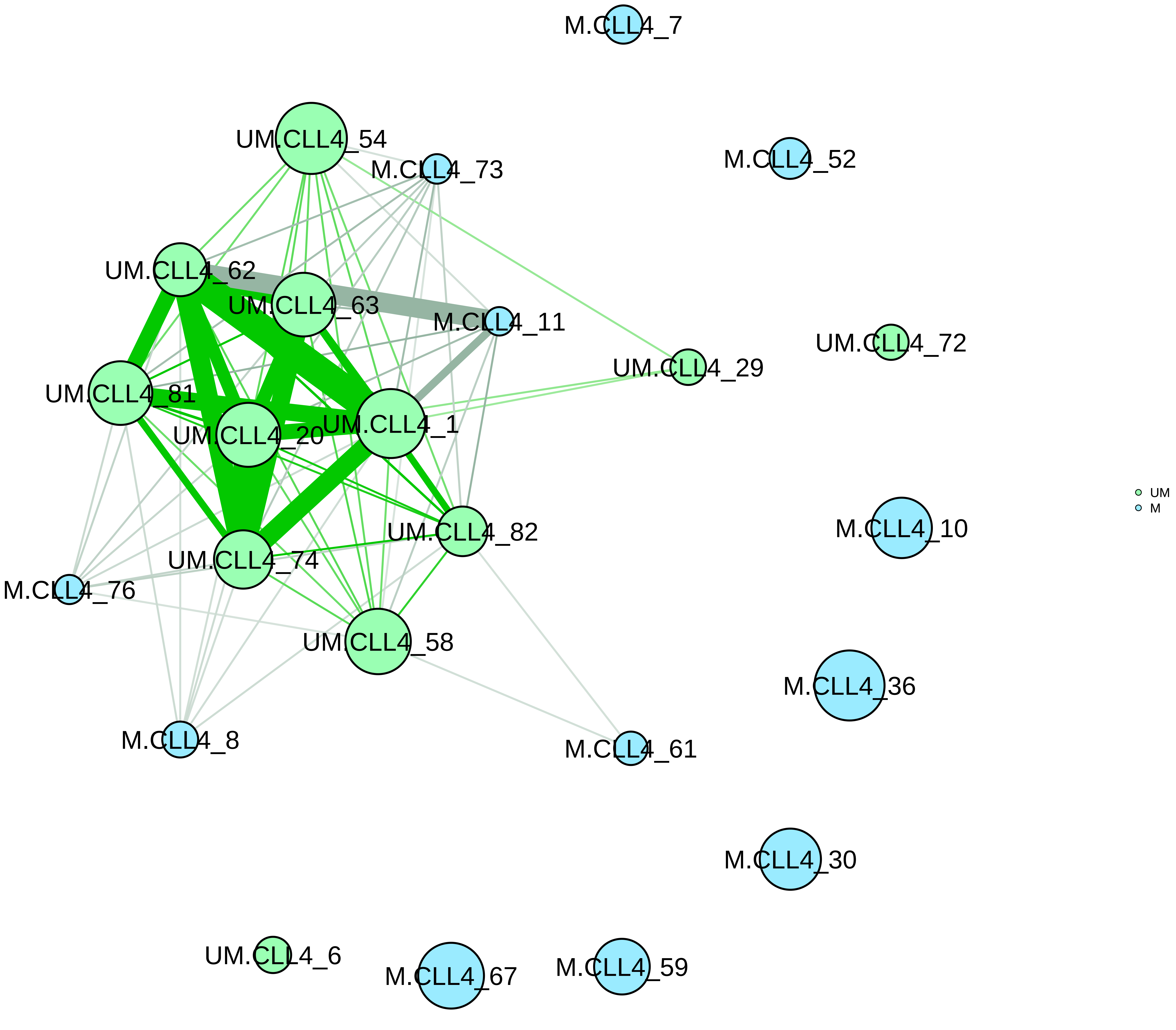

POSITIVE REGULATION OF HEMOPOIESIS || activated in UM with power 3

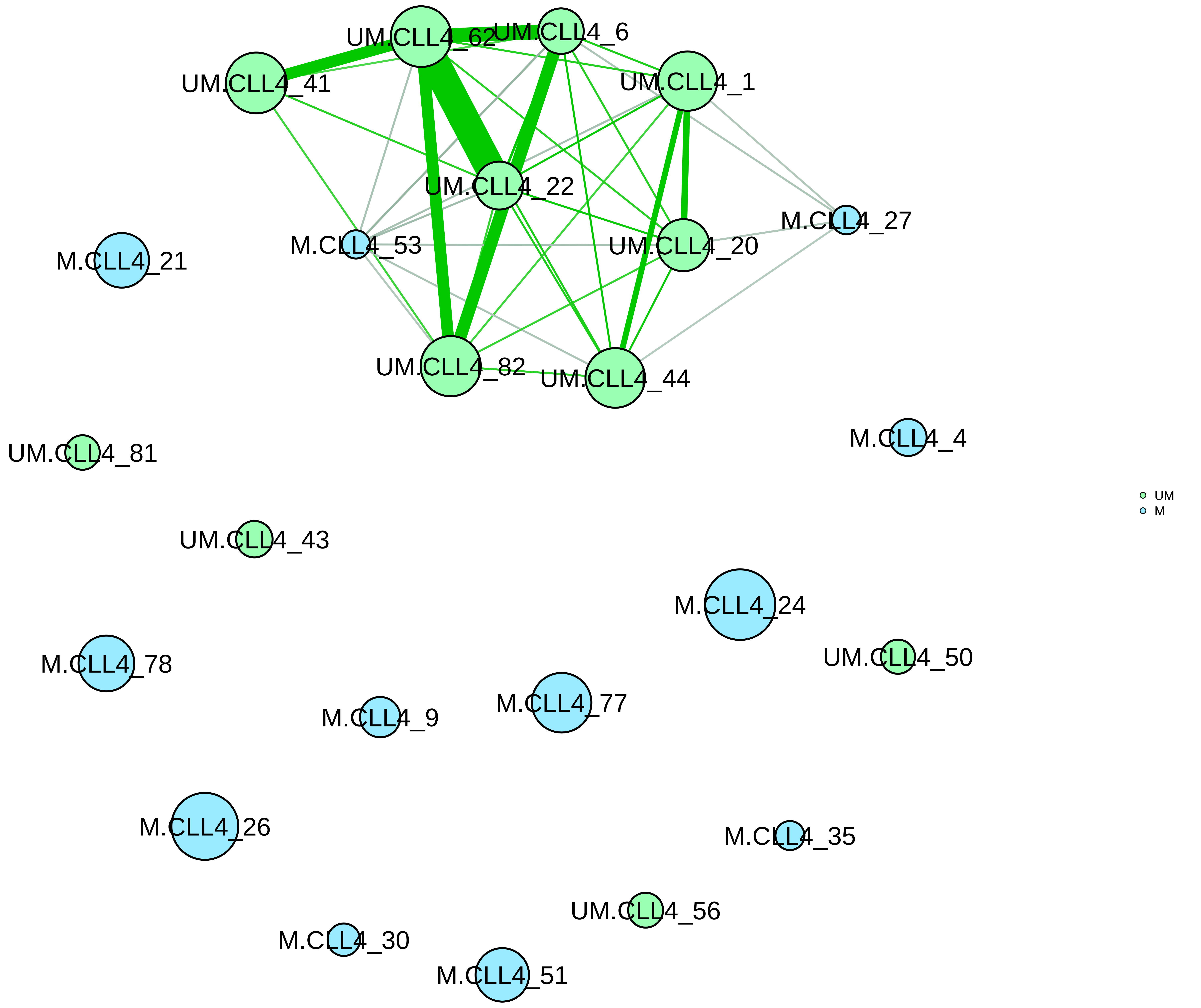

POSITIVE REGULATION OF MAST CELL ACTIVATION || inhibited in UM with power 1

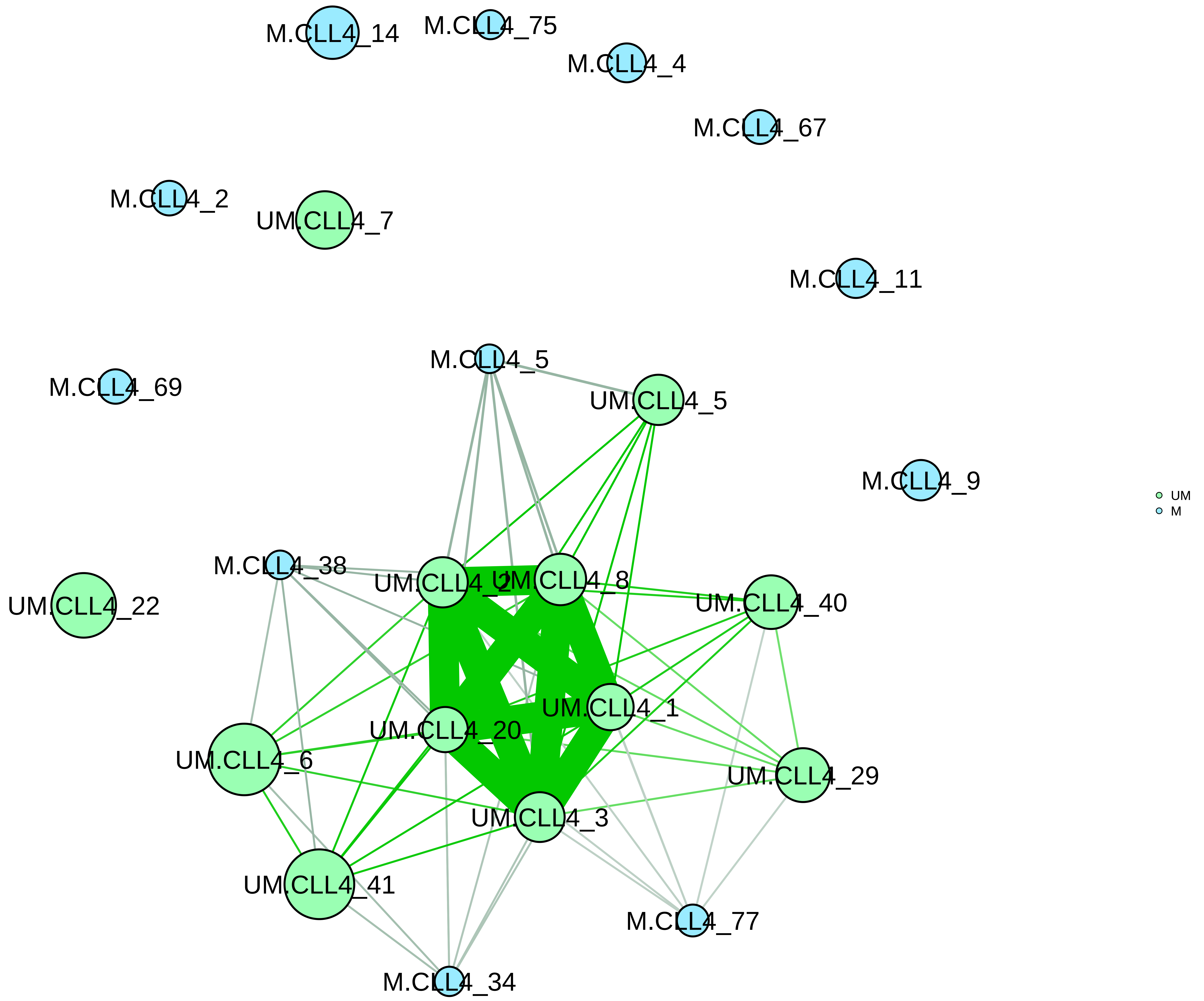

REGULATION OF JNK CASCADE || activated in UM with power 4

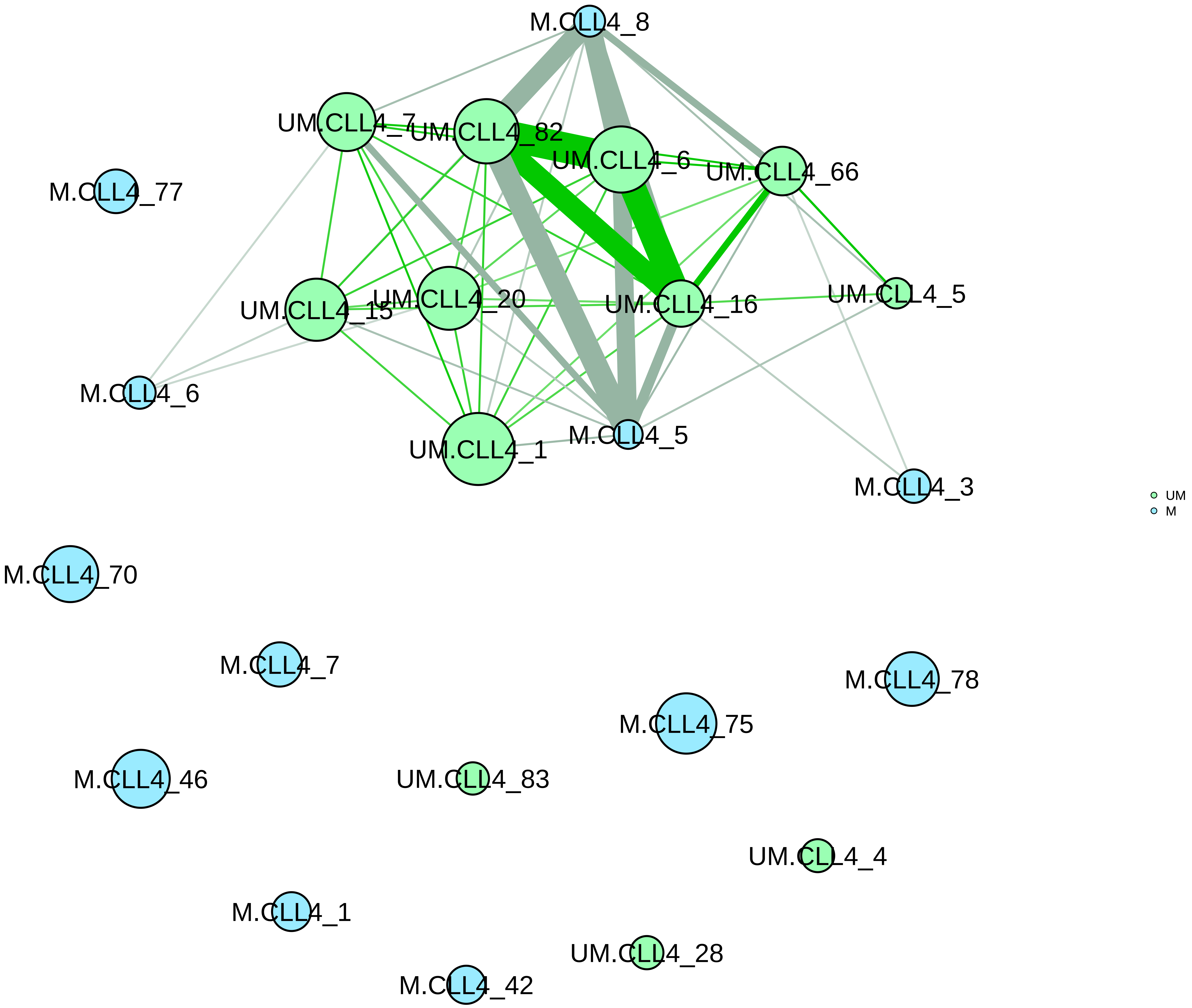

REGULATION OF MAST CELL ACTIVATION || inhibited in UM with power 1

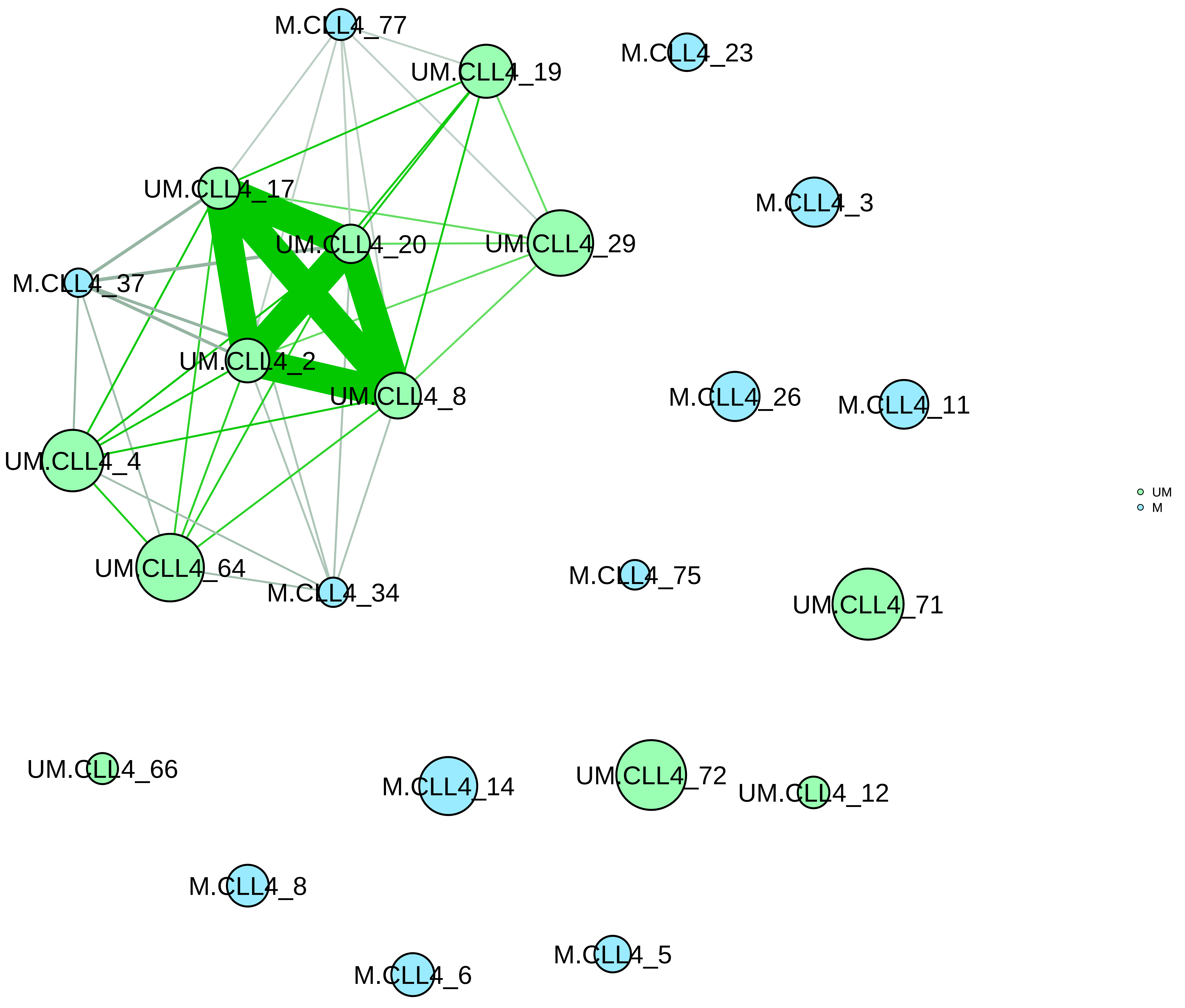

REGULATION OF STRESS−ACTIVATED MAPK CASCADE || activated in UM with power 4

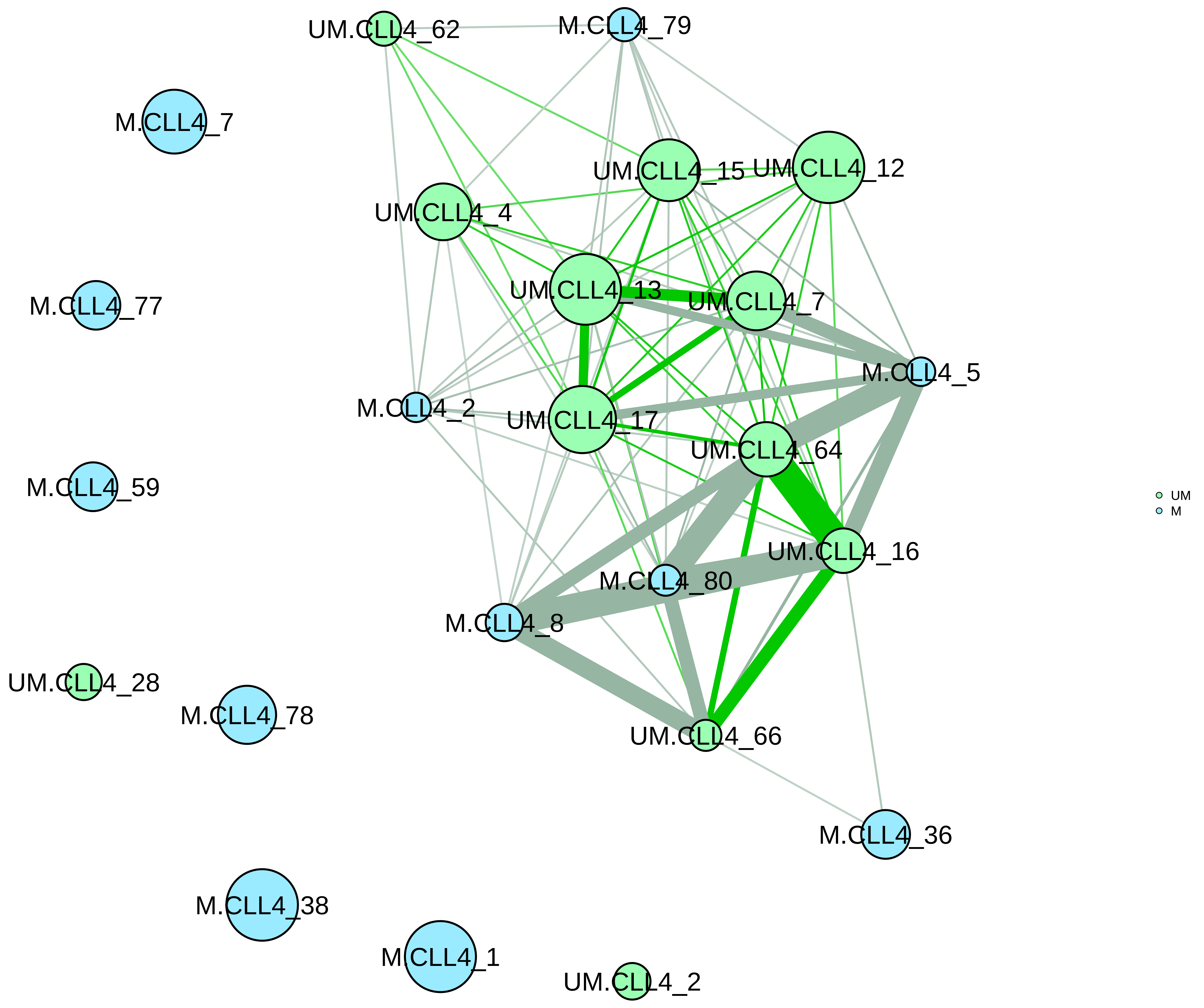

REGULATION OF SYNAPTIC PLASTICITY || inhibited in UM with power 1

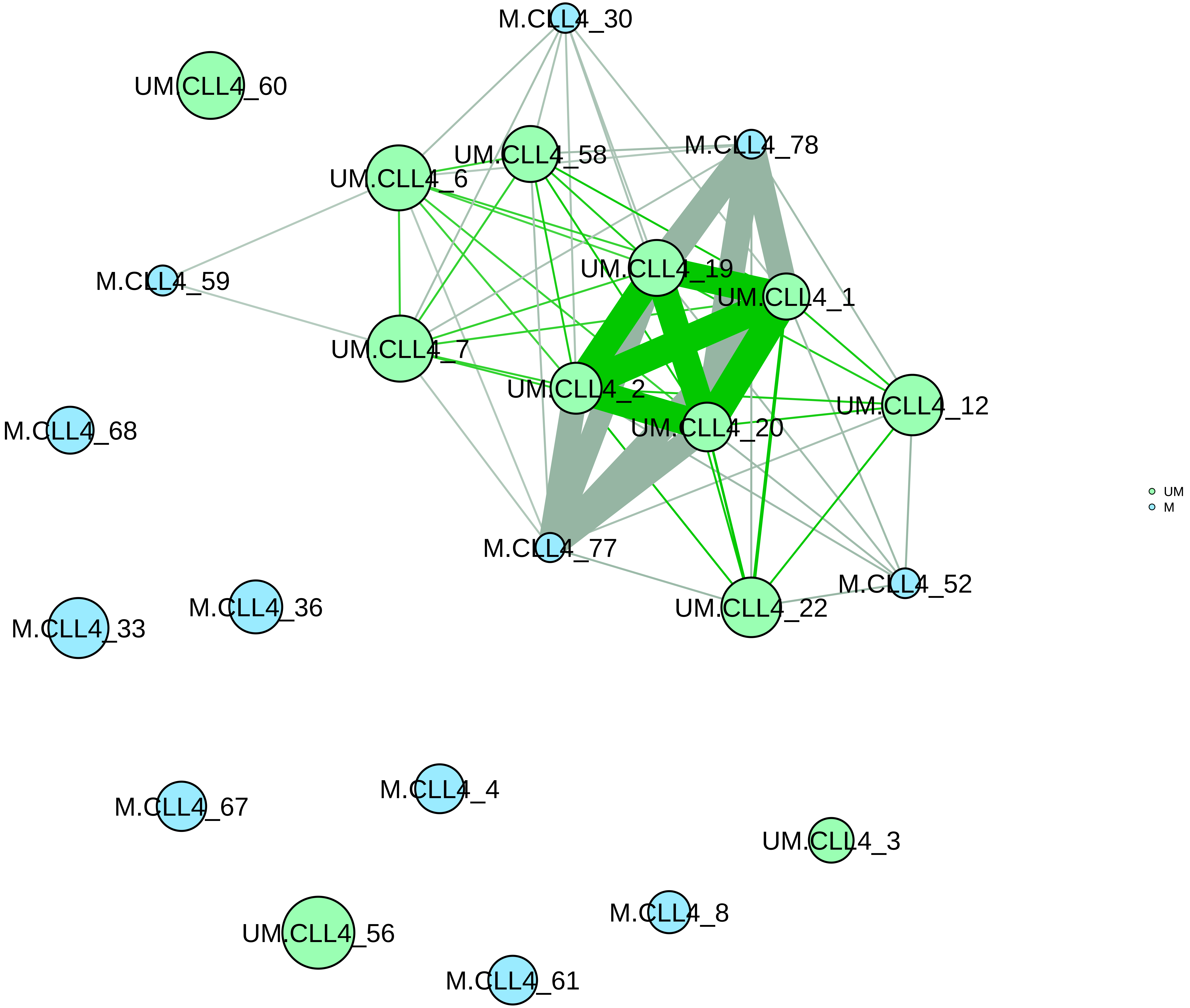

Supplement: S2 File — File of patient similarity networks. Each PSN is the representation of the patient similarities related to a specific pathway which has been found enriched and predictive in classifying the CLL patients. (PDF) [file pcbi.1012022.s004.pdf]
