# Supplementary material for: StellarPath: Hierarchical-vertical multi-omics classifier synergizes stable markers and interpretable similarity networks for patient profiling
Source: PLoS Comput Biol. 2024 Apr 12;20(4):e1012022. doi: 10.1371/journal.pcbi.1012022 (PMC11042724; doi:10.1371/journal.pcbi.1012022)
Supplement: Attachment — Submitted filename: comments-StellarPath-202312.pdf [file pcbi.1012022.s005.pdf]

Remarks to the Authors:

In this manuscript, Giudice et al. described a new method called StellarPath for patient classification, leveraging bioinformatics techniques to select biologically meaningful molecular and pathway features. StellarPath analyses multi-omics datasets, performs a feature selection to identify markers, integrates the different omics in a hierarchical-vertical integration flow and employs a unique strategy by training a GCN on each pathway-specific PSN to classify unknown patients. StellarPath represents a novel patient similarity network (PSN) paradigm, and outperforms other PSNs in terms of classification performance and computational resources across seventeen datasets, which is a significant contribution to the field of precision medicine. Their method has the potential to be more robust than the currently available methods. However, there are several significant weaknesses in the manuscript, regarding quality of presentation, as well as methodology, validity of approach and appropriate use of statistics that impinge on the robustness, validity, and reliability of the conclusions. These issues are highlighted below:

1. The authors suggest that StellarPath is the first method to adopt a hierarchical-vertical integration flow and to adopt a novel similarity measure. Also this work has demonstrated StellarPath superior performance compared to netDx by bridging the gap between machine learning algorithms and precision medicine. Is netDx the popular, traditional or state-of-the-art method that could represent the current existing PSN methods? Similarly, it was said that "To date, no patient classifier fully leverages the paradigm's inherent benefits. PSNs remain complex, unexploited, and meaningless" in the abstract, however, an article in https://www.ncbi.nlm.nih.gov/pmc/articles/PMC6921439/ mentioned several methods that might do these jobs, please clarify it. Overall, they should provide more evidence, such as comparative analysis with other state-of-the-art methods in terms of efficiency and effectiveness to support that StellarPath addresses the limitations of current approaches or outperforms them.
2. Vertical and hierarchical? Is there a strong reason to suggest "vertical" in addition to "hierarchical" which seems to be very inclusive already. If this is the case, authors shall be cautious about claiming "the first" on page 36. And "patient learning" does not seem to be a standard termology.
3. The authors mention that StellarPath provides new insights about patients that enable subsequent analyses like stratification. However, they should elaborate on how these insights can be used in practice and what specific applications they could have in precision medicine.
4. on pages 22-24, authors can further explore whether the additional pathways/processes StellarPath identified can indeed provide more accurate biological insights, instead of just comparing numbers, especially considering that there have been numerous studies contributing to biological stories behind pathology behind these 17 test datasets. Efforts in this direction are much better valued by biologists.

5. The authors mention that StellarPath selects many deregulated pathways and molecules that are correctly associated with Hypoxia and the Disorder of Glucose Metabolism. However, they should provide more details on how these findings can be used in practice and what specific applications they could have in precision medicine.
6. Section “Patient classification” is tedious, could it be simplified or assisted by a diagram?
7. Column ‘leadingEdge’ in sheet ‘Table 15_GSEA_prerank’ and ‘Table 17_unique_GSEA’ of Supplementary2.xlsx lacks contents, and which sheets list the markers you found? There are too many tables, which might be integrated into less tables to only deliver necessary and easy-interpretable information to readers.
8. What is the overview plot of patient similarity network (i.e. like Fig. 10 in reference PMC6921439) for all the sixteen public datasets based on StellarPath? Since the overview plot could help readers to identify the robustness of StellarPath.
9. It seems like that StellarPath could not be installed, giving errs information in installation (Downloading GitHub repo LucaGiudice/StellarPath@HEAD

   Error in utils::download.file(url, path, method = method, quiet = quiet, :

   download from 'https://api.github.com/repos/LucaGiudice/StellarPath/tarball/HEAD' failed.). Therefore, we could not learn the robustness, validity, and reliability of StellarPath in practice.
10. Need to work out Fig. 1’s captions, leaving many terminologies unexplained, e.g., cohesiveness, separability power, stability, etc.
11. Measures on classification performances are too limited, shall include PPV, NPV, AUC, etc. Besides, error bars of StellarPath GCN seem to be too wide, could authors clarify?
12. It seems that “validation” was loosely used. In machine learning, this step is typically for tuning hyper-parameters.
13. The structure of the manuscript is a bit confusing. The author appears to calculate a PSN for each pathway, but this is not clearly explained in the Methods section; instead, it is elucidated in the Computational resources section. Additionally, the Computational resources section could be at the last subsection in the Methods section.
14. The resolution of most images is insufficient. In Figure 6 (C), the color of edges is difficult to distinguish. It is recommended to modify Figure 6 (C) and add graphics of important PSNs.
15. There are some grammatical issues in the manuscript, for instance, ‘The Separability Power (Method 2.4) and the Jaccard Index.’ Additionally, certain

expressions, like ‘aka,’ are not suitable for a research paper. It is advisable to revise the manuscript carefully.

16. There are some formatting irregularities, including the pseudocode format for the GraphSAGE algorithm and the format of tables. Furthermore, there is inconsistency in the font styles of many variables throughout the entire manuscript, and it is recommended to use italics for all variables. It is advisable to revise the manuscript carefully.
17. It is hard to understand some descriptions in the Methods section because there are some contradictions when explaining the details of StellarPath. Hope the author will check the inconsistency. Specifically, in the last paragraph of the Feature selection section, the author described the standard for selecting activated/inhibited pathways. However, in the next section, it mentioned, ‘Conversely, if the pathway is inhibited (i.e., negative expression log fold change of its molecules),’.
18. In the Abstract, the author mentioned that 17 datasets were used when testing StellarPath's computing resources and classification performances, but only 16 datasets were shown in the figures presenting the result these two experiments. It is suggested to check for this conflict.
19. It is best to provide exact data and images as evidence for the following statement, ‘In conclusion, GSEA finds many more pathways than StellarPath, some of them are clearly connected to OGD but many of them are generic or seemingly unrelated to the classes in comparison.’
20. The formula for the Stability Component is most likely written incorrectly. The summation symbol should be outside the semicolon.
