# Supplementary material for: StellarPath: Hierarchical-vertical multi-omics classifier synergizes stable markers and interpretable similarity networks for patient profiling
Source: PLoS Comput Biol. 2024 Apr 12;20(4):e1012022. doi: 10.1371/journal.pcbi.1012022 (PMC11042724; doi:10.1371/journal.pcbi.1012022)
Supplement: Attachment — Submitted filename: Rebuttal letter and Replies.pdf [file pcbi.1012022.s006.pdf]

UNIVERSITY OF EASTERN FINLAND

Cover letter

February 22, 2024
Giudice et al., StellarPath: hierarchical-vertical multi-omics classifier synergizes stable markers and interpretable similarity networks for patient profiling

Dear Editors and Reviewers,

We would like to express our sincere gratitude to all the editors and reviewers who have been involved in the proceeding of our work. We are grateful for your appreciation of our work and for the opportunity to refine our manuscript. Your input emphasized the importance of articulating the methodological challenges tackled by our work, StellarPath role in precision medicine, and the practical applications of our findings. Your advice led us to enhance the manuscript's clarity and detail, especially in discussing the pathways identified by netDx and GSEA. We trust these revisions have addressed your concerns, aiming for the updated manuscript to meet your expectations.

Sincerely,
Luca Giudice

A.I. Virtanen
Institute for
Molecular Sciences
neulaniementie 2
p.o. box 1627, fi-70211
kuopio, finland
tel. +358 207 87 2211
(operator)
fax +358 17 163 030

A.I. Virtanen -
instituutti
neulaniementie 2
pl 1627, 70211 kuopio
puh. 0207 87 2211
(vaihde)
faksi 017 163 030

www.uef.fi

Cover letter

Legend:

- Reviewer/Editor comment
- Author reply

Reviewer 1 Comment 1:

Is netDx the popular, traditional or state-of-the-art method that could represent the current existing PSN methods? Similarly, it was said that "To date, no patient classifier fully leverages the paradigm's inherent benefits. PSNs remain complex, unexploited, and meaningless" in the abstract, however, an article in https://www.ncbi.nlm.nih.gov/pmc/articles/PMC6921439/ mentioned several methods that might do these jobs, please clarify it. Overall, they should provide more evidence, such as comparative analysis with other state-of-the-art methods in terms of efficiency and effectiveness to support that StellarPath addresses the limitations of current approaches or outperforms them.

Reply:

Thank you for your feedback. We recognize that our research focuses on a niche area with few directly comparable published methods. However, we see this aspect as an advantage rather than a limitation that we must necessarily overcome.

We developed StellarPath to address the challenges of implementing a classifier based on the PSN paradigm, to overcome the inherent and hidden limitations of using networks as features, and to propose new strategies for enhancing the application and interpretation of PSNs. StellarPath also highlights new directions for those aiming to develop this paradigm further.

Our selection of a competitor was based on two criteria: First, the workflow of data analysis which had to include patient similarity networks, feature selection, data integration, and classification. Second, the goal had to be to identify interpretable, predictive, and biologically meaningful PSNs.

netDx emerged as the most suitable competitor. It parallels StellarPath's workflow in terms of data processing, classification, and results. It creates PSNs from multi-omics data, filters the networks, predicts the patients on the selected networks, does not innovate the algorithm of node prediction (GeneMania), focuses on interpretable machine learning, and is configurable to run with pathways. netDx as StellarPath has the goal to identify interpretable, predictive, and biologically meaningful PSNs that can provide new insights about the patients and be used for further analytical operations. Finally, netDx's significant impact, indicated by 71 citations in three years, underscores its novelty and relevance in this niche.

We excluded methods that did not meet these criteria. For instance, while a conventional random forest may be faster and offer similar classification performance, it lacks data processing, omics integration, and network selection; all characteristics that are crucial in StellarPath's workflow for delivering novel and meaningful insights. This difference is important to consider, as a direct comparison might lead to misleading conclusions. The "Figure 2 Performance benchmarking with PanCancer Survival data" in the netDx article proves our point. Canonical classifiers (e.g. regression, SVM, Random Forest) show similar classification performances to netDx and are "apparently" weakening its scientific contribution. Conversely, it is also possible to encounter the opposite scenario. If we were to compare StellarPath against a method that presents a unique prediction algorithm but lacks feature selection in its workflow, StellarPath would inherently have an unfair advantage.

Cover letter

In the article that you mentioned (PMC6921439), MultiSourcDSim and the related cited methods (in cit. 18, 19, and 20) significantly differ from StellarPath as they focus on disease similarities from published annotations rather than on the comparison of the patients (or samples) under study (or experiment). Methods like MultiSourcDSim, analyze semantic similarities between disease terms, aiming to understand and quantify the relationships between the different diseases. They exploit ontologies and medical vocabularies to assess how closely related various disease terms are, based on their descriptions and contexts within biomedical literature or databases. The main goal of these methods is to uncover potential connections between diseases that may not be immediately apparent, facilitating the discovery of novel disease correlations, understanding translational disease mechanisms, and identifying new applications for already existing treatment strategies. Specifically, MultiSourcDSim compares diseases annotated with terms from the CTD's MEDIC disease vocabulary database, 10.1016/j.jbi.2011.11.017 uses the Gene Ontology database and PMC3041538 uses Disease Ontology database. None of these methods analyses high-throughput omics data or experimental data describing patients. None of these methods helps the biologist or the clinician to profile the subjects and the classes under study. Plus, these methods depend on the quality and quantity of the disease annotations. They cannot be easily used to study the similarity between treatments, drug effects, cell lines, genotypes, and any clinical or biological information related to the samples of an experiment.

This difference in both workflow and objectives makes them unsuitable comparators for StellarPath.

It is also important to say that we deliberately positioned StellarPath within a niche, highlighting in the Introduction and Discussion sections the importance of an improved implementation of a classifier based on the PSN paradigm.

Reviewer 1 Comment 2:

Vertical and hierarchical? Is there a strong reason to suggest "vertical" in addition to "hierarchical" which seems to be very inclusive already. If this is the case, authors shall be cautious about claiming "the first" on page 36. And "patient learning" does not seem to be a standard termology.

Reply:

Thank you for the questions.

About the first one, we used the term "hierarchical-vertical" based on the definitions provided by Gliozzo et al. (https://doi.org/10.1093/bib/bbac207) in their review of the methods based on PSNs. They defined three different types of integration flows: horizontal, hierarchical-vertical, and parallel-vertical, so we followed their annotation.

About the second question, we have not found another method that models multi-omics data into PSNs with a hierarchical-vertical integration flow, so we wanted to push this characteristic of our software. It is also a way to highlight the importance and the contribution of such integration flow which is rarely implemented (even in general) because it requires modeling each interaction between omics and avoids simply feeding all the omics into the classifier (which is the usual strategy).

Thank you also for pointing out that "patient learning" is not standard. We replace it with the canonical "patient profiling". In this way, we hope to highlight that our method produces data capable of describing the patients under study.

Cover letter

Reviewer 1 Comment 3:

The authors mention that StellarPath provides new insights about patients that enable subsequent analyses like stratification. However, they should elaborate on how these insights can be used in practice and what specific applications they could have in precision medicine.

Reply:

Thank you for your comment. Initially, our focus was on the methodology. However, we agree that including more details on how users can practically exploit our method's results can significantly enrich our work. This addition will also offer a deeper understanding of our method's applicability in the field of precision medicine.

According to this comment, we add a new section called “Precision Medicine” in the manuscript. This section outlines the operations that can be performed with the output data of StellarPath and discusses their implications in precision medicine.

Reviewer 1 Comment 4:

on pages 22-24, authors can further explore whether the additional pathways/processes StellarPath identified can indeed provide more accurate biological insights, instead of just comparing numbers, especially considering that there have been numerous studies contributing to biological stories behind pathology behind these 17 test datasets. Efforts in this direction are much better valued by biologists.

Reply:

Thank you for your comment. We value your interest in increasing the biological significance of our research. Therefore, we are fully committed to addressing this matter.

We researched the predictive and significant pathways detected by StellarPath but not by netDx across three datasets. Precisely, we focused on predictive pathways of the late cancer stage and their annotated associations with key characteristics of the late stage such as tumor progression, immune system evasion, and metastasis.

In the end, according to this comment, we make the following changes to our manuscript:

- We update the supplementary table 6 (now table 5 due to Rev1 Comment 7) to indicate those pathways found only by netDx and not by StellarPath.
- We update the supplementary table 8 (now table 6 due to Rev1 Comment 7) to indicate those pathways found only by StellarPath and not by netDx.
- We add a new paragraph in the “Predictive Pathways” section where we discuss the pathways found uniquely by StellarPath in classifying the patients of three datasets.

Reviewer 1 Comment 5:

The authors mention that StellarPath selects many deregulated pathways and molecules that are correctly associated with Hypoxia and the Disorder of Glucose Metabolism. However, they should provide more details on how these findings can be used in practice and what specific applications they could have in precision medicine.

Reply:

In response to your feedback, we have explored practical applications of StellarPath's findings using the OGD dataset, which provides straightforward implications in precision medicine due to the simple phenotypes in comparison.

We add the result of this research in a new paragraph at the end of the section “Validation Results” (now called “Biological Relevance” due to Rev1 Comment 12).

Reviewer 1 Comment 6:

Section “Patient classification” is tedious, could it be simplified or assisted by a diagram?

Reply:

We appreciate the feedback regarding the 'Patient classification' section of our paper, which describes how StellarPath uses a graph convolutional neural network (GCN) for predicting patient classes/labels in the PSNs. While our initial goal was to create a self-contained section, we recognize that it may be overly detailed and distract from StellarPath's innovations.

For this reason, we align with your comment and simplify this part so that the readers can focus their attention on the innovative aspects and results of our method. Precisely, the revised section keeps the key aspects such as how StellarPath prepares the patient similarity networks and feeds them into the neural network. However, we remove the definition of the GraphSAGE algorithm which is not novel and is much more algorithmic/mathematic than the rest of our manuscript.

Reviewer 1 Comment 7:

Column ‘leadingEdge’ in sheet ‘Table 15_GSEA_prerank’ and ‘Table 17_unique_GSEA’ of Supplementary2.xlsx lacks contents, and which sheets list the markers you found? There are too many tables, which might be integrated into less tables to only deliver necessary and easy-interpretable information to readers

Reply:

Thank you for pointing out the issue with the 'leadingEdge' column in the 'Table 15_GSEA_prerank' and 'Table 17_unique_GSEA' sheets of our Supplementary2.xlsx file. The Gene Set Enrichment Analysis (GSEA) method generates gene lists for this column, which were not properly exported in the Excel sheet. We have now resolved this issue.

Regarding your feedback about the abundance of tables, we agree (we initially thought that it was a good idea to report the same information in different granularities but we probably overdid it). To enhance readability and avoid overwhelming our readers, we re-design the Supplementary 2 Excel file. Specifically, we remove tables that summarize the data of other tables and we compact the tables containing combinable information:

- We remove Table 4_avg_methods_perf which contains average performance metrics
- We remove Table 7_netDx_pathways_type and Table 9_SP_pathways_type which list the types of significant pathways identified by netDx and StellarPath and already reported in tables 6 (now table 2.5) and 8 (now table 2.6).
- We remove Table 10_PSN_Power reporting the power of the PSNs identified by netDx and StellarPath in tables 6 and 8.

- We remove Table 13 which summarizes the number of enriched pathways found by GSEA.
- We compress Tables 14, 15, and 17, about the unique, shared, and overall pathways identified by GSEA. Now there is only table the compact new table 2.8.

Reviewer 1 Comment 8:

What is the overview plot of patient similarity network (i.e. like Fig. 10 in reference PMC6921439) for all the sixteen public datasets based on StellarPath? Since the overview plot could help readers to identify the robustness of StellarPath.

Reply:

Thank you for your question about creating an overview plot for StellarPath like Fig. 10 in PMC6921439.

However, it's important to highlight that StellarPath is fundamentally different from MultiSourcDSim. While MultiSourcDSim focuses on semantic similarities between diseases, utilizing the CTD's MEDIC disease vocabulary database (i.e. the similarity is determined based on how the terms of the diseases' annotations are similar), StellarPath analyzes patient similarities based on the molecular profiles produced within a study (i.e. the similarity is determined based on how two molecular profiles similarly regulate coding and non-coding elements). This distinction means StellarPath evaluates patient data within a specific study/experiment rather than evaluating reported and annotated diseases' characteristics across independent studies. As a consequence, creating a unified overview plot connecting all sixteen public datasets is not possible with StellarPath and is not connected to our work (we could potentially apply MultiSourcDSim on StellarPath's results to understand how all the early and late stages of different cancer types are similar but it wouldn't be meaningful for our project).

Recognizing potential confusion between MultiSourcDSim and StellarPath, we decided to highlight the distinct approaches and applications of the two methods.

MultiSourcDSim calculates the similarity between diseases. It uses data from gene-disease associations, GO biological process-disease associations, and symptom-disease associations to form an understanding of the relationships between various diseases. These associations are extracted from a database called CTD's MEDIC disease vocabulary (http://ctdbase.org). The method doesn't directly compare individual patients or biological samples; rather, it focuses on finding similarities and connections between different diseases based on these associations. In fact, they propose a semantic similarity based on MeSH descriptors that help us to understand how biological and medical terms are connected.

1. Data and Methodology:
    - MultiSourcDSim: Analyzes published annotations and associations about diseases.
    - StellarPath: Analyzes multi-omics data of patients divided into two classes in comparison.
2. Results:
    - MultiSourcDSim: Determines similarities between diseases. It builds a disease similarity network. It can reveal unexpected disease similarities and translational treatment strategies.

Cover letter

- StellarPath: Determines markers, pathways and PSNs distinguishing the patients in comparison. It measures the similarity between patients based on high-throughput biological data. It builds patient similarity networks. It builds a trained classifier able to predict the class of new patients described by independent external data.

3. Classification:

   - MultiSourcDSim predicts the category of "unknown" diseases according to the disease similarity network built with databases. It uses a random walk with restart which is a network-based propagation algorithm that does not learn or generalize on trained data. The propagation algorithm assigns a score to the unknown new disease according to the close neighbors and the score is stratified for assigning the category to the disease. At the end of the run, the algorithm is not trained, does not preserve the knowledge of the original disease similarity network, and does not learn from previous data.
   - StellarPath trains graph convolutional networks (GCNs) on pathway-specific patient similarity networks to predict the target clinical condition (anything among, disease, treatment, genotype, cell line, etc.) and test the trained models on unknown patients to understand their ability to generalize. The trained GCNs can be re-used on new multi-omics data describing new patients that are independent of the initial multi-omics data used for training. The GCNs preserve the knowledge of past training.

4. Impact and Application:

   - MultiSourcDSim: Useful for comparing results across different studies, and understanding commonalities and unique aspects of diseases.
   - StellarPath: Suited for patient profiling and precision medicine, helping to understand biological differences between patient classes, to guide treatment strategies, and to formulate follow-up studies.

9. Limitations:

   - MultiSourcDSim: Its effectiveness is limited by the extent and quality of disease annotations in the literature. It cannot effectively analyze diseases that are poorly represented or annotated in existing research.
   - StellarPath: Reliant on the availability of biological data. It cannot utilize pre-existing literature annotations for analysis to avoid bias. It is also true that without biological data that describe the patient's diseases, then also MultiSourcDSim cannot be applied.

10. Extra:

   - MultiSourcDSim: Can be used in conjunction with StellarPath. After that StellarPath has analyzed patient data and provided the disease annotations, MultiSourcDSim can use this information for broader, more generalized similarity analyses between diseases.

Cover letter

Reviewer 1 Comment 9:

It seems like that StellarPath could not be installed, giving errs information in installation (Downloading GitHub repo LucaGiudice/StellarPath@HEAD Error in utils::download.file(url, path, method = method, quiet = quiet, :

download from 'https://api.github.com/repos/LucaGiudice/StellarPath/tarball/HEAD' failed.). Therefore, we could not learn the robustness, validity, and reliability of StellarPath in practice.

Reply:

The error is related to the download process from GitHub, not an issue with StellarPath's code or its implementation. This problem could arise from a range of external factors such as internet instability, GitHub's accessibility, firewall restrictions, or possibly having an outdated version of the devtools package in R.

Having said that, we took this comment as a chance to improve the webpage of StellarPath. We have updated and improved the installation guide for our R package. On the GitHub page of StellarPath (https://github.com/LucaGiudice/StellarPath), we added a "Prerequisites" section to help with the installation of dependencies.

We now offer two installation methods: one utilizing devtools and another exploiting the manual download of the package from GitHub. We suggest attempting the installation process again, following the updated instructions. Additionally, we remind you that we provide a Docker container, ensuring the installation by replicating a pre-tested operating system and environment setup.

Finally, for any other issue, we further advise you to post the problem in the “Issue” section of the StellarPath repository. We will be able to reply you faster and assist you better.

Reviewer 1 Comment 10:

Need to work out Fig. 1’s captions, leaving many terminologies unexplained, e.g., cohesiveness, separability power, stability, et.

Reply:

You are right. Figure 1 presents some terms that are not introduced to the reader yet.

We replace the unexplained terms with other ones that are easier to understand but that still provide the necessary insights to the reader.

Reviewer 1 Comment 11:

Measures on classification performances are too limited, shall include PPV, NPV, AUC, etc. Besides, error bars of StellarPath GCN seem to be too wide, could authors clarify?.

Reply:

In our article, we focus on the Matthews correlation coefficient (MCC) as our primary performance metric, while also reporting accuracy, sensitivity, and specificity in the Supplementary 2.3 table. The MCC is chosen for its suitability in binary classifications with imbalanced classes, characteristic of our TCGA datasets. Plus, MCC natively integrates elements of PPV, NPV, AUC, and AUPR, providing a performance score that summarizes all the others.

MCC is calculated with true positives, true negatives, false positives, and false negatives. This coefficient is highly regarded in binary classification evaluations because it only gives high scores if the model performs well across all four categories of the confusion matrix, considering both classes of patients in the dataset. This characteristic makes it more reliable than metrics like PPV, NPV, AUC, AUPR, accuracy, precision, recall, and F1 score, which can be misleading with imbalanced datasets. In fact, they are prone to evaluate the predictions by using only one class. This can mean overemphasizing the performance of the largest class while underrepresenting the smallest one (or the opposite depending on the user's choice of which class to set as reference).

By focusing on MCC, we aim to provide a more robust assessment of our classifier's performance and to set up a standard for future research about PSN-based classifiers. We also provide further documentation about the performance metrics in the Suppl. 1.8 (now Suppl. 1.9 due to changes based on the Reviewer 1 comment 7).

Regarding the error bars, it is important to clarify that the lines in a violin plot are not error bars.

Rather, a violin shows the distribution of a set of values, in our case, MCC values. The horizontal width of the violin at any level indicates the frequency of values at that level. In other words, a violin extends vertically from the lowest to the highest value in the set, and its horizontal width increases where more values are concentrated.

The considerable vertical range in the yellow violins is due to variations in MCC values assessed with the predictions of the single pathway-specific PSNs. For instance, in analyzing and classifying the OGD samples, some PSNs misclassified the test patients of one class, leading to be poorly evaluated (MCC scores of zero). This explains why the violin extends down to zero and appears wider at this point. In contrast, in the other datasets, the MCC scores from the pathway-specific PSNs were generally above 0.5, leading to a different distribution shape. The violins of the other datasets show the greatest width around the MCC value of 0.9, indicating that the vast majority of the PSNs predicted the testing patients correctly.

We opted for the violin plot because provides a better view of the performances compared to a box plot. A box plot would describe the 25th, 50th (median), and 75th percentiles, obscuring outliers, while a violin plot shows the full range of values.

Reviewer 1 Comment 12:

It seems that "validation" was loosely used. In machine learning, this step is typically for tuning hyper-parameter.

Reply:

We acknowledge the two different usages of 'validation' in our manuscript and clarify their context:

- 'Validation' refers to the subset of the dataset used to evaluate the model (GCN) performance during training. This includes tuning, early stopping, and preventing overfitting, aligning with the typical machine learning use of the term. (Here are the quotes: "At the start, it divides the patients into three sets: training, validation, and testing". "The validation set". "the validation patients". "To assess the effectiveness of this training, StellarPath introduces the validation patients". "Only when a GCN proves proficiency in predicting the class of the validation patients".)

- 'Validation' denotes the process of verifying the biological correctness of the data produced by StellarPath, such as molecules and pathways. (Here are the quotes: “Validation results”. “Molecules and Pathways Validation”. “Post-validation”).

To prevent confusion, we rephrase the sentences in the section “Validation results” to avoid the usage of the “validation” term for the second meaning. Precisely:

- Validation results → Biological Relevance
- Molecules and Pathways Validation → Molecules and Pathways Relevance
- Post-validation → After the annotation

Plus, we improve the explanation about the role of validation patients for tuning the GCNs in the “Patient Classification” section:

- For clarity, we categorize patients as "known" when their class is recognized by the GCN, "unknown" when the GCN is unaware of their class and does not use this information, and "unseen" when they have not been previously evaluated by the GCN. Thus, patients in the training set are known, those in the validation set remain unseen until they are used by the GCN, and patients in the testing set are entirely unknown.
- Here the GCN is called to learn from the training patients and minimize the error of misrepresenting the validation patients in relation to their true class. The minimization is conducted by adjusting the learnable parameters.
- StellarPath introduces the validation patients. These patients, unseen but known, serve as a benchmark for the GCNs.

Reviewer 1 Comment 13:

The structure of the manuscript is a bit confusing. The author appears to calculate a PSN for each pathway, but this is not clearly explained in the Methods section; instead, it is elucidated in the Computational resources section. Additionally, the Computational resources section could be at the last subsection in the Methods section.

Reply:

Thank you for your feedback regarding the manuscript's structure and the clarity of the PSN creation process within StellarPath. Here below, we did a brief recap and analysis of the sentences that mention that one pathway is represented by one PSN and one PSN represents only the patient’s relationships in one pathway:

- Overview section:
    - StellarPath builds pathway-specific PSNs. For each enriched pathway, StellarPath calculates the similarity between pairs of training patients using a novel measure that compares the values of the significant molecules belonging to the pathway.
- Patient similarity networks section:
    - StellarPath builds a patient similarity network per enriched pathway.
    - $Comp1PATHWAY(Pj, Pk)$ = $\sum\sum ii$max min($(mmij\ ij,,mmik\ ik)$) $with\ \forall i \in$ PATHWAY
    - $Comp2PATHWAY(Pj, Pk)$ = $(\sum i|(PATHWAY\ mij{+}mik)|/2)$ $with\ \forall i \in$ PATHWAY
    - $Comp2PATHWAY(Pj, Pk)$ = 1 − $(\sum i|(PATHWAY\ mij{+}mik)|/2)$ $with\ \forall i \in$ PATHWAY
- Network processing section:

    - StellarPath analyses the class-signature pathway-specific PSNs to infer new information.
- Patient classification section:
    - StellarPath trains a graph convolutional network on each PSN.
    - The similarity is calculated with the significant molecules enriching a pathway, so the graph could be denoted with $\mathcal{G}PATHWAY$.
    - Each PSN reflects how patients are similar according to how they regulate a pathway.

To improve the clarity of our method, we want to remove any doubt about the PSN creation process early in the text, ensuring readers understand how the PSNs are built from the beginning. So, we strengthen the “Overview” section with the following revision:

- Overview section:
    - StellarPath builds pathway-specific PSNs. For each enriched pathway, StellarPath uses a novel similarity to assess how much each pair of training patients is similar. The similarity evaluates how much the patient’s values in the significant molecules of the pathway are close and similarly deregulated. The similarities are then used to build one PSN that represents the relationships between patients in one specific pathway.

Additionally, we recognize that the 'Computational resources' section may be considered the least interesting and most technical part of our manuscript for many readers. Therefore, we position it at the end of the "Results" section. This adjustment aims to prioritize the presentation of the other more significant and interesting findings.

Reviewer 1 Comment 14:

The resolution of most images is insufficient. In Figure 6 (C), the color of edges is difficult to distinguish. It is recommended to modify Figure 6 (C) and add graphics of important PSNs.

Reply:

We appreciate your feedback regarding the resolution and clarity of our images, specifically Figure 6(C). The issue is likely to stem from the image compression applied by Word. We included the images directly in the Word document to facilitate the review process and reading, acknowledging that the journal will use the native versions for the publication.

Precisely, we now also processed our images with Preflight Analysis and Conversion Engine (PACE) digital diagnostic tool, https://pacev2.apexcovantage.com. PACE that ensures that figures meet PLOS requirements. Plus, for this revision, the editors also checked and approved the images of our manuscript.

Having said that, it is likely not a problem of resolution but an issue that may arise from the size of certain elements, such as the edges in Figure 6 (now Figure 5 due to Rev1 comment 13), as you highlighted.

Therefore, we increase the size of the edges in Figure 6 (now Figure 5 due to Rev1 comment 13), and we also add a new file called Supplementary 4 that contains the plots of predictive PSNs related to the classification of the CLL patients. By doing this, we also update the discussion of

Figure 6 (C) to highlight patients that are important both in the (B Cell Receptor Signaling Pathway) PSN of Figure 6 (C) and one (negative regulation of T cell activation) of the PSNs included in the new Supplementary 4.

Reviewer 1 Comment 15:

There are some grammatical issues in the manuscript, for instance, 'The Separability Power (Method 2.4) and the Jaccard Index.' Additionally, certain expressions, like 'aka,' are not suitable for a research paper. It is advisable to revise the manuscript carefully.

Reply:

Thank you for the comment.

We have now completely revised the "Topology of patient similarity networks" section where you found the grammatical issues.

We revised the usage of "aka" in the text. We replaced it with "also known as". For example in the "Patient similarity networks" section, the first component, also known as Stability Component, divides the smallest value that each molecule has in the two patients by its largest value.

We checked the entire manuscript and corrected the grammar issues that we managed to find.

Reviewer 1 Comment 16:

There are some formatting irregularities, including the pseudocode format for the GraphSAGE algorithm and the format of tables. Furthermore, there is inconsistency in the font styles of many variables throughout the entire manuscript, and it is recommended to use italics for all variables. It is advisable to revise the manuscript carefully.

Reply:

Thank you, a lot, for this feedback.

The pseudocode of the GraphSAGE algorithm has been moved from the manuscript to the supplementary material 1.8 as a consequence of comment 6. To avoid possible ambiguities within the pseudocode, we add parenthesis defining the start and end of each loop for.

Regarding the font of variables, we indeed previously formatted single variables as Equations and the format of some Equations was weirdly different from the format of other Equations. Now single variables are normal text formatted in Italic and equations have all the same format (MathType + Italic). These changes align with your comment and with PLOS guidelines:

- MathType/Equation Editor for display and inline equations
- Single variables (e.g., "$a^2 + b^2 = c^2$"), Greek or other symbols (e.g., β, Δ, or ′ [prime]), or mathematical operators (e.g., x, ≥, or ±) as normal text.

Reviewer 1 Comment 17:

It is hard to understand some descriptions in the Methods section because there are some contradictions when explaining the details of StellarPath. Hope the author will check the inconsistency. Specifically, in the last paragraph of the Feature selection section, the author described the standard for selecting activated/inhibited pathways. However, in the next section, it mentioned,

‘Conversely, if the pathway is inhibited (i.e., negative expression log fold change of its molecules),’.

Reply:

Your feedback on the Methods section's clarity regarding activated and inhibited pathways in StellarPath is well appreciated. We acknowledge the discrepancy in the "Patient similarity networks" section, where the explanation of the inhibited pathway was overly simplified and did not align with the detailed description in the "Feature selection" section.

To resolve this confusion, we remove the misleading simplification related to inhibited pathways. Additionally, we ensure consistency across the manuscript, so the definition and meaning of activated and inhibited pathways remain unchanged from how they are presented in the "Feature selection" section.

Reviewer 1 Comment 18:

In the Abstract, the author mentioned that 17 datasets were used when testing StellarPath's computing resources and classification performances, but only 16 datasets were shown in the figures presenting the result these two experiments. It is suggested to check for this conflict.

Reply:

In our study, 16 datasets have been used to evaluate the classification performance and computational efficiency of StellarPath and netDx, as well as to compare the resulting pathways and PSNs of their analysis. These datasets are discussed in the Result's first four subsections.

Additionally, 1 dataset including Chronic Lymphocytic Leukemia (CLL) patients derived from four distinct studies, has been used to assess StellarPath's classification and inference capabilities, discussed in the "Inference" section.

To address this apparent discrepancy, we clarify the distinct purposes of the datasets within the Abstract.

Reviewer 1 Comment 19:

It is best to provide exact data and images as evidence for the following statement, ‘In conclusion, GSEA finds many more pathways than StellarPath, some of them are clearly connected to OGD but many of them are generic or seemingly unrelated to the classes in comparison.

Reply:

Thank you for your suggestion. We enhance the discussion regarding the pathways identified by GSEA, focusing on those not directly related to the Oxygen-Glucose Deprivation (OGD) response. A new supplementary section (1.14) now provides detailed explanations for pathways unconnected to OGD, alongside an updated Supplementary Table (2.8) that highlights these pathways. These additions aim to clarify the relevance of each pathway to OGD, ensuring readers can easily discern which cellular functions are not associated with the OGD condition.

Reviewer 1 Comment 20:

The formula for the Stability Component is most likely written incorrectly. The summation symbol should be outside the semicolon.

Reply:

Upon reviewing the formula of the Stability Component, we confirm its correctness as written in the manuscript. The summation symbol, which iterates over the variable 'i', could be potentially written only one time for both the 'min' and 'max' functions but it would not change the outcome.

Reviewer 2 Comment 1:

Clarity and Structure: The introduction effectively sets the stage for the significance of patient classification and the limitations of existing methods. However, the introduction could benefit from a more explicit statement of the specific challenges that StellarPath aims to address.

Reply:

Thank you for the very interesting comment. The introduction is a bit long and the reader may lose the challenges that StellarPath aims to address.

We add a new paragraph in the Introduction section that summarizes the following challenges:

1. Meaningful features: Identifying relevant clinical and biological features for meaningful patient classification. Avoiding the classifier to use predictive but biologically meaningless features.
2. Integration of Multiple Omics: Effectively integrating diverse omics to mirror a biologist's expertise in hierarchically analyzing patient data.
3. Interpretability: Ensuring PSNs are interpretable and reflective of biologically significant patterns, helping the user to understand the method’s rationale behind feature selection, class differentiation and patient classification.
4. New Insights: Producing predictive PSNs that not only classify patients but also enable the retrieval of new meaningful insights for further analyses such as patient stratification, outlier detection, and correlation with clinical data.
5. Fast and Versatile Implementation: Overcoming the drawbacks prevalent in existing classifiers, such as the need for extensive user input, hyper-parameter tuning, and high computational resources, while maintaining simplicity, efficiency, and the use of an interpretable, omics-specific similarity measure.

Reviewer 2 Comment 2:

Interpretability and Clinical Relevance: The manuscript emphasizes the interpretability of StellarPath, which is a significant strength of the method. However, further discussion on how the interpretability of patient similarity networks translates to actionable insights for clinicians and researchers would enhance the clinical relevance of StellarPath.

Cover letter

Reply:

This feedback is nice and aligns with multiple comments made by reviewer 1. We suggest to read the replies to the reviewer's comments 3 and 5.
